# Fungal Metabolites Antagonists towards Plant Pests and Human Pathogens: Structure-Activity Relationship Studies

**DOI:** 10.3390/molecules23040834

**Published:** 2018-04-05

**Authors:** Marco Masi, Paola Nocera, Pierluigi Reveglia, Alessio Cimmino, Antonio Evidente

**Affiliations:** Dipartimento di Scienze Chimiche, Università di Napoli Federico II, Complesso Universitario Monte Sant’Angelo, Via Cintia 4, 80126 Napoli, Italy; paola.nocera@unina.it (P.N.); pierlugi.reveglia@unina.it (P.R.); alessio.cimmino@unina.it (A.C.); evidente@unina.it (A.E.)

**Keywords:** fungal secondary metabolites, bioactive natural products, fungicides, bactericides, insecticides, herbicides, SAR studies

## Abstract

Fungi are able to produce many bioactive secondary metabolites that belong to different classes of natural compounds. Some of these compounds have been selected for their antagonism against pests and human pathogens and structure–activity relationship (SAR) studies have been performed to better understand which structural features are essential for the biological activity. In some cases, these studies allowed for the obtaining of hemisynthetic derivatives with increased selectivity and stability in respect to the natural products as well as reduced toxicity in view of their potential practical applications. This review deals with the SAR studies performed on fungal metabolites with potential fungicidal, bactericidal, insecticidal, and herbicidal activities from 1990 to the present (beginning of 2018).

## 1. Introduction

Pests are one of the main concerns for plants, animals, and humans. Among them, invasive species such as weeds are a serious problem for agriculture while organisms that are human disease carriers such as mosquitoes cause significant diseases or even death, worldwide [1,2]. Strategies of pest management can vary but are still strongly dependent on the use of synthetic chemicals. The intensive use of these pesticides in the last fifty years has had negative environmental and ecological impacts [3,4,5]. The accumulation of the chemicals in the ecosystem and food and the rapidly evolving resistance of pests to commercial pesticides are two main problems to be solved [6]. Furthermore, many substances have been banned because of their hazardous effects and chemical companies have difficulties substituting them with safe and efficient synthetic compounds [7,8]. Thus, there is an increased requirement for novel and environmentally friendly alternatives and biological control could be an efficient tool for pest management [9,10]. Biocontrol agents are categorized into four groups: macroorganisms (for example, predators, parasitic insects, and nematodes), micro-organisms (for example, bacteria, fungi, and viruses), chemical mediators (for example, pheromones) and natural substances (originated from plant or animal). Biopesticide products can be either living organisms, and more specifically micro-organisms, or products derived from living organisms, including the metabolites produced during their growth and development [11]. Biopesticides are natural product based, therefore, they are expected to be more environment-friendly [12]. Furthermore, the half-life of biopesticides is usually shorter than that of chemicals but their activity spectrum should be carefully evaluated [13]. The available data show that natural products have had a substantial impact on pest control by providing compounds that are efficient as pest control agents. More importantly, identifying novel modes of action serves as inspiration/models for synthetic pesticides [6,14]. However, research, development, and regulation are necessary to improve the number of effective solutions on the bioherbicide world market [11]. Natural products have also been extensively studied to discover new drugs and to develop new formulations [15,16,17,18,19].

The antimicrobial resistance developed by human pathogens is one of the most urgent public health problems. For instance, members of the *Enterobacteriaceae* and *Pseudomonas* families—organisms that cause diarrhea, urinary infection, and sepsis—are now virtually resistant to all of the traditional antibiotics [20,21,22]. Bacteria can resist antibiotics by different mechanisms: chromosomal mutation; inductive expression of a latent chromosomal gene; by exchanging genetic material through transformation and/or transduction (bacteriophage); and through conjugation by plasmids. After a bacterium gains a gene resistance to protect itself from various antimicrobial agents, they can use several biochemical types of resistance mechanisms [23]. Several drugs that have recently been developed are still unable to overcome the resistance mechanisms of these pathogens [24,25].

New efforts are now dedicated to the discovery of new compounds with different mechanisms of action and in this battlefield, the biologically active natural products isolated from living organisms could play a fundamental role, being an efficient alternative to synthetic or semi-synthetic compounds [23,24,25,26,27,28]. 

Among the living organisms, fungi and bacteria are able to produce secondary metabolites with interesting biological activities [2,29,30]. These metabolites belong to all the different classes of natural compounds, such as terpenes, phenylpropanoids, polyketides, alkaloids, and so forth. [31,32]. Thus, these organisms represent a very good source of natural substances that could be used as an efficient tool to design natural biopesticides or antibiotics against human pathogens with new modes of action. Some selected metabolites have been used to carry out structure–activity relationship (SAR) studies to better understand which structural features are essential for their biological activity, to increase the selectivity and the stability of the natural products, as well as to reduce their toxicity in view of their potential practical applications.

This review reports the results of SAR studies performed from 1990 to the present, using fungal metabolites with potential practical applications as potential fungicides, bactericides, insecticides, and herbicides (Table S1, Supplementary Materials). The herbicides section is focused on the last results on SAR studies which were not covered by the review by Cimmino et al. 2015 [2].

## 2. Structure–Activity Relationship Studies Performed with Fungal Metabolites

### 2.1. Fungicides

**Figure 1:**
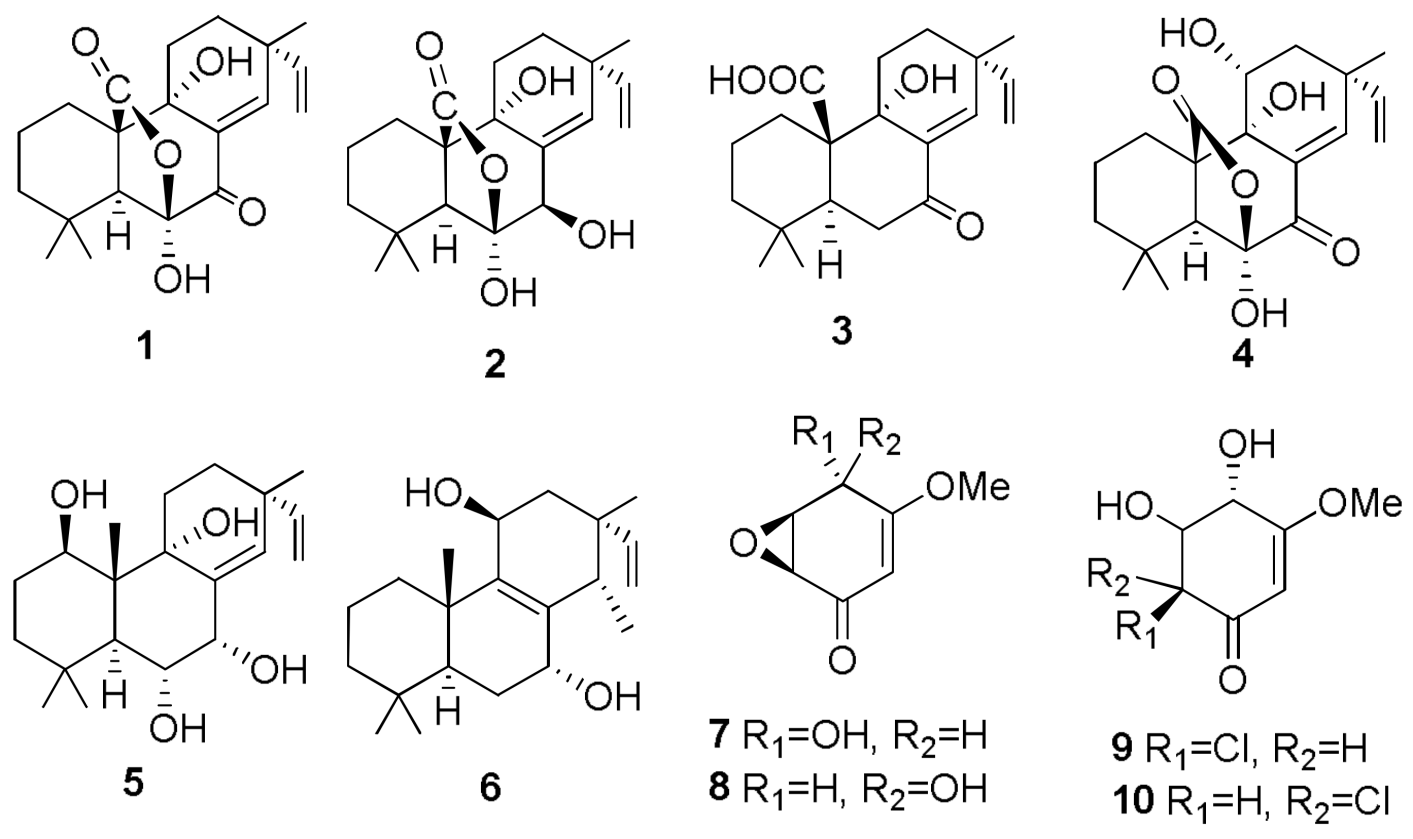
The structures of sphaeropsidins A–F (**1**–**6**) and sphaeropsidones (**7** and **8**) and their chlorinated analogs (**9** and **10**).

Fungi are by far the most prevalent plant pathogens and several studies have been carried out to develop management strategies that minimize crop losses. Many natural products have shown potential fungicidal activity against phytopathogenic fungi and some of them have been selected to perform SAR studies. In particular, pathogenic fungi of forest trees are a good source of bioactive metabolites which have been extensively studied [33]. From the fungus *Diplodia cupressi*, involved in the cypress (*Cupressus sempervirens*) canker disease in the Mediterranean area, tri-, and tetra-cyclic un-rearranged pimarane diterpenes, namely sphaeropsidins A–F (**1**–**6**, Figure 1), were isolated together with the dimedone methyl ethers named sphaeropsidones (**7** and **8**, Figure 1) and their chlorinated analogs (**9** and **10**, Figure 1) [33].

**Scheme 1:**
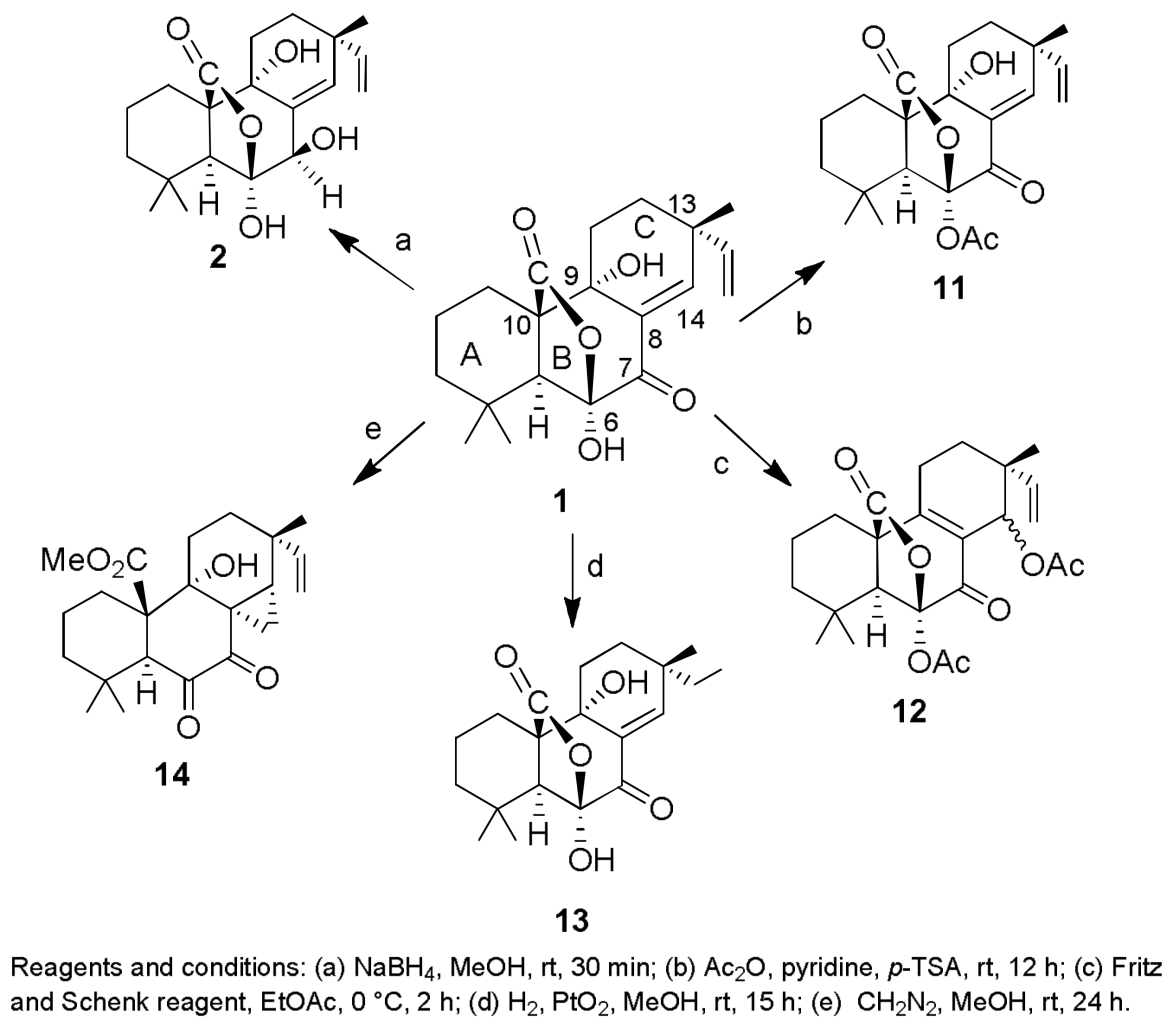
The structures of sphaeropsidin A (**1**) and its derivatives (**2**, **11**–**14**).

**Scheme 2:**
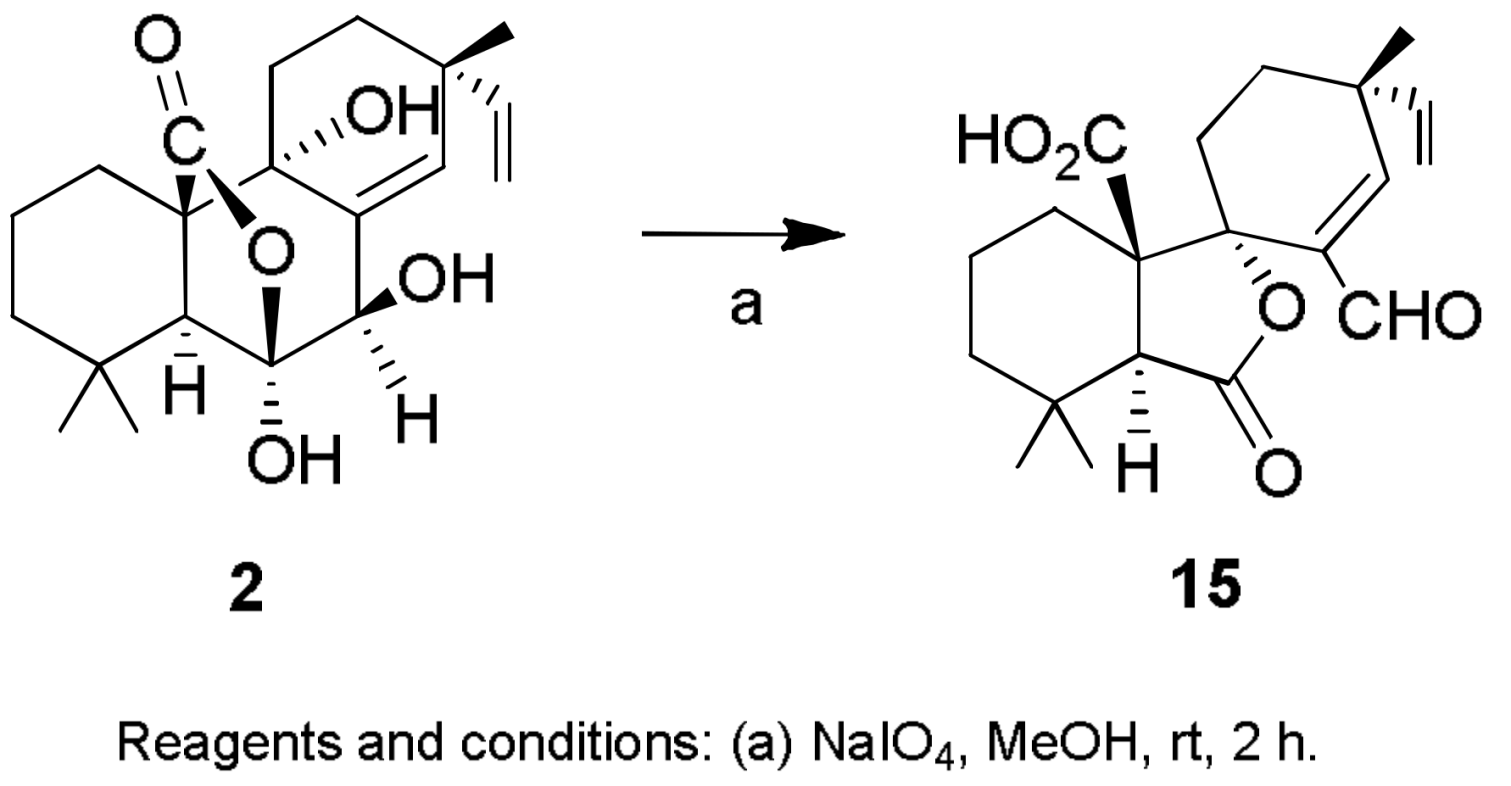
The structures of sphaeropsidin B (**2**) and its derivative (**15**).

**Scheme 3:**
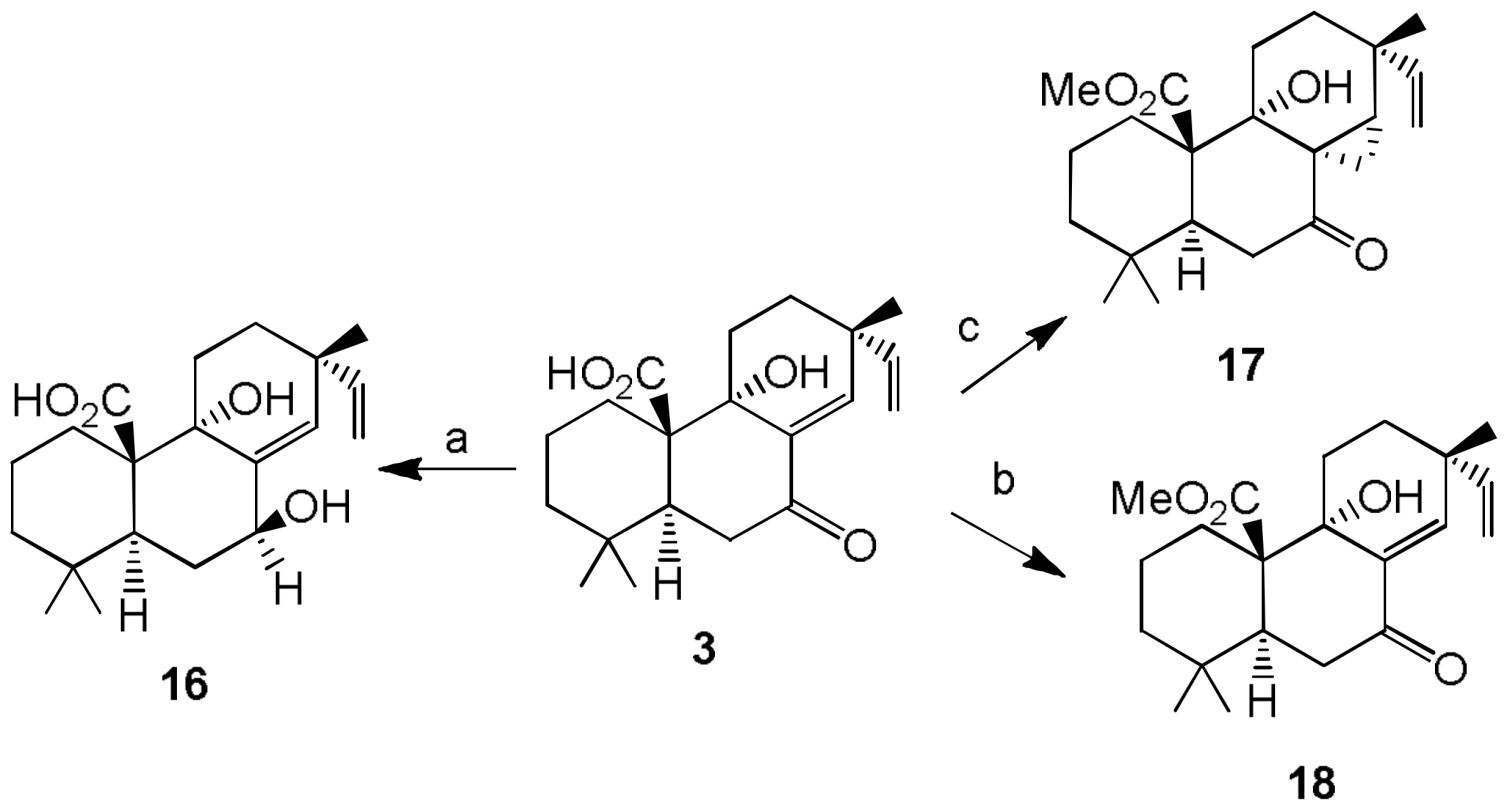
The structures of sphaeropsidin C (**3**) and its derivatives (**16**–**18**).

Sphaeropsidin A is a well-known bioactive metabolite produced by different species of *Diplodia* [34,35,36,37] which showed strong anticancer activity against advanced melanomas [38,39]. Recently, its absolute configuration was confirmed by X-ray analysis of the toxin and its 6-*O*-*p*-bromobenzoyl derivative [40], while its anticancer activity was evaluated in combination with cytotoxic chemotherapeutics [41]. Sphaeropsidins A–C (**1**–**3**) showed a preliminary interesting activity when tested against several plant pathogenic fungi, including *Seiridium cardinale* and *Seiridium cupressi*, both agents of a different canker forms [42,43]. For this reason, their potential antimycotic activity was investigated [44]. In order to get information on the SAR and to identify which structural features are essential for the biological activities of sphaeropsidins, eight derivatives (**11**–**18**, Scheme 1, Scheme 2 and Scheme 3) were prepared by chemical transformation of the functionalities present in **1**–**3** [44].

A suitable amount of sphaeropsidin B was obtained by stereoselective reduction of **1** as reported in Scheme 1 [44].

The antimycotic activity of **1**–**6** and **11**–**18** was assayed at 100 μg/mL on eight plant pathogenic fungi, namely *Botrytis cinerea*, *Fusarium oxysporum*, *Penicillium expansum*, *Verticillium dahliae*, *Phomopsis amygdali*, *S. cardinale*, *S. cupressi*, and *Seiridium unicorne.* The results showed that the integrity of the tricyclic pimarane system, the preservation of the double bond from C-8 to C-14, the tertiary hydroxyl group at C-9, the vinyl group at C-13, the carboxylic group at C-10, and the integrity of the A-ring are structural features essential to impart activity against several plant pathogenic fungi [44].

These relations were also observed when the same compounds were tested (at a concentration of 100 μg/mL) to evaluate their phytotoxic activity against host (three cypress species) and non-host plants [44].

**Scheme 4:**
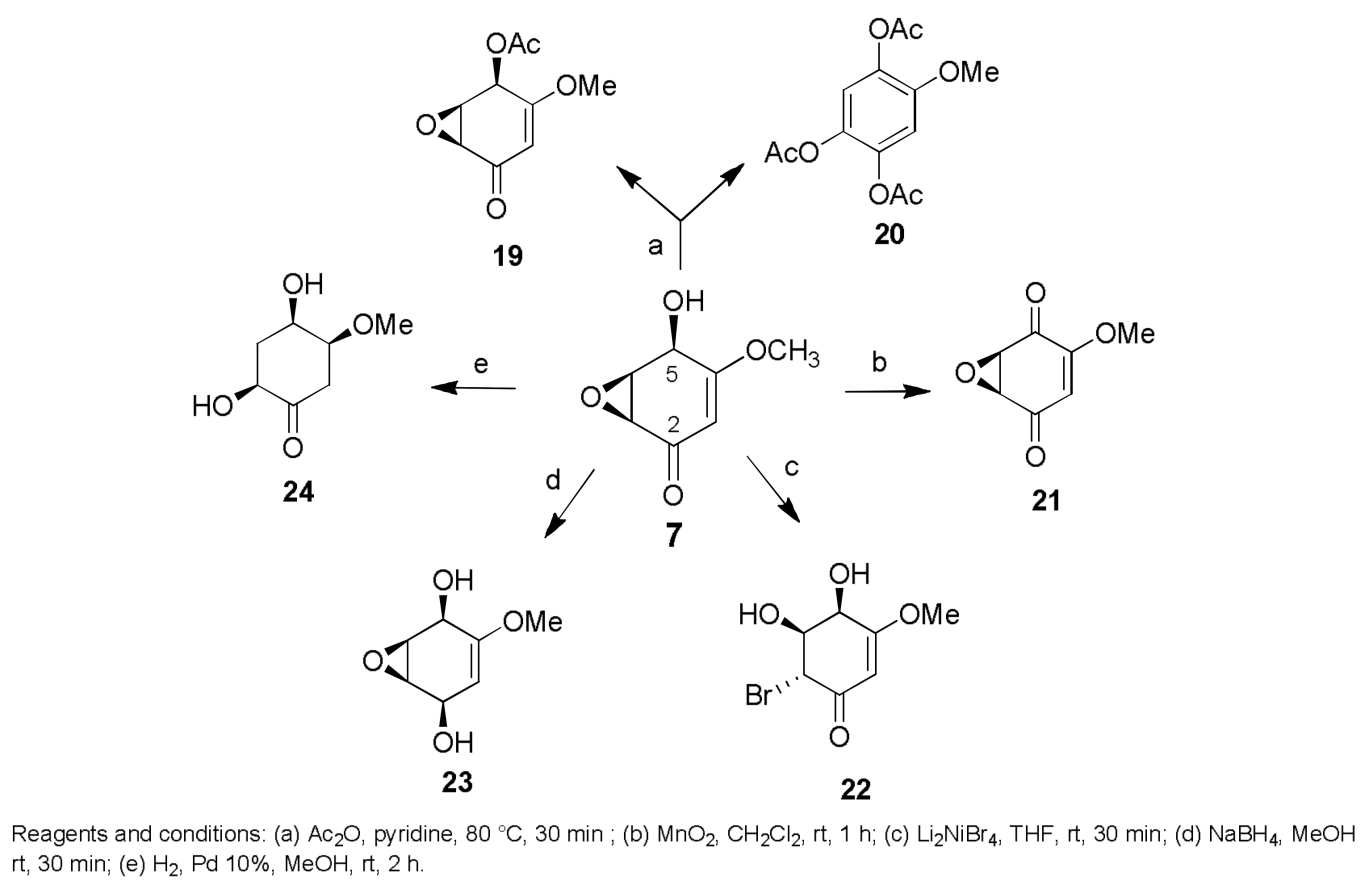
The structures of sphaeropsidone (**7**) and its derivatives (**19**–**24**).

**Scheme 5:**
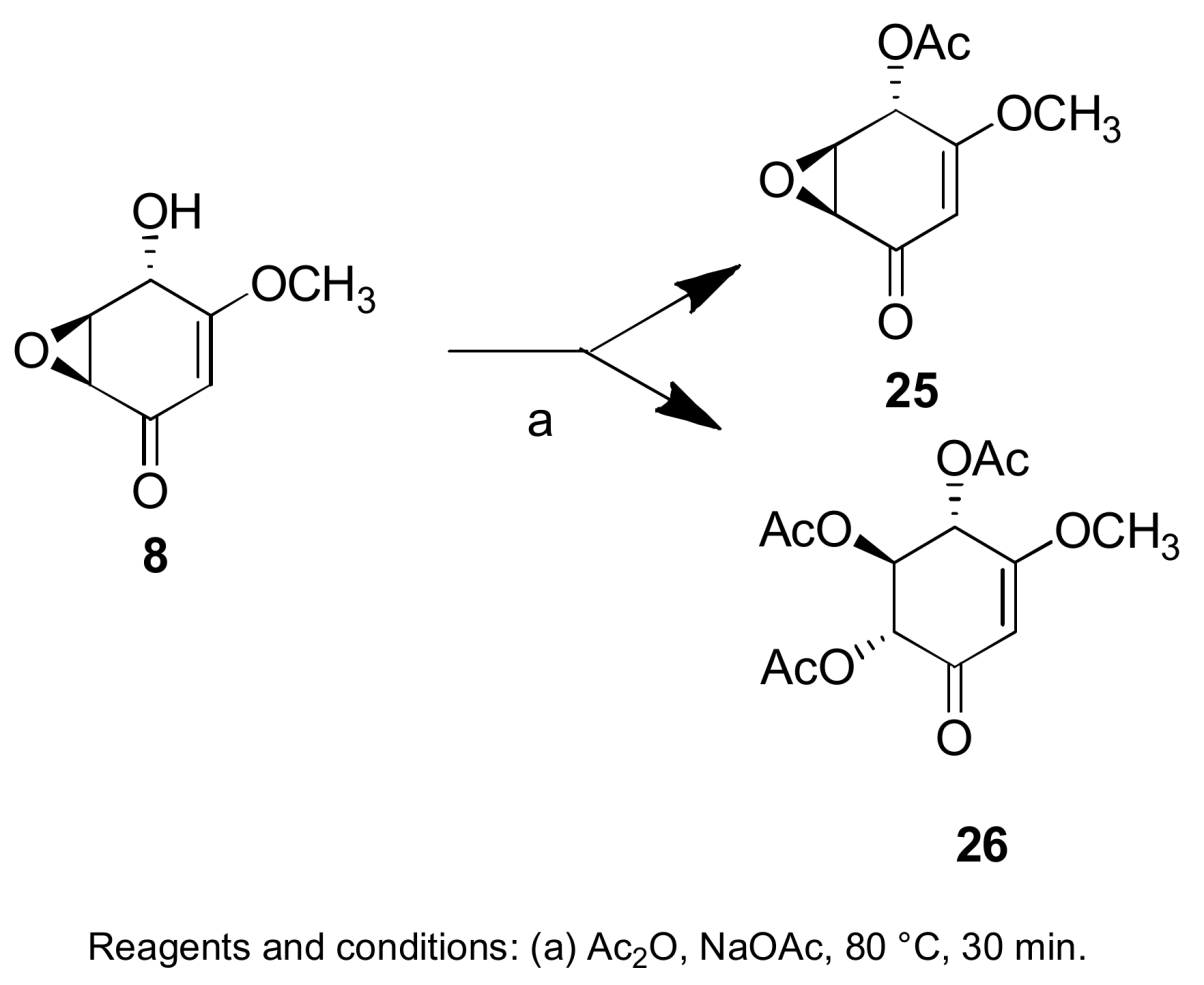
The structures of *epi*-sphaeropsidone (**8**) and its derivatives (**25** and **26**).

Sphaeropsidones (**7** and **8**, Figure 1) and their chlorinated analogs (**9** and **10**, Figure 1) were evaluated for their activities against five *Phytophthora* species (destructive pathogens of forest trees and shrubs) and for their phytotoxicity on *Quercus ilex*, *Quercus rubra*, *Quercus suber*, and tomato (*Lycopersicon esculentum*) leaves. In particular, eight derivatives (**19**–**26**, Scheme 4 and Scheme 5) were prepared by chemical transformation of the functionalities present in **7** and **8** in order to carry out SAR studies [45].

The results obtained with either natural or synthetic analogs of sphaeropsidones (tested at 0.05, 0.1 and 0.2 mg/plug) showed that the specific structural features related to their toxicity are the C-5 hydroxy group, the epoxy ring, and the C-2 carbonyl group, along with the C-5 absolute configuration. In fact, compounds that were much less active and/or inactive in comparison to **7** were obtained with the opening of the epoxy ring and with the modifications of the C-5 hydroxy group and the reduction of the C-2 carbonyl group. However, the most important result was obtained with compound **21** which, among the synthesized derivatives, was found to be more effective than **7** in inhibiting the mycelial growth of *Phytophthora* species. In particular, its activity was very similar to that showed by the synthetic fungicide (mefenoxam) commonly employed for the control of diseases caused by oomycetes. Considering the development of a resistance to the phenylamides and the fact that the *Phytophthora* species are invasive pathogens on a global scale, compound **21** could be suitable for the development of an alternative strategy to manage these pathogens [45].

**Figure 2:**
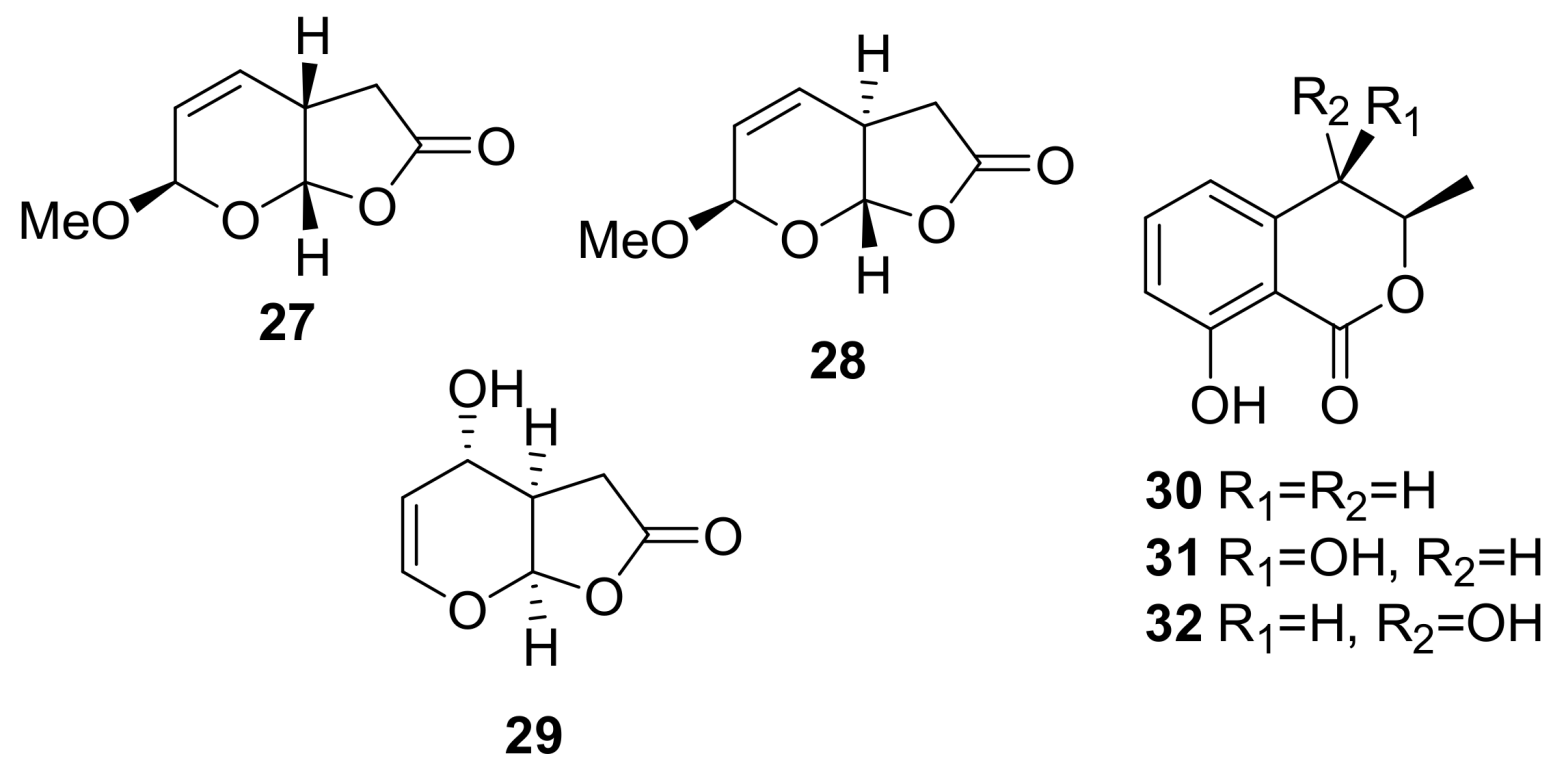
The structures of afritoxinones A and B (**27** and **28**), oxysporone (**29**), *R*-(−)-mellein, (3*R*,4*R*)-4-hydroxymellein, and (3*R*,4*S*)-4-hydroxymellein (**30**–**32**).

**Scheme 6:**
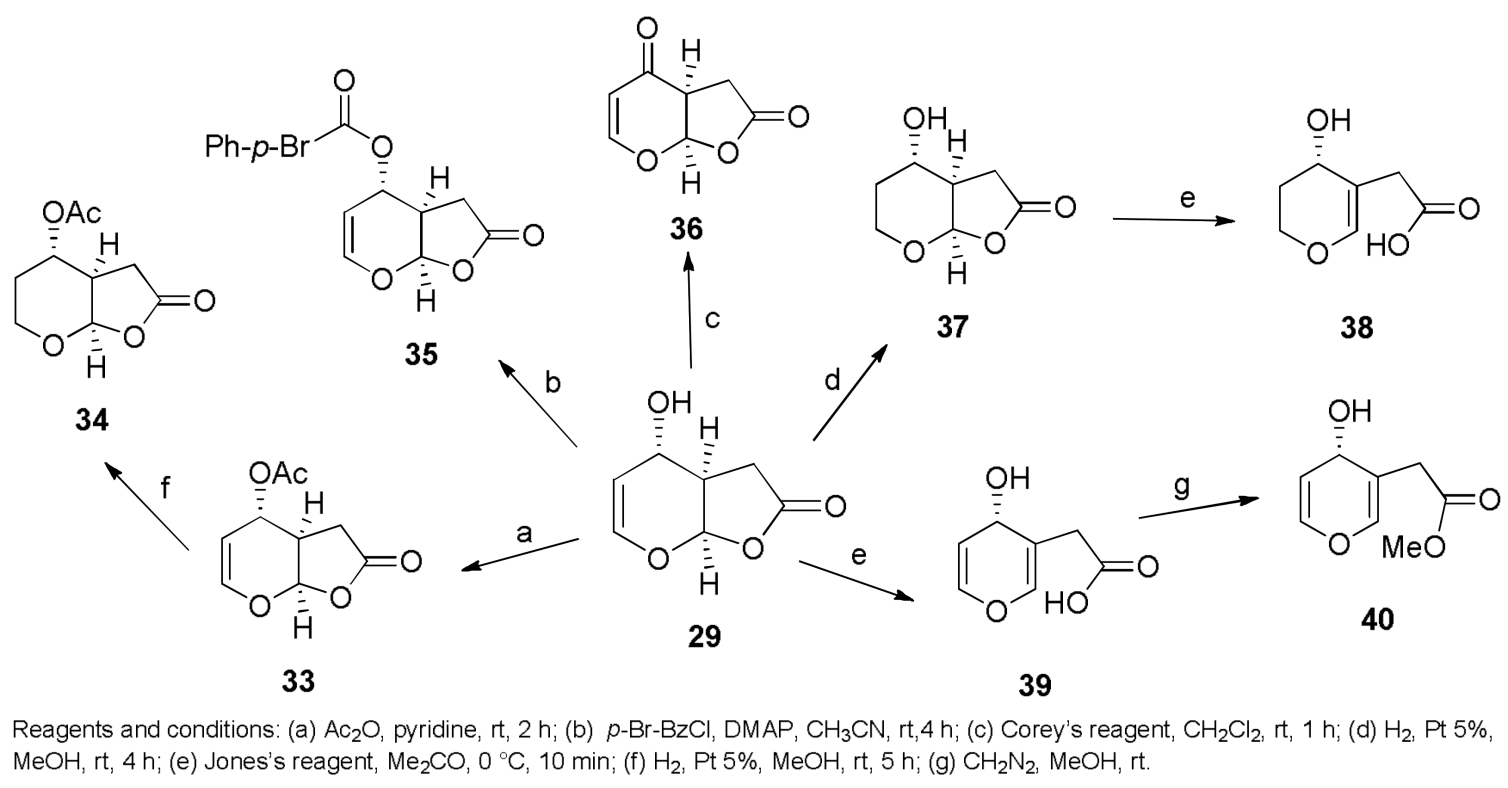
The structures of oxysporone (**29**) and its derivatives (**33**–**40**).

From *Diplodia africana*, the fungal pathogen responsible for branch dieback of *Juniperus phoenicea* in Italy, two phytotoxic dihydrofuropyran-2-ones, named afritoxinones A and B (**27** and **28**, Figure 2) were isolated together with the known oxysporone (**29**, Figure 2), sphaeropsidin A and *epi*-sphaeropsidone (**1** and **8**, Figure 1), *R*-(−)-mellein, (3*R*,4*R*)-4-hydroxymellein, and (3*R*,4*S*)-4-hydroxymellein (**30**–**32**, Figure 2) [34]. The phytotoxic activity of afritoxinones A and B and the main compound oxysporone was evaluated on host (*J. phoenicea*) and non-host plants (*Q. ilex*, *Q. suber*, and tomato) by cutting and leaf puncture assays. Oxysporone proved to be the most phytotoxic compound [34]. Successively, eight derivatives (**33**–**40**, Scheme 6) were hemisynthesized and assayed for their phytotoxic and antifungal activities in comparison to the parent compound oxysporone. In particular, oxysporone (**29**) and its derivatives (**33**–**40**) were tested (at a concentration of 100 mg/mL) on four different plant pathogens including two fungal species (*Athelia rolfsii* and *Diplodia corticola*) and two oomycetes (*Phytophthora cinnamomi* and *Phytophthora plurivora*) which have a great impact in both agriculture and natural ecosystems. The same compounds were also tested (at a concentration of 1 mg/mL) against non-host plants, namely cork oak (*Q. suber*), holm oak (*Q. ilex*), and grapevine (*Vitis vinifera*) to evaluate their phytotoxic activity. The results of the latter assay showed that the dihydrofuropyranone carbon skeleton and both the double bond and the hydroxy group of the dihydropyran ring are structural features important in conferring phytotoxic activity [46].

Unfortunately, the antifungal activity data were not suitable to speculate on the SAR but the corresponding 4-*O*-benzoyl derivative of oxysporone **36** showed a good antifungal activity towards *P. cinnamomi*, *P. plurivora*, and *A. rolfsii*. However, its EC_50_ (concentration which inhibits mycelial growth by 50%) was one or two orders of magnitude lower than those of three specific commercial fungicides toclofos-methyl, pentachloronitrobenzene (PCNB), and metalaxyl-M [46]. 

**Figure 3:**
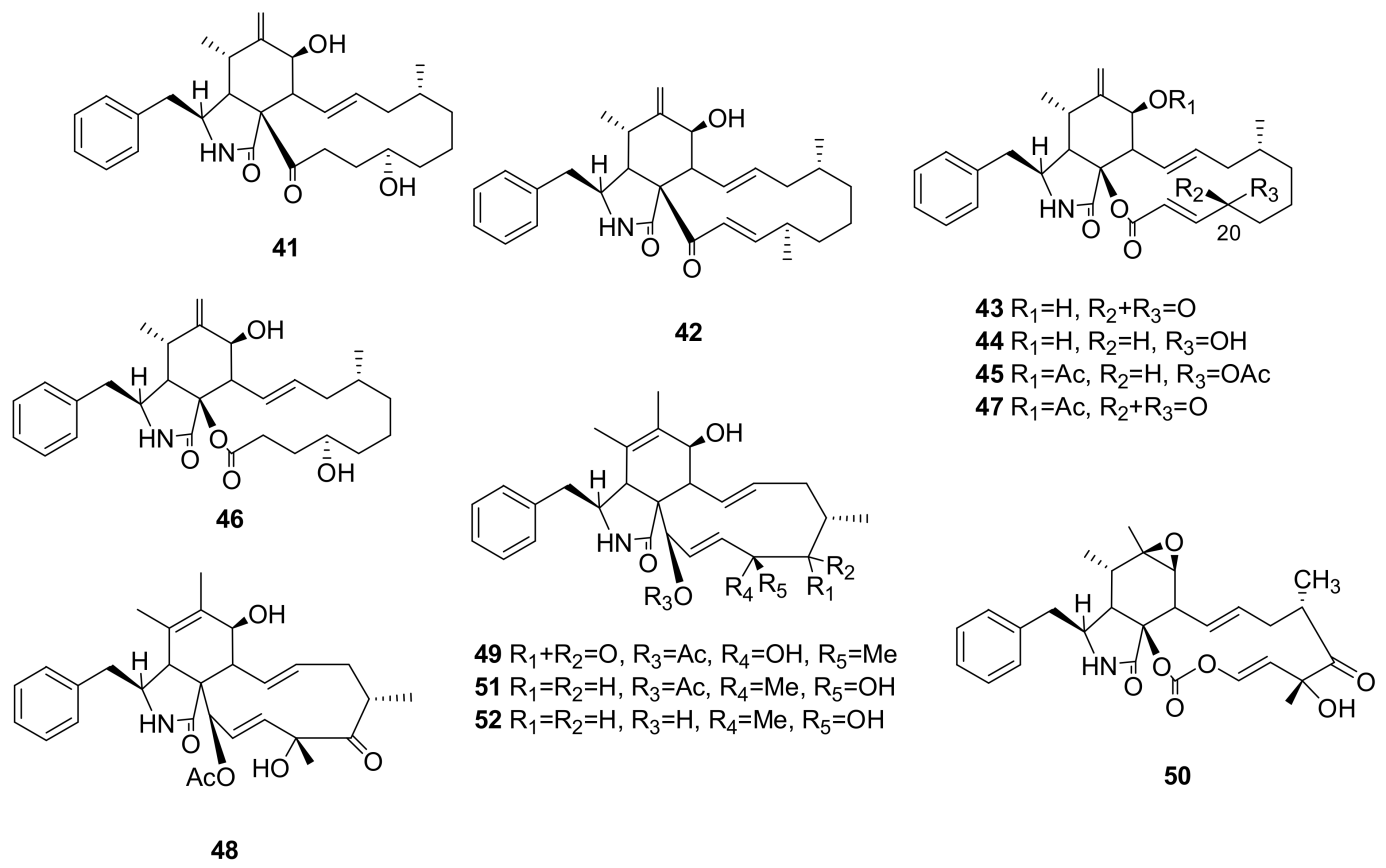
The structures of ascochalasin (**41**); deoxaphomin (**42**); cytochalasin A (**43**); cytochalasin B (**44**); 7,20-*O*,*O*′-diacetylcytochalasin B (**45**); 21,22-dihydrocytochalasin B (**46**); 17-*O*-acetylcytochalasin A (**47**); and cytochalasins C, D, E, H, and J (**48**–**52**, respectively).

From the culture filtrates of the fungus *Ascochyta heteromorpha*, the causal agent of a foliar disease of oleander (*Nertum oleander*), a new cytochalasin named ascochalasin (**41**, Figure 3), was isolated together with deoxaphomin (**42**, Figure 3) and cytochalasins A and B (**43** and **44**, Figure 3). Cytochalasins are a large group of fungal metabolites produced by several genera of fungi which showed different biological activities. These compounds have the ability to bind to actin filaments and block the polymerization and the elongation of actin [47,48,49,50].

These compounds were assayed against *Geotrichum candidum* together with two derivatives of cytochalasin B (namely 7,20-*O*,*O*′-diacetylcytochalasin B and 21,22-dihydrocytochalasin B (**45** and **46**, Figure 3)); 17-*O*-acetylcytochalasin A (**47**, Figure 3); and the natural cytochalasins C, D, E, H, and J (**48**–**52**, Figure 3). However, only cytochalasin A (**43**) showed to be active (<5 μg/disk) against this fungus, suggesting the importance of the ketonic group at C-20 (probably involved in a Michael reaction) which plays a significant role in the induction of fungicidal activity [51].

**Figure 4:**
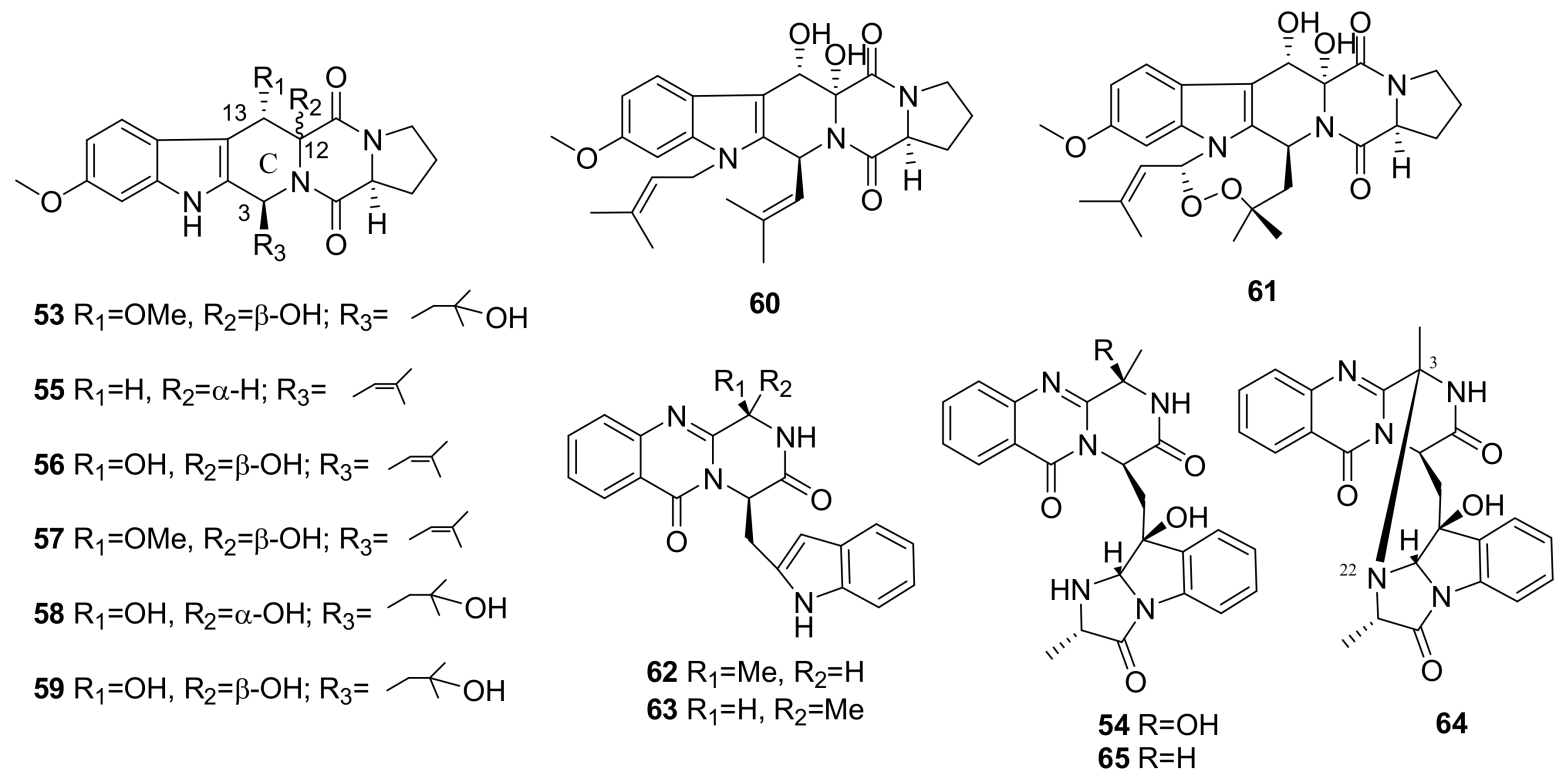
The structures of 12β-hydroxy-13α-methoxyverruculogen TR-2 (**53**); 3-hydroxyfumiquinazoline A (**54**); fumitremorgin C (**55**); cyclotryprostatins A (**56**) and B (**57**); verruculogen TR-2 (**58**); 12β-hydroxyverruculogen TR-2 (**59**); fumitremorgin B (**60**); verruculogen (**61**); and fumiquinazolines F, G, D, and A (**62**−**65**).

*Aspergillus fumigatus* LN-4, an endophytic fungus isolated from the stem bark of *Melia azedarach*, was grown in vitro to evaluate its ability to produce secondary metabolites with antifungal activity. In particular, from its fermentation broth, 39 fungal metabolites were isolated, including the two new alkaloids 12β-hydroxy-13α-methoxyverruculogen TR-2 (**53**, Figure 4) and 3-hydroxyfumiquinazoline A (**54**, Figure 4). When tested against some phytopathogenic fungi (*B. cinerea*, *Alternaria solani*, *Alternaria alternata*, *Colletotrichum gloeosporioides*, *Fusarium solani*, *Fusarium oxysporum* f. sp. *niveum*, *Fusarium oxysporum* f. sp. *vasinfectum*, and *Gibberella saubinettii*), 16 compounds showed potent antifungal activity. For some compounds, these results were comparable to those obtained testing two commercial fungicides such as carbendazim and hymexazol, which were used as a positive control. Structure–activity relationships of the metabolites were also discussed for the tested indole diketopiperazine alkaloids (**53**, **55**−**61**, Figure 4) and the fumiquinazolines (**54**, **62**–**65**, Figure 4) [52]. In particular, the results obtained with the diketopiperazine alkaloids suggested that the introduction of a MeO group onto C-13 in these molecules give higher activity, regardless of the configuration of the OH group at C-12. Compound **53** (MIC (Minimum inhibitory concentration) = 6.25 μg/mL), having a 2-methylpropan-2-ol group at C-3, is more toxic than **57** (MIC = 12.5 μg/mL), which has an isobutenyl group at C-3, indicating that the 2-methylpropan-2-ol substituent at C-3 on ring C of the compounds **53**, **58**, and **59** appear to be necessary for activity. Furthermore, comparing the activity of **60** and **61** shows that the presence of the peroxide bridge is important to impart antifungal activity. Among the five fumiquinazolines (**54**, **62**−**65**), fumiquinazolines F (**62**), G (**63**), A (**65**), and 3-hydroxyfumiquinazoline A (**54**) have good antifungal activities (MICs = 12.5−25 μg/mL), whereas, fumiquinazoline D (**64**) weakly inhibited the growth of phytopathogenic fungi (MICs = 25−50 μg/mL), indicating that the presence of a C−N bridge between C-3 and N-22 in compound **64** could be detrimental to the activity. In addition, the brine shrimp (*Artemia salina*) toxicity was determined and compounds **60** and **61** both showed significant toxicities with median lethal concentration (LC_50_) values of 13.6 and 15.8 μg/mL, respectively. Furthermore, among nine metabolites that were found to possess antifeedant activity against armyworm larvae, compounds **60** and **61** gave the best activity with antifeedant indexes (AFI) of 50.0% and 55.0%, respectively. All these results allowed the authors to consider the compounds 12β-hydroxy-13α-methoxyverruculogen TR-2 (**53**) and fumitremorgin B (**60**) as promising lead compounds for developing new fungicides [52]. However, considering the toxicity shown by **60**, its potential application as a natural fungicide seems very difficult. For the other compounds, further studies need to be carried out in order to better understand the mechanism of action associated with the antifungal and antifeedant effects and to show some selectivity. 

**Figure 5:**
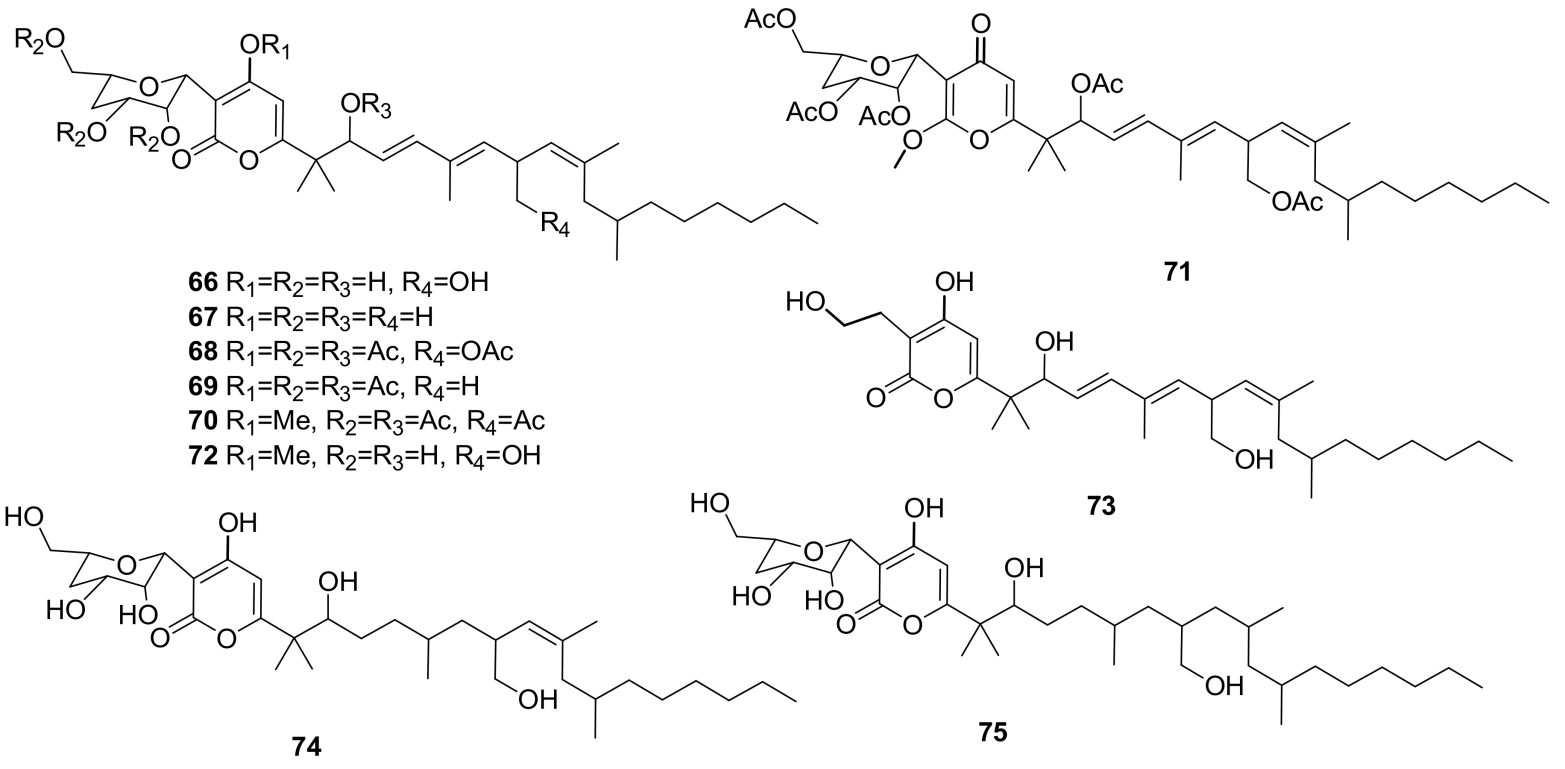
The structures of fusapyrone (**66**), its derivatives (**68**,**70**–**75**), deoxyfusapyrone (**67**), and its derivative (**69**).

From the rice cultures of the soil fungus *Fusarium semitectum* obtained from maize stalk rot in southern Italy, two 3-substituted 4-hydroxy-6-alkyl-2-pyrones were isolated and named fusapyrone (**66**, Figure 5) and deoxyfusapyrone (**67**, Figure 5), respectively. Fusapyrone showed a preliminary antifungal activity and low zootoxicity in an *A. salina* larvae mortality bioassay, while **67** had a higher zootoxicity [53]. Thus, a SAR study was performed by preparing seven different chemical derivatives of fusapyrone (**68**,**70**–**75**, Figure 5) and one derivative of deoxyfusapyrone (**69**, Figure 5) and testing their antifungal and zootoxic activities in comparison with the natural compounds. In particular, the antifungal activity was tested against two yeasts (*Pichia guilliermondii* and *Rhodotorula glutinis*) and against three filamentous fungi *B. cinerea*, *Aspergillus parasiticus* and *Penicillium brevi-compactum* that are agents of pre- and post-harvest plant diseases. The derivatives were prepared by trying to modify the glycosyl residue, the 2-pyrone ring, and the aliphatic chain of **66** and **67**. Among the derivatives of **68**–**65**, only compounds **72**, **74**, and **75** retained some activity against *B. cinerea*, while the others were inactive against the yeasts. However, the activity was shown only at the highest concentration (25–50 μg/mL). These results highlighted the importance of the hydrophilic sugar residue for the activity against *B. cinerea*. The same derivatives were also tested against *A. salina* (brine shrimp) to evaluate the zootoxic activity. In this case, the toxicity was related to the increased hydrophobicity of some derivatives [54].

**Figure 6:**
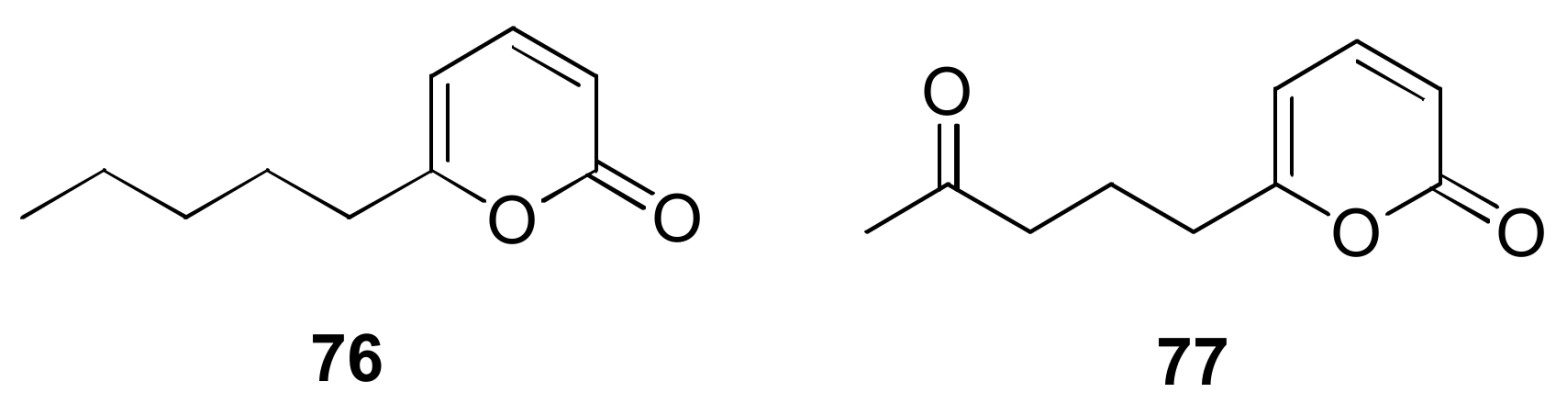
The structures of 6-*n*-pentyl-2*H*-pyran-2-one and viridepyronone (**76** and **77**)**.**

Considering the biological activities of **66** and **67** and the results obtained, the potential use of these molecules in combination with biocontrol agents for plant disease control was proposed [54]. The importance of α-pyrone moiety to impart antifungal activity in natural compounds is also confirmed by the fungicidal activity of 6-*n*-pentyl-2*H*-pyran-2-one (**76**, Figure 6), which is a metabolite produced by different *Trichoderma* species. In fact, compound **76** has been demonstrated to inhibit the growth of several plant-pathogenic fungi: *B. cinerea*, *Fusarium oxysporum* f. sp. *lycopersici*, *Fusarium verticillioides* (*moniliforme*), *Phytophthora megasperma*, *Rhizoctonia solani*, and *Armillaria mellea* [55,56]. Compound **76** was also previously isolated by some of the authors together with the new 6-substituted-2*H*-pyran-2-one, named viridepyronone (**77**, Figure 6), from the cultural filtrates of a *Trichoderma viride* strain showing in vitro antagonistic activity toward *Sclerotium rolfsii*, which is the causal agent of crown and stem rot of artichoke. Viridepyronone inhibited the growth of *S. rolfsii* by 48% with a MIC of 196 μg/mL [57].

**Scheme 7:**
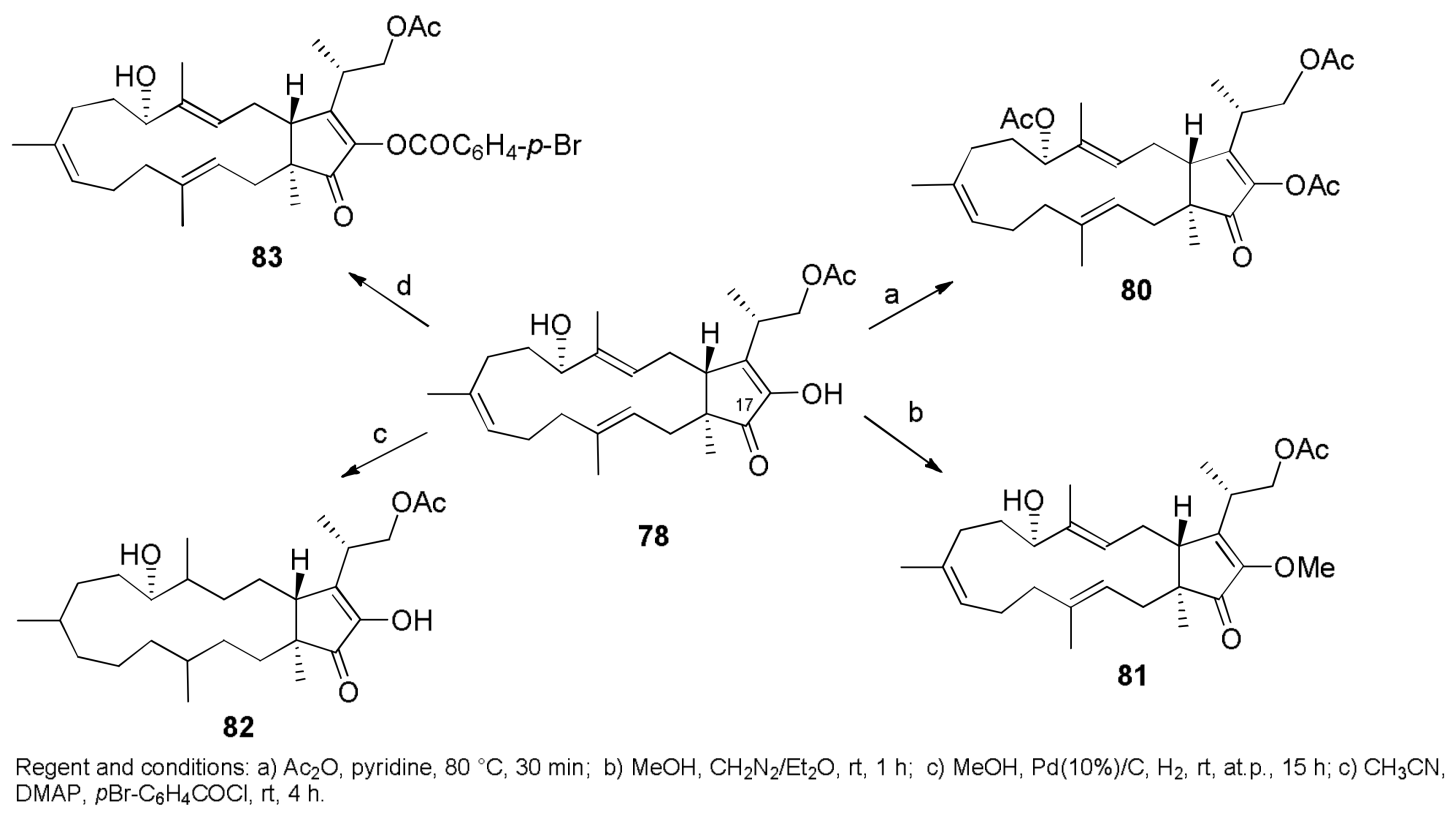
The structures of fusaproliferin (**78**) and its derivatives (**80**–**83**).

**Scheme 8:**
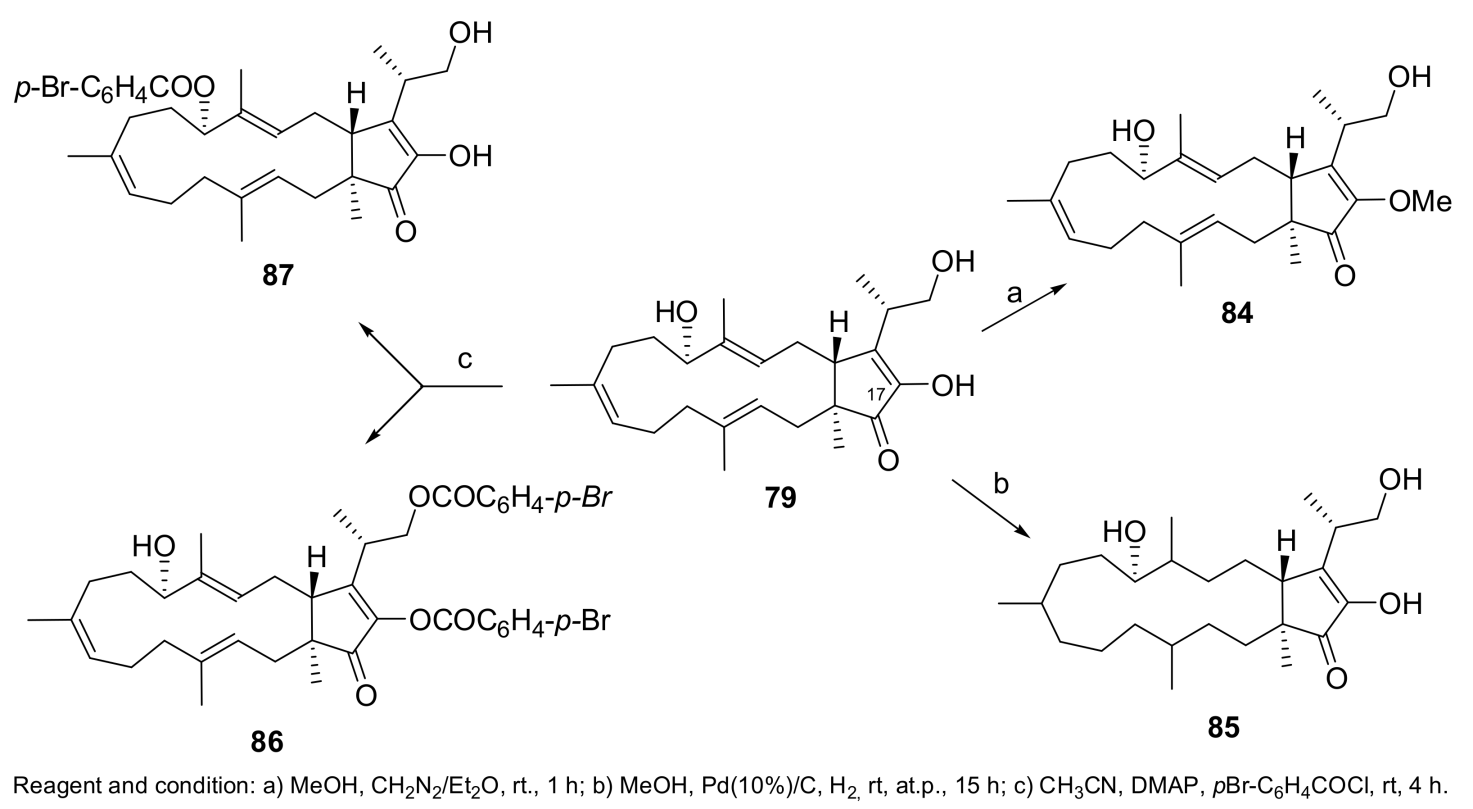
The structures of terpestacin (**79**) and its derivatives (**84**–**87**).

Dung-inhabiting fungi are also an underexplored reservoir of bioactive compounds [58]. Recently, the cultures of the two fimicolous fungi *Cleistothelebolus nipigonensis* and *Neogymnomyces virgineus* showed strong antifungal activity against *Alternaria brassicicola*, *B. cinerea*, and *Fusarium graminearum*. From the bioguided purification of their organic extracts, fusaproliferin (**78**, Scheme 7) and terpestacin (**79**, Scheme 8) were isolated and identified. A SAR study was conducted to understand the role of each functional group of these natural products to the imparted activity. In particular, four fusaproliferin derivatives (**80**–**83**, Scheme 7) and four terpestacin derivatives (**84**–**87**, Scheme 8) were prepared and tested (using a concentration of 10^−3^ M) against *A. brassicicola*, *B. cinerea*, and *F. graminearum* [59]. These results showed that the three fungi have different sensitivities towards fusaproliferin and terpestacin. Furthermore, the hydroxy enolic group at C-17 and the conformational freedom of the macrocyclic ring, due to the presence/absence of the three double bonds, are structural features important to impart activity [59].

However, fusaproliferin resulted in being toxic to *A. salina*, against the lepidopteran cell line SF-9, and also against the human non-neoplastic B-lymphocyte cell line IARC/LCL 171. Thus, it seems very hard to hypothesize on its application as a biopesticide, although the SAR study performed was useful in order to speculate on the mechanisms of action of these natural products. The functions in nature of these two mycotoxins have not been clearly established, but they are believed to play a role in eliminating other microorganisms competing in the same environment [59].

### 2.2. Bactericides

**Scheme 9:**
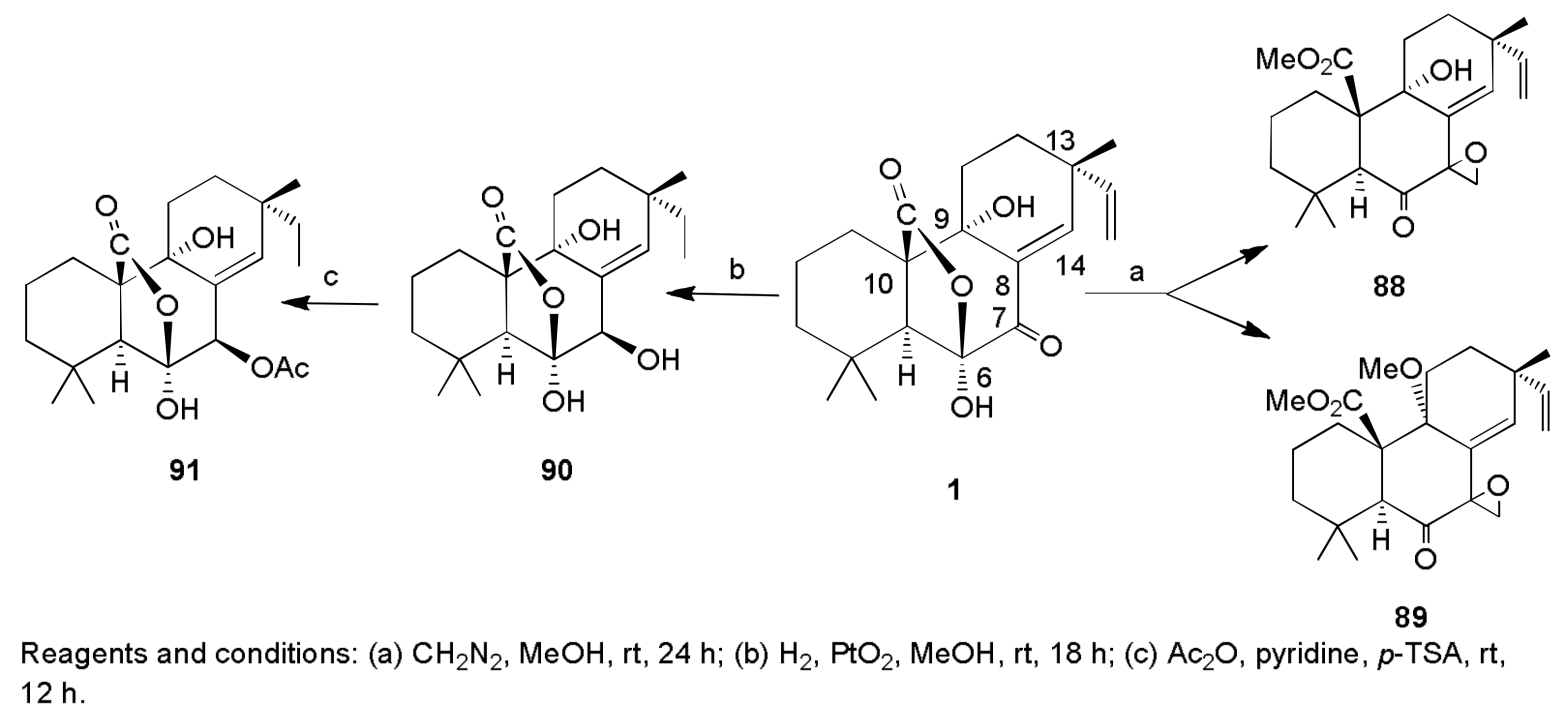
The structures of sphaeropsidin A (**1**) and its derivatives (**88**–**91**).

**Scheme 10:**
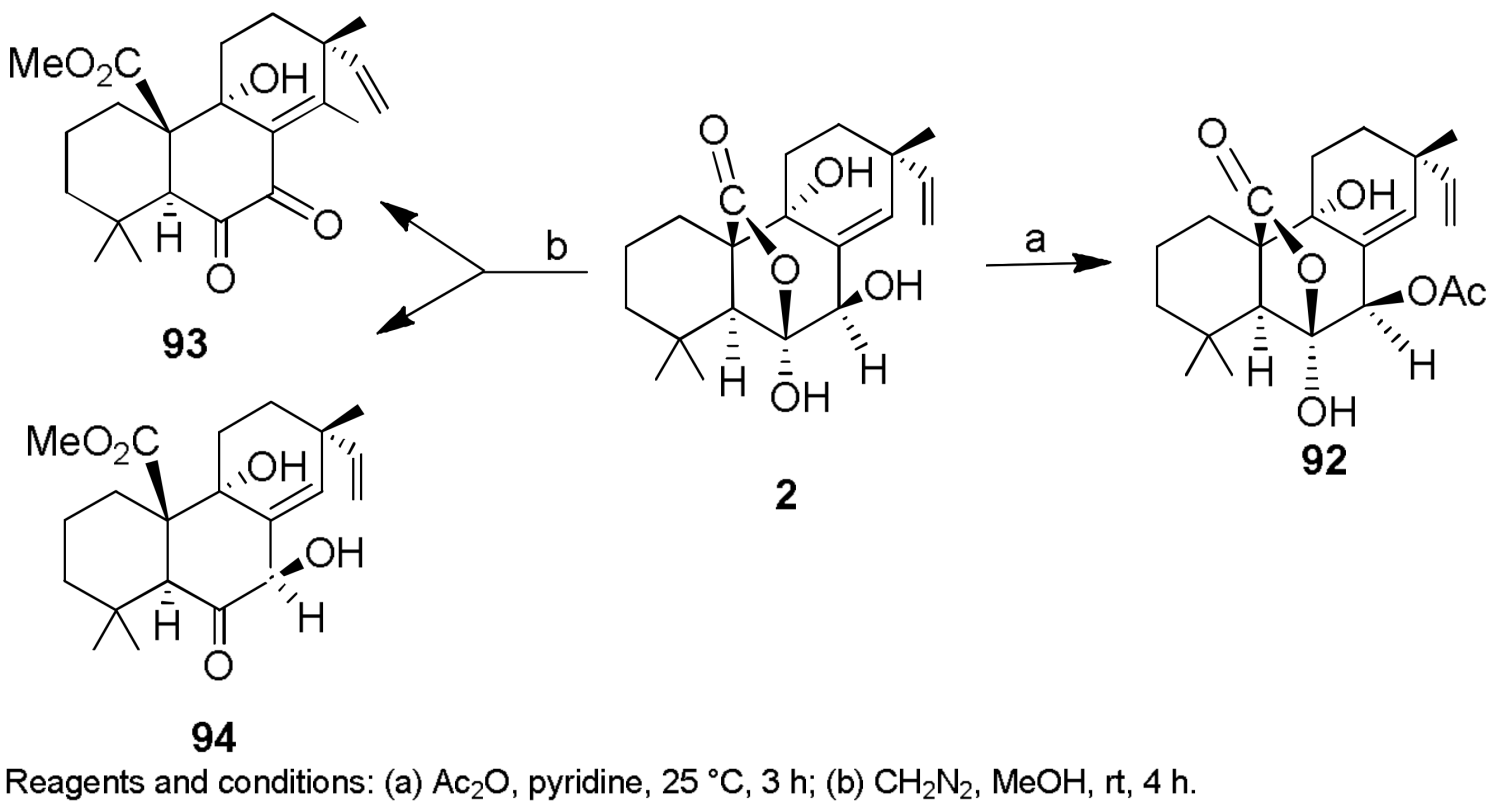
The structures of sphaeropsidin B (**2**) and its derivatives (**92**–**94**).

Several bacterial species are able to cause a large number of different plant diseases, some of which are devastating for agricultural crops [60]. Among the pathogens associated with rice diseases, *Xanthomonas oryzae* pv. *oryzae* is the causal agent of bacterial blight [61,62]. This disease is one of the most serious bacterial diseases in many of the rice-growing regions of the world. Every year, fungal and bacterial diseases cause damage to crops and lead to severe economic losses of rice production. Thus, different studies were carried out to investigate the potential antibacterial activity of fungal metabolites. In particular, sphaeropsidin A (**1**, Figure 1)—the main phytotoxin produced by *D. cupressi*—showed good activity against several bacterial rice pathogens (*X. oryzae* pv. *oryzae*, *Pseudomonas fuscovaginae*, and *Burkholderia glumae*). For this reason, fourteen derivatives were prepared by chemical transformation of the functionalities present in sphaeropsidins A, B, and C (**11**–**14**, Scheme 1; **15** Scheme 2; **16**–**18** Scheme 3; **88**–**91** Scheme 9; **92**–**94**, Scheme 10) and tested (at a concentration range of 0.5−6.0 × 10^−3^ M) against three rice bacterial pathogens to identify which structural features are essential for this biological activity [63].

The results obtained showed a strong and specific activity of sphaeropsidin A against *X. oryzae* pv. *oryzae*, while no activity was observed against the other two pathogens. The results of the SAR study showed that the structural features important to impart this antibacterial activity are the presence of the ketone at C-7 (probably involved in a Michael reaction) and the hemiketal lactone functionality. The C-13 vinyl group, the double bond of ring C, and/or the tertiary hydroxyl group at C-9, as well as the pimarane arrangement of tricylic carbon skeleton, are also important for the antibacterial activity. These findings may be useful in designing novel compounds for practical applications in agriculture as bactericides. However, selectivity studies are necessary to understand if **1** is a suitable compound to develop a new biopesticide formulation. On the other hand, it is important to take into account its other biological activities with particular focus on the concentrations under which sphaeropsidin A turns out to be active [63].

**Figure 7:**
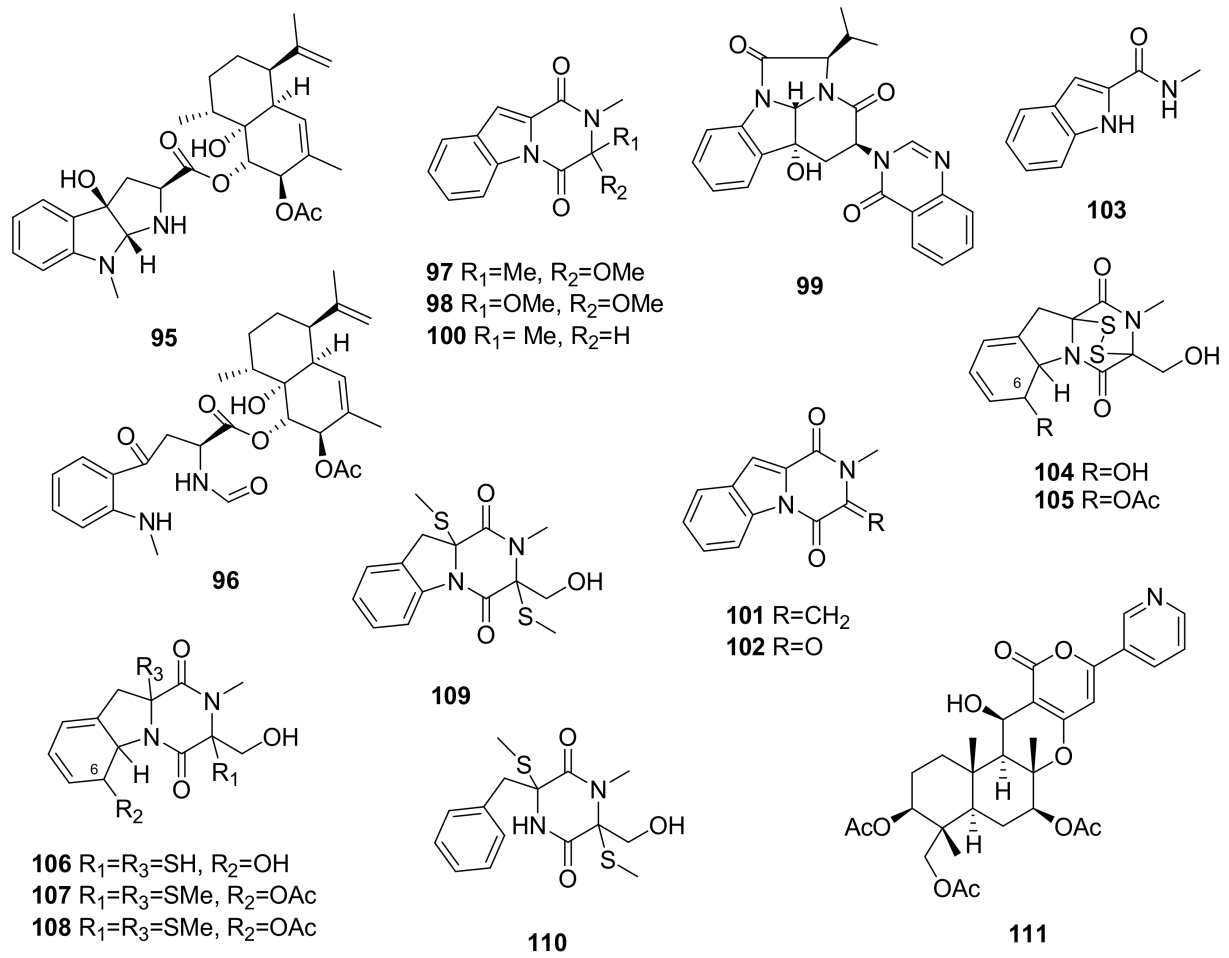
The structures of fischerindoline (**95**), eurochevalierine (**96**), neosartins A–C (**97**–**99**), 1,2,3,4-tetrahydro-2,3-dimethyl-1,4-dioxopyrazino[1–a]indole (**100**), 1,2,3,4-tetrahydro-2-methyl- 3-methylene-1,4-dioxopyrazino[1–a]indole (**101**), 1,2,3,4-tetrahydro-2-methyl-1,3,4-trioxopyrazino [1–a]indole (**102**), *N*-methyl-1*H*-indole-2-carboxamide (**103**), gliotoxin (**104**), acetylgliotoxin (**105**), reduced gliotoxin (**106**), 6-acetylbis(methylthio)gliotoxin (**107**), bisdethiobis(methylthio)gliotoxin (**108**), didehydrobisdethiobis(methylthio)gliotoxin (**109**), bis-*N*-norgliovictin (**110**), and pyripyropene A (**111**).

Some bacteria species are also pathogenic for humans and the bacterial resistance to antimicrobial drugs is an increasing health and economic problem [23,64]. Natural products continue to provide key scaffolds for drug development and new bactericides could be discovered studying the fungal secondary metabolism [26]. Recently, the biosynthetic potential of the marine fungus *Neosartorya pseudofischeri* (isolated from the inner tissue of starfish *Acanthaster planci*) has been evaluated in different culture conditions to find new bactericides [65]. *N. pseudofischeri* was previously studied for the production of secondary bioactive metabolites possessing in vitro anticancer activity, and a new pyrroloindole sesquiterpenoid named fischerindoline (**95**, Figure 7) was isolated together with the promising compound eurochevalierine (**96**, Figure 7) [66,67].

Three new metabolites named neosartins A–C (**97**–**99**, Figure 7) were isolated together with twelve other already-known compounds (**100**–**111**, Figure 7) from in vitro cultures of *N. pseudofischeri* [65].

Compounds **98**–**110** were evaluated for their antibacterial activity against three multidrug-resistant bacteria, that is, the Gram-positive *Staphylococcus aureus* and *Methicillin-resistant Staphylococcus aureus* and the Gram-negative *Escherichia coli*. Compounds **104** and **106** displayed significant inhibitory activities against these three bacteria with MIC values ranging from 1.52 to 97.56 μM. Compounds **104** and **106**, especially, showed potent inhibition against Methicillin-resistant *S. aureus* with MIC values of 1.53 and 1.52 μM, respectively. Compounds **101** and **105** inhibited the growth of both *S. aureus* strains with MIC values of 283.11, 70.70 μM and 86.91, 21.73 μM, respectively. The remaining nine compounds were inactive in this assay (MIC > 256 μg/mL). Compounds **104** and **106** only inhibited the growth of *S. aureus*. Considering the results obtained, a preliminary analysis of the structure-activity relationships of these twelve diketopiperazines suggests that the disulfide bridge, the α-methylene ketone group, the hydroxyl group at C-6, and the thiol groups are important to impart the activity. In particular, the presence of the disulfide bridge or the reduced disulfide bond are essential for the inhibitory activity because when the thiol groups are substituted, the inhibitory effects disappeared. Furthermore, the intensity of the antibacterial activity is influenced by the substitution at the six-membered ring containing two conjugated double bonds and the analogs with a hydroxyl group at C-6 enhance the antibacterial activity compared to the analogs with an acetyl group at the same position [52].

**Figure 8:**
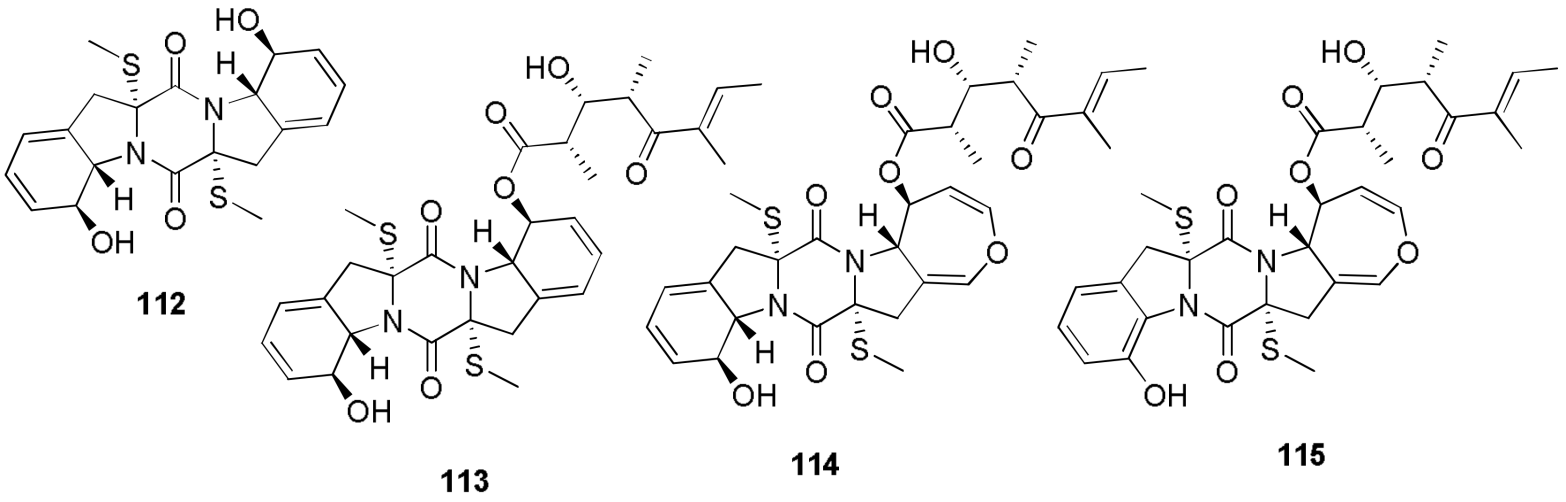
The structures of boydines A–D (**112**–**115**).

From the culture filtrates of the fungus *Pseudallescheria boydii* residing in the gut of a coleopteran (*Holotrichia parallela*) larva, four new epipolythiodioxopiperazines named boydines A–D (**112**–**115**, Figure 8) were isolated together with six other known and new metabolites [68]. All the isolated fungal metabolites were assayed for in vitro antibacterial activity against pathogenic anaerobes isolated from clinical specimens. Only boydine B (**113**) showed a strong inhibition against *Peptostreptococcus* sp., *Bifidobacterium* sp., *Anaerostreptococcus* sp., and *Veillonella parvula* with a MIC range of 0.2–0.8 μM. The SAR study on the cyclic dipeptides suggested that the antibacterial activity might be dependent on the combination of the acyl residue with a diketopiperazine nucleus condensed from two (2*R*,7*S*,7a*S*)-7-hydroxy-2-(methylthio)-2,3,7,7a-tetrahydro-1*H*indole-2-carboxylic acids [68].

The above reported natural and hemisynthetic cytochalasins (**43**–**52**) were also tested against two bacteria: *Pseudomonas syringae* (gram-negative) and *Bacillius megaterium* (gram-positive) [51]. Only cytochalasin A (**43**) and its 17-*O*-acetyl derivative (**47**) were active against *B. megaterium*, while no effects were observed on *P. syringae*. This may be attributed to the different cell walls of these two classes of bacteria which caused a different permeability of the compounds assayed. Furthermore, the activity of **47** could be explained by considering the fact that the acetylation of **43** increased the lypophilicity of the compound. However, in this case, the results obtained correlating the structures and the activity of compounds **42**–**51** highlight the importance of the ketone group at C-20 in **44**. The same compounds (**43**–**52**) were also tested in the brine shrimp bioassay to evaluate their toxicity at concentrations of 20, 2, and 0.2 μg/mL. Cytochalasin E (**50**) was the most active mycotoxin, but generally, at low concentrations, the cytochalasans with the (11)-macrocyclic ring are more active than those with the (14)-macrocyclic ring [51]. Considering that cytochalasins are primarily known to act against eukaryotes as they strongly inhibit actin polymerization, the activity described in this study against bacteria may be an off target effect. 

**Figure 9:**
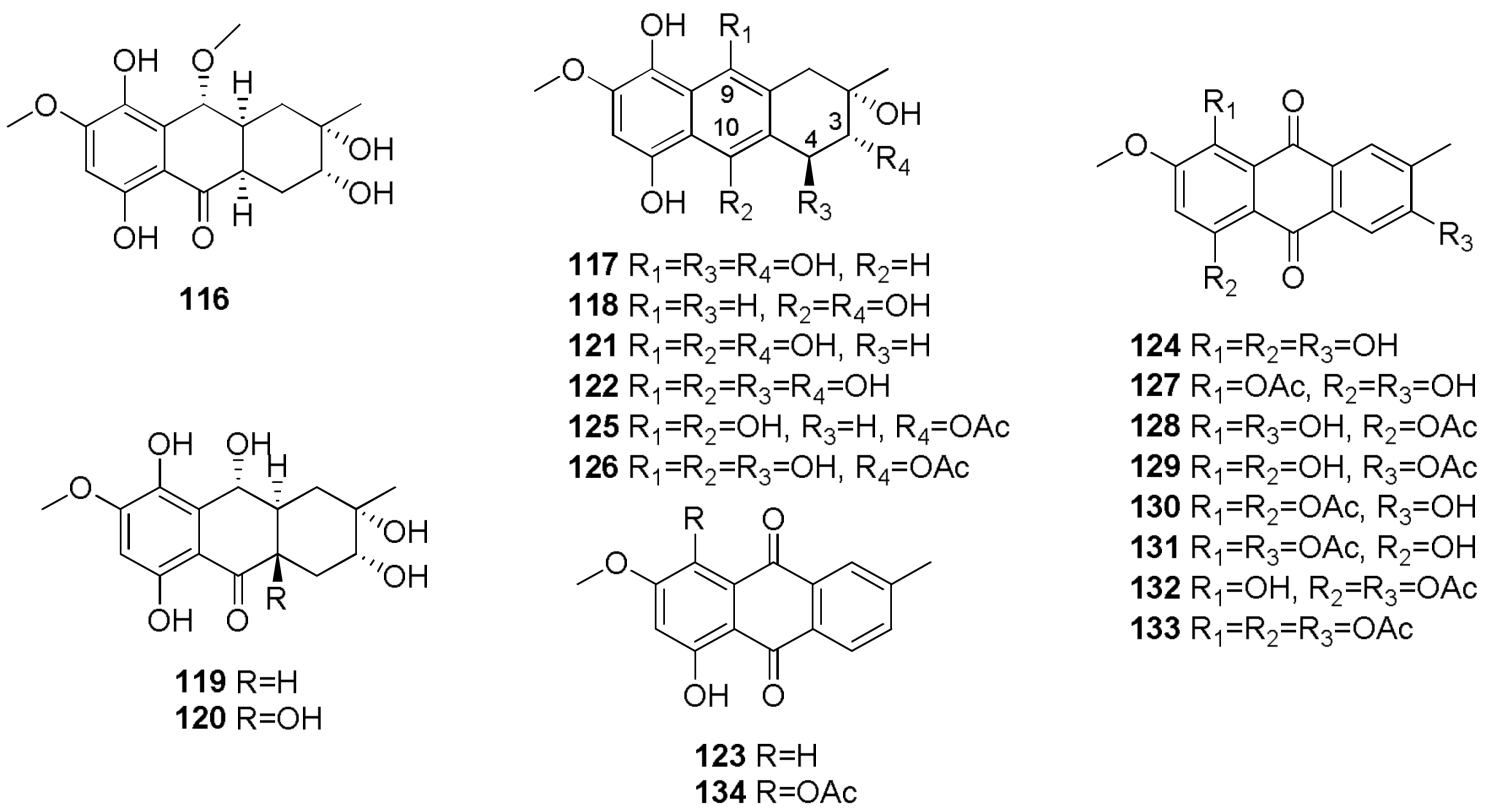
The structures of 4a-*epi*-9α-methoxydihydrodeoxybostrycin (**116**), 10-deoxybostrycin (**117**), nigrosporin B (**118**), 9α-hydroxydihydrodesoxybostrycin (**119**), 9α-hydroxyhalorosellinia A (**120**), 4-deoxybostrycin (**121**), bostrycin (**122**), austrocortirubin (**123**), 3,5,8-trihydroxy-7-methoxy-2-methylanthracene-9,10-dione (**124**), 3-acetoxy-4-deoxybostrycin (**125**), 3-acetoxybostrycin (**126**), 8-acetoxy-3,5-dihydroxy-7-methoxy-2-methylanthracene-9,10-dione (**127**), 5-acetoxy-3,8-dihydroxy-7-methoxy-2-methylanthracene-9,10-dione (**128**), 3-acetoxy-5,8-dihydroxy-7-methoxy-2-methylanthracene-9,10-dione (**129**), 5,8-diacetoxy-3-hydroxy-7-methoxy-2-methylanthracene-9,10-dione (**130**), 3,8-diacetoxy-5-hydroxy-7-methoxy-2-methylanthracene-9,10-dione (**131**), 3,5-diacetoxy-8-hydroxy-7-methoxy-2-methylanthracene-9,10-dione (**132**), 3,5,8-triacetoxy-7-methoxy-2-methylanthracene-9,10-dione (**133**), and 8-acetoxyaustrocortirubin (**134**).

**Figure 10:**
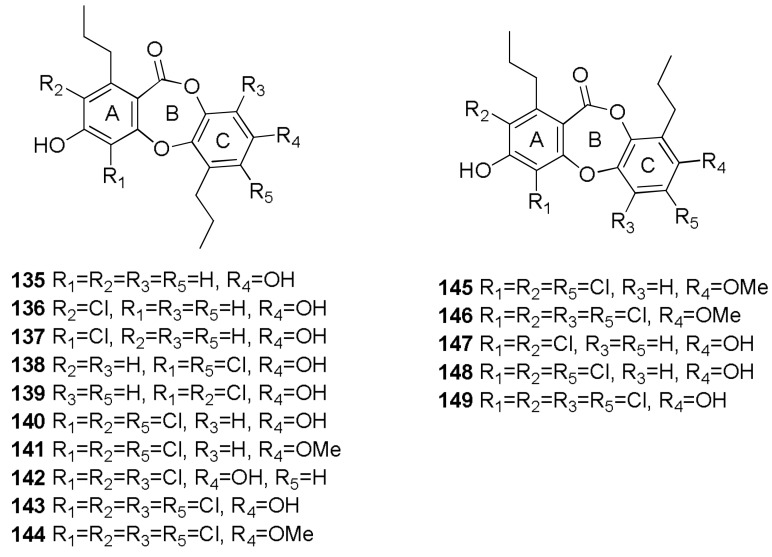
The structures of spiromastixones A–O (**135**−**149**).

From the culture filtrates of the marine-derived fungus ZJ-2010006, isolated from a sea anemone and identified as a *Nigrospora* sp., two new hydroanthraquinone analogs, 4a-*epi*-9α-methoxydihydrodeoxybostrycin (**126**, Figure 9) and 10-deoxybostrycin (**127**, Figure 9), were obtained together with seven known anthraquinone derivatives (**118**−**124**, Figure 9). The antibacterial activity of these compounds was evaluated in vitro together with those of the ten acetyl derivatives (**125**–**134**, Figure 9) prepared from the natural analogs and their structure–activity relationships were examined [69].

In particular, the antibacterial activities were tested against nine bacterial strains including the Gram-positive *Bacillus cereus*, *Bacillus subtilis*, *Staphylococcus albus*, *S. aureus*, *Micrococcus luteus*, and *Micrococcus tetragenus* and the Gram negative *E. coli*, *Vibrio anguillarum*, and *Vibrio parahemolyticus*. Nigrosporin B (**118**) showed strong antibacterial activity against *B. subtilis* (MIC = 312 nM) and *B. cereus* (MIC = 312 nM) while the acetylated derivative **125** exhibited strong activity against *B. cereus* and *V. anguillarum* with MIC values of 48.8 and 97.5 nM, respectively. The SAR study suggested that the cycloaliphatic ring C and the aromatic ring B are very important for the antibacterial activity while the presence of the hydroxyl groups at C-4, C-9, and C-10 are not essential to impart the same activity. Furthermore, the presence of an acetyl group at C-3 in **125** has a positive impact on the antibacterial activity [69].

From the fermentation broth of a deep-sea-derived fungus (*Spiromastix* sp.), 15 new depsidone analogs were isolated by Niu et al. in 2014 and named spiromastixones A–O (**135**–**149**, Figure 10) [70]. They were classified into two subtypes based on the orientation of the C ring relative to the A ring and most analogs were substituted by various numbers of chlorine atoms [70].

Antimicrobial activities were measured against four bacterial strains, namely *E. coli*, *S. aureus*, *Bacillus thuringensis*, and *B. subtilis*, against a spectrum of multiresistant Gram-positive and Gram-negative bacteria species, including reference strains and clinical isolates (methicillin-sensitive and -resistant staphylococci *S. aureus* and *Staphylococcus epidermis*, vancomycin-sensitive and -resistant enterococci *Enterococcus faecalis* and *Enterococcus faecium*) and a number of Gram-negative bacteria containing an extended spectrum β-lactamase (*E. coli*, *K. pneumoniae*). All compounds exhibited significant inhibition against Gram-positive bacteria (MIC values ranging from 0.125 to 8.0 μg/mL) while none of the tested spiromastixones had any significant growth inhibitory effect against the Gram-negative bacterium *E. coli*. Furthermore, compounds **135**–**149** displayed potent inhibitory effects (IC_50_ values ranging from 1 to 32 μM) against methicillin-resistant bacterial strains of *S. aureus* and *S. epidermidis*. Compound **144** also inhibited the growth (IC_50_ values = 4 μM) of the vancomycin-resistant bacteria *E. faecalis* and *E. faecium*. These results allowed for the speculation on the SARs which revealed that the inhibitory effects of the spiromastixones depend on the number of chlorine atoms and the substitution at ring C. In fact, increasing the number of chlorine atoms in the spiromastixones enhanced the inhibitory effects while the compounds with a methoxy substituent at ring C (**141**, **144**–**146**) were more active against the Gram-positive bacteria compared to the analogs that had a hydroxy group at this position (**140**, **143**, **148** and **149**). The selective inhibition against Gram-positive bacteria suggested that the spiromastixones could be promising lead compounds and the specific activity of **144** suggested that further investigation into its potential as an agent to treat multidrug-resistant bacterial infections is required [70]. The results of this SAR study were also confirmed when spiromastixones A–O (**135**–**149**) were tested to evaluate their lipid-lowering activity. In particular, compounds **135**–**149** inhibited foam cell formation via the regulation of cholesterol efflux and uptake in the RAW264.7 macrophages. In this study, the mechanistic investigation revealed that compounds **140** and **148** promoted cholesterol efflux through the upregulation of the PPARγ-ABCA1/G1 pathway and inhibited cholesterol uptake via the downregulation of the scavenger receptors CD36 and SR-A1. Thus, spiromastixones **140** and **148** were considered promising leads for the development of a new type of anti-atherosclerotic agent. However, further studies need to be carried out to investigate their specificity and toxicity [71].

### 2.3. Insecticides

Insecticides of chemical and biological origins constitute a large number of chemical classes which exert toxicity towards insects through different mechanisms of action. They are used in agriculture, forestry, horticulture, gardens, and homes, but also to control vectors such as mosquitoes and ticks [72]. Several insecticidal and nematicidal metabolites were isolated from fungi but very few examples of SAR studies are reported in the literature for these compounds [73]. Most of the work has been done trying to find natural compounds that could have insecticidal activity against *Aedes aegypti,* the major vector of the Zika virus and the viruses responsible for dengue and yellow fevers [1]. These devastating human diseases are some of the major concerns for public health safety. During the last few years, different secondary metabolites produced by plants, fungi, bacteria, and lichens have been studied for their activity against *Ae. aegypti* [74,75,76,77]. 

**Scheme 11:**
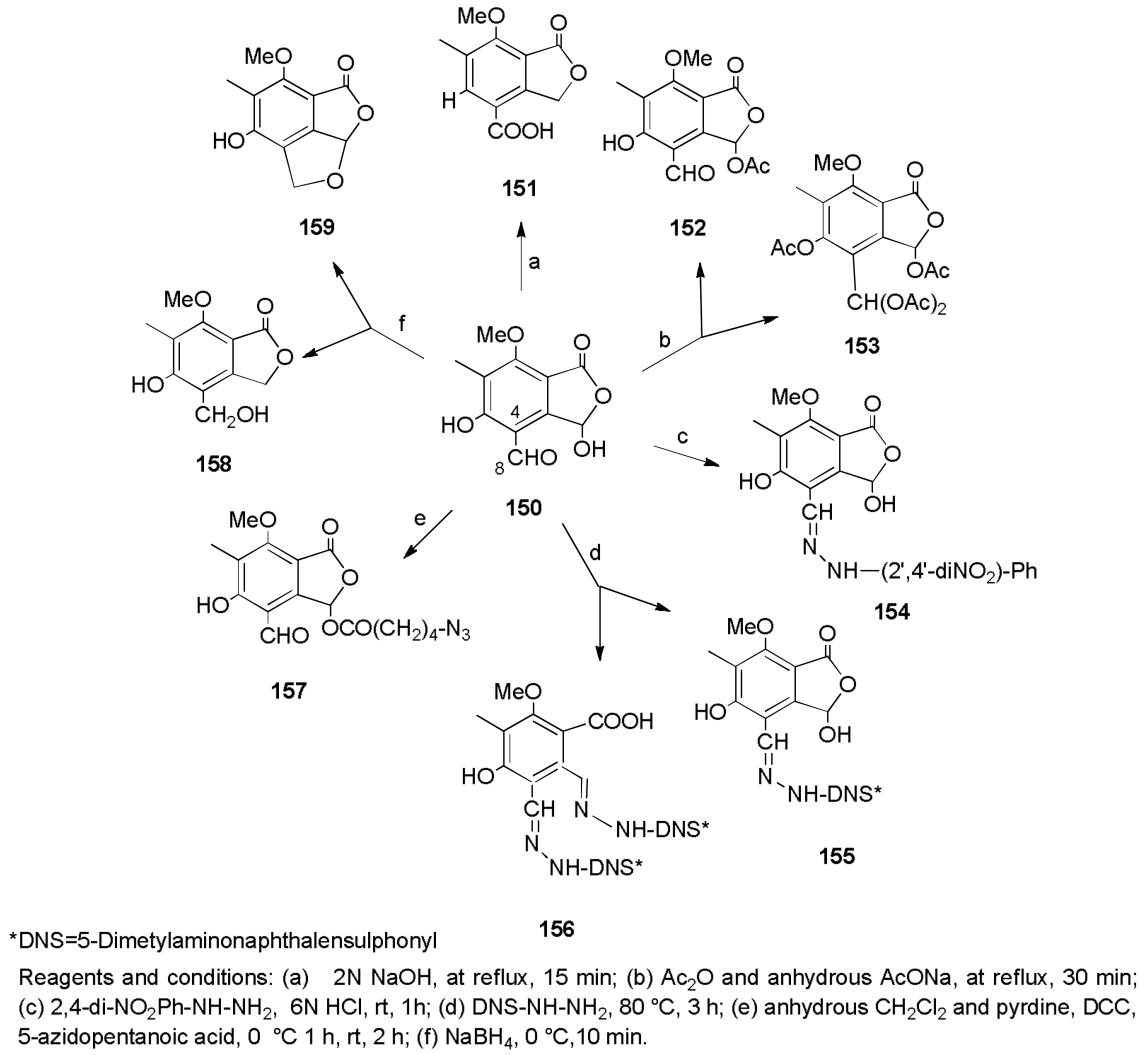
The structures of cyclopaldic acid (**150**) and its derivatives (**151**–**159**).

In a preliminary screening, some fungal phytotoxins were evaluated for their biting deterrent and larvicidal activities against *Ae. aegypti* and SAR studies were conducted with the active compounds preparing some known and new suitable derivatives [78,79]. In particular, starting from cyclopaldic acid (**150**, Scheme 11), produced by the fungus *S. cupressi* involved in the canker disease of Italian cypress (*C. sempervirens*), nine derivatives (**151**–**159**, Scheme 11) were prepared and their insecticidal activity was tested in comparison with the parent compound (**150**) [78,80]. Compounds **155**–**159** were active in biting-deterrence bioassays, confirming the importance of the CHO group at C-4 to impart the deterring activity. These data are in agreement with the results of a previous SAR study in which the phytotoxic activity of **150** and some derivatives was tested on host and non-host plants [81]. However, the lack of activity of **159** indicates that the primary OH group at C-8 should be free and not involved in an ether bond as in the 3,4,5-trisubstituted dihydrofuran ring [78].

**Scheme 12:**
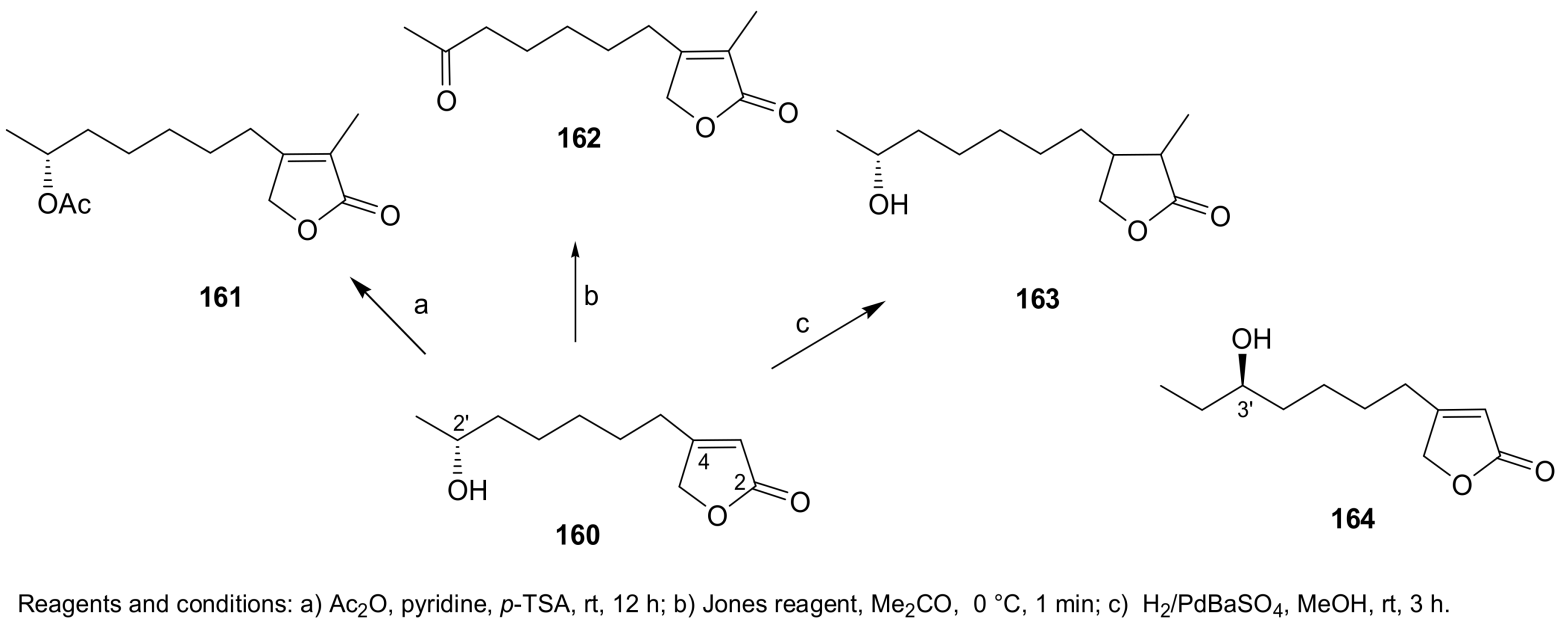
The structures of seiridin (**160**), its derivatives (**161**–**163**), and isoseiridin (**164**).

Additionally, seiridin (**160**, Scheme 12), a metabolite produced by *S. cardinale*—another fungus involved in canker disease of cypress [82]—showed a promising activity against *Ae. aegypti*. Three of its derivatives (**161**–**163**, Scheme 12) were prepared by modifying the main functional groups present in the molecule. These compounds were tested for their biting deterrence and larvicidal activities against *Ae. aegypti* in comparison with **160** and its natural analogue isoseiridin **164** (Scheme 12). The results showed that the presence of an unaltered butenolide ring is very important for the activity while the presence and the position of the OH group of the hydroxyheptyl side chain at C-4 are not important to impart the deterring activity. In addition, the increased activity of 2′-*O*-acetylseiridin (**161**) was probably due to its high lipophilicity (which can facilitate the membrane crossing) and its hydrolysis at the physiological pH (according to the known lethal metabolism) [78].

**Scheme 13:**
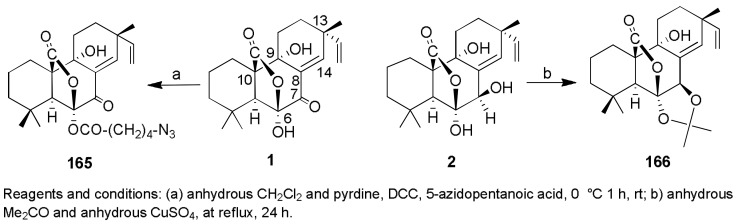
The structures of sphaeropsidins A and B (**1** and **2**) and their derivatives (**165** and **166**).

Additionally, the previously described sphaeropsidin A (**1**, Figure 1) showed interesting activities against *Ae. aegypti* and a SAR study was performed [78]. In particular, some new derivatives (**165** and **166**, Scheme 13) were prepared starting from **1** and **2** and tested together with the known **11** and **14** (Scheme 1) and the natural analogue **2**. The results obtained were in total disagreement with the results of the previous SAR studies [44,63] and indicated that the lactone ring, the hemiketal OH group at C-6, and the C-8=C-14 bond are not relevant for the activity. Furthermore, the increased activity of the sphaeropsidin B (**2**) suggested that the presence of a secondary OH group at C-7 is more significant compared to the C=O group in **1**. Furthermore, this result was not in agreement with the previous SAR studies, indicating a different mode of action for these natural products in imparting biological activities [44,63,78].

**Figure 11:**
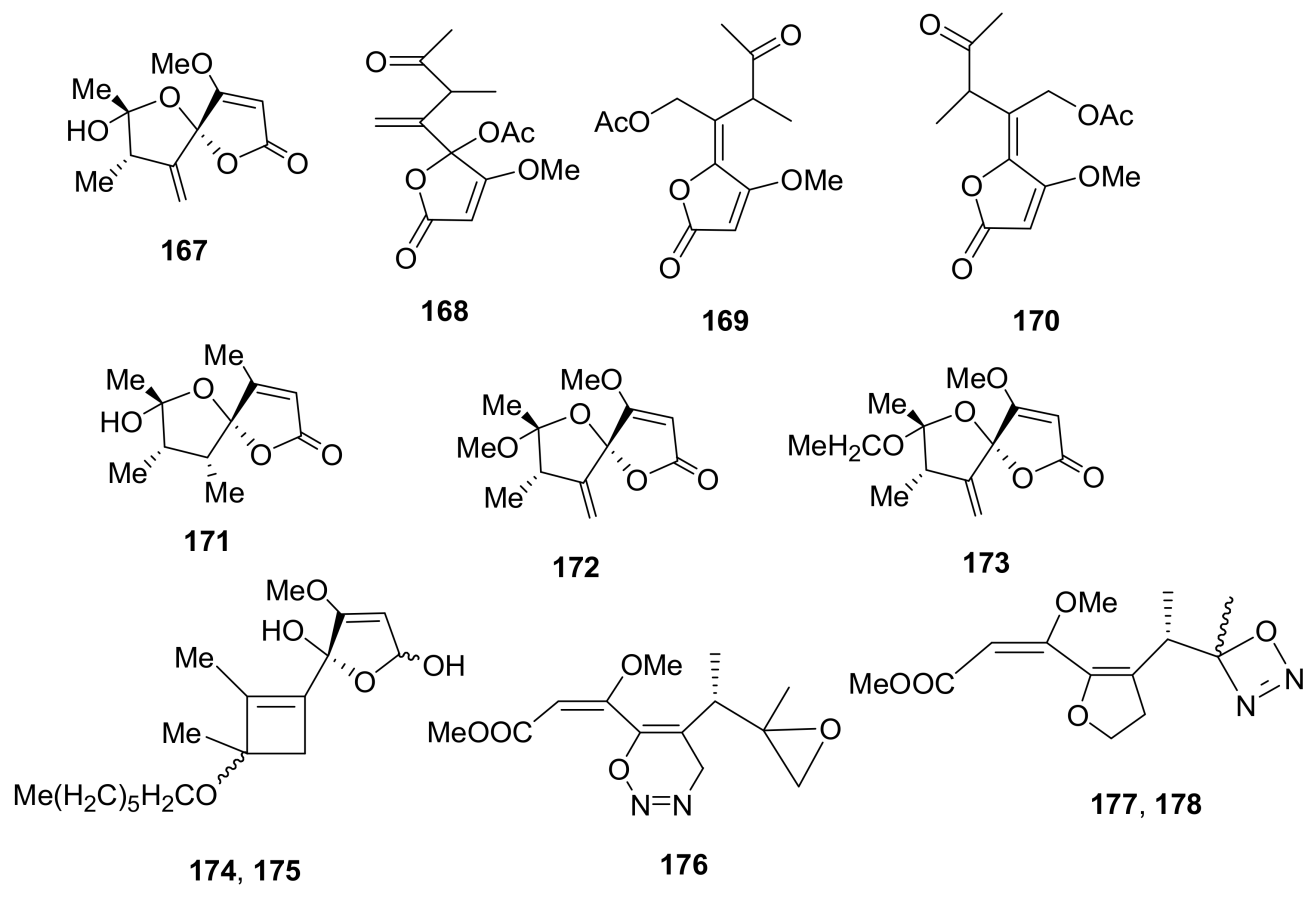
The structures of papyracillic acid (**167**) and some of its derivatives (**168**–**178**).

Because papyracillic acid (**167**, Figure 11) showed a strong mosquito biting deterrent activity, five already known and six new derivatives were prepared to carry out a SAR study (**168**–**178**, Figure 11) [78,79]. Papyracillic acid (**167**) was isolated for the first time in 1996 from the cultures of the ascomycete *Lachnum papyraceum* [83] and then as the main phytotoxin produced by a strain of *Ascochyta agropyrina* var. *nana.* This latter fungus was obtained from the noxious perennial weed *Elytrigia repens* (quack grass) and for this reason, **167** has been previously studied for its potential mycoherbicide activity [84]. In particular, when tested by leaf disk-puncture assay at a concentration of 1 mg/mL, papyracillic acid was shown to be phytotoxic both for the host plant and a number of nonhost plants of the fungus. Papyracillic acid was also active against bacteria (*Xanthomonas campestris* and *B. subtilis*) and the fungus *Candida tropicalis* at 6 μg/disk [84]. When tested against *Ae. aegypti*, all the compounds showed biting deterrence but the activity of compounds **174**–**176** and **178** were similar to the positive control DEET (97%, *N*,*N*-diethyl-3-methylbenzamide). None of these compounds showed any larvicidal activity at the highest testing dose of 100 ppm. These results indicated that the structural feature responsible for the activity of compound **167** is probably the furanone ring. The presence of the alternative substituted cyclobutene, oxiran, or substituted 4*H*-1,2,3-oxadiazine rings in some derivatives may be responsible for an increase in activity [79]. However, **167** is an analogue of penicillic acid, a mycotoxin produced by various fungi including strains of the genera *Penicillium* and *Aspergillus*, and its reactivity was extensively studied. In particular, it was demonstrated that its bioactivities are probably due to its reactivity towards nucleophiles. In fact, when **167** reacted with cysteine and cysteine methyl ester, it exclusively added the thiol group to the exomethylene double bond [85]. Thus, considering the high reactivity of **167** and its low specificity, it is important to further investigate the mode of action and the toxicity of its derivatives before proposing them as an alternative to common insecticides.

**Figure 12:**
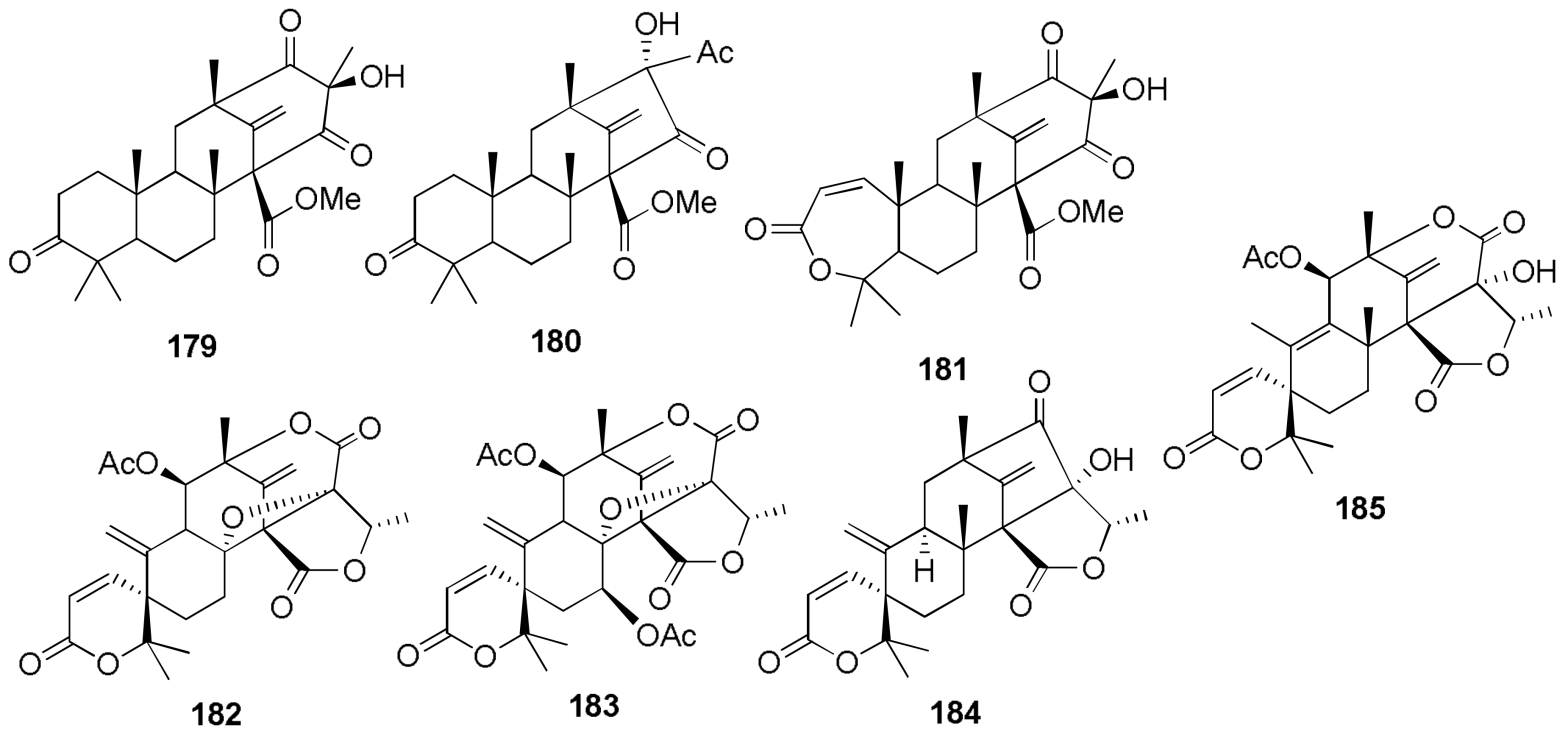
The structures of preaustinoid A (**179**), preaustinoid B (**180**), preaustinoid A2 (**181**), dehydroaustin (**182**), acetoxydehydroaustin (**183**), neoaustin (**184**), and austin (**185**).

Six meroterpenoids (**179**–**185**, Figure 12) were isolated from the solid cultures of a *Penicillium* sp. obtained from *M. azedarach* roots. These compounds were tested (at a concentration of 500 ppm) for their larvicidal effects in the control of *Ae. aegypti*, together with the related meroterpenoid austin (**185**, Figure 12). Compounds **182** and **183** exhibited in vitro larvicidal activities (LC_50_ values of 2.9 and 7.3 ppm, respectively) of 100% and 70%, respectively, after 24 h of exposure while **185** displayed a very low larval mortality. The larvicidal activity displayed by **182** and **183** was probably related to the δ-spirolactone system while the additional AcO group in **183** seems to significantly reduce the larvicidal activity. Furthermore, the very low activity of **185** compared to **182** and **183** suggests that the additional bridging furan ring also significantly influences the activity. This could indicate a hydrophobic binding/reactivity site in this part of the molecule. Since **182** is much more active than natural insecticides, it could have a great potential for the control of *Ae. aegypti* larvae [86]. However, it will first be necessary to more deeply investigate the larvicidal mode-of action and possible effects on non-target organisms before it can be practically used as a natural mosquito-control agent.

**Figure 13:**
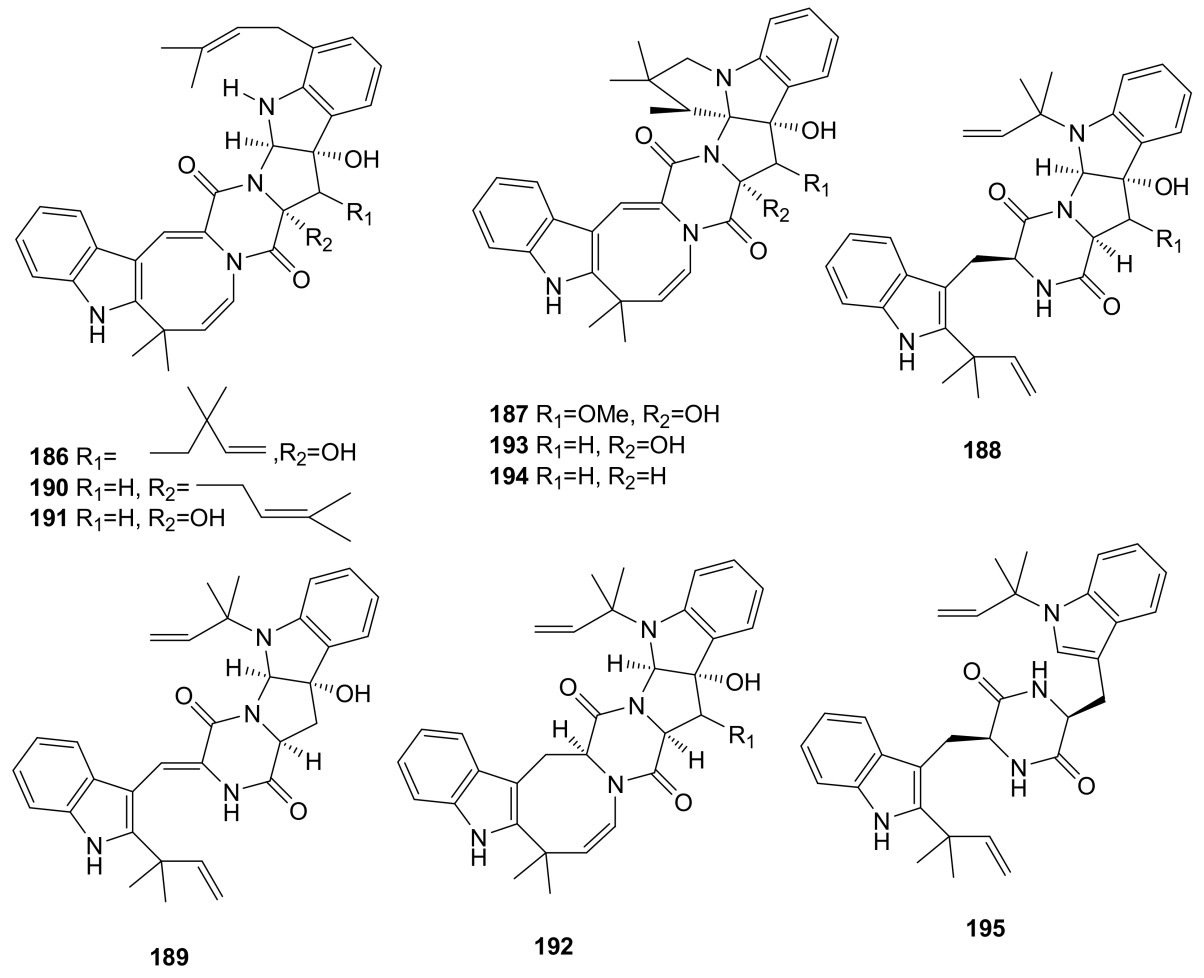
The structures of okaramines A, B, C, G, H, I, N, and Q (**186**–**193**); 2-Dehydroxy-3-demethoxy okaramine B (**194**); and cyclo(*N*^8^-(α,α-dimethylallyl)-*L*-Trp-6a′-(α,α-dimethylallyl)-*L*-Trp) (**195**).

From *Penicillium simplicissimum* AK-40 (ATCC 90288), more than a dozen different prenylated indole alkaloids, named okaramines, were isolated [87,88]. These compounds exhibited insecticidal activity against silkworm larvae of *Bombyx mori* [88]. Okaramine A (**186**, Figure 13) is a heptacyclic compound containing a hexahydropyrrolindole and dihydroazocinoindole while okaramine B (**187**, Figure 13) possesses an unusual octacyclic ring system, including a four-membered azetidine ring and an eight-membered azocine ring [89]. Compound **187** exhibited insecticidal activity against the third instar larvae of silkworm at 0.1 ppm. At a concentration of 0.3 ppm, 100% of the larvae were killed within 24 h. On the other hand, **186** showed much lower activity than **187**, indicating that an azetidine ring and/or a methoxyl in **188** play(s) an important role in expressing the physiological activity. Other SAR studies conducted in order to compare the activities of **187** with those of other natural analogs have suggested that the two characteristic ring structures of **187** play important roles in the insecticidal activity of the compound. A molecular target of **187** was identified as *B. mori* GluCl (BmGluCl) [90]. In order to explore the biosynthetic potential of okaramines, gene knockout experiments of an okaramine-producer fungus were performed. Ten compounds (**186**–**195**, Figure 13) were isolated and tested for BmGluCl-activating activity. Analysis of the structure–activity relationships of **186**–**195** revealed that the 1,4-dihydroazocine and *N*-aliphatic groups attached to the indole are crucial for the GluCl-activating activity. This provided insights into the further derivatization of the complex structure of okaramines in order to facilitate the development of new insecticides [88].

### 2.4. Herbicides

**Figure 14:**
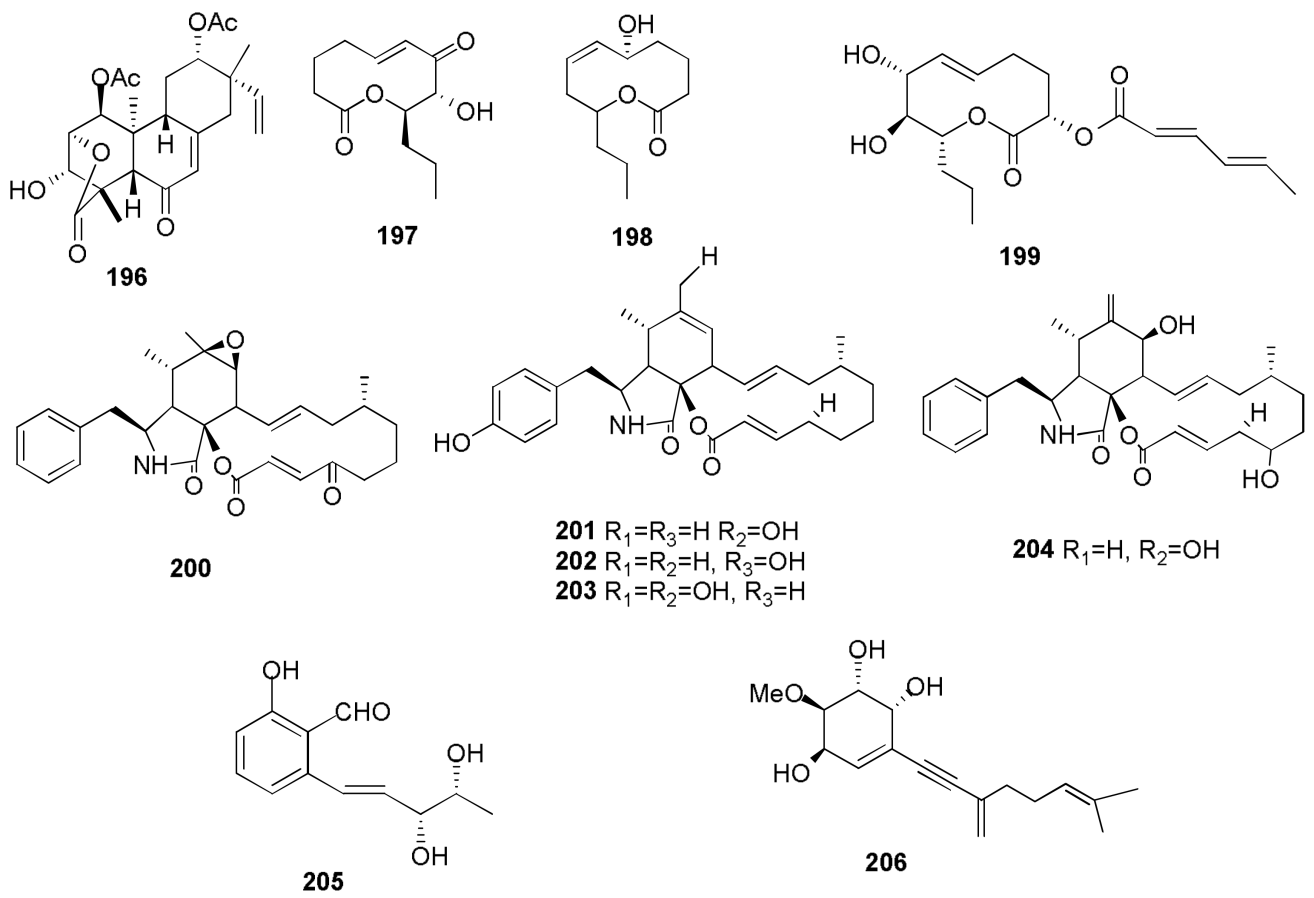
The structures of chenopodolin (**196**); stagonolide (**197**); putaminoxin (**198**); pinolidoxin (**199**); cytochalasins F, T, Z1, Z2, and Z3 (**200**–**204**); agropyrenol (**205**); and phomentrioloxin (**206**).

Weeds (including parasitic, annual, and perennial) are able to compete with a large number of important crops causing severe yield losses worldwide [60]. In order to avoid the use of synthetic chemicals, the phytotoxins produced by weed pathogenic fungi could be an efficient tool to design natural and safe bioherbicides. A recent review has described, in detail, the chemical and biological characterization of fungal phytotoxins with potential herbicidal activity [2]. The same review has also reported the SAR studies conducted on some promising phytotoxins such as chenopodolin (**196**, Figure 14) produced by *Phoma chenopodiicola* for the control of *Cirsium arvense* [91,92]; nonenolides (**197**–**199**, Figure 14) and cytochalasans (**42**–**44**, Figure 3 and **200**–**204**, Figure 14) isolated from phytopathogenic *Stagonospora*, *Phoma*, and *Ascochyta* spp. for the control of *C. arvense* and *Sonchus arvensis* [93]; papyracillic acid (**167**, Figure 11) and agropyrenol (**205**, Figure 14), produced by *A. agropyrina* var. *nana* for the control of *E. repens* [84,94,95]; and phomentrioloxin (**206**, Figure 14), isolated from the liquid culture of *Phomopsis* sp., a fungal pathogen proposed for the biological control of *Carthamus lanatus* [96,97]. The last results which were not covered by the review of Cimmino et al. [2] are reported below.

**Figure 15:**
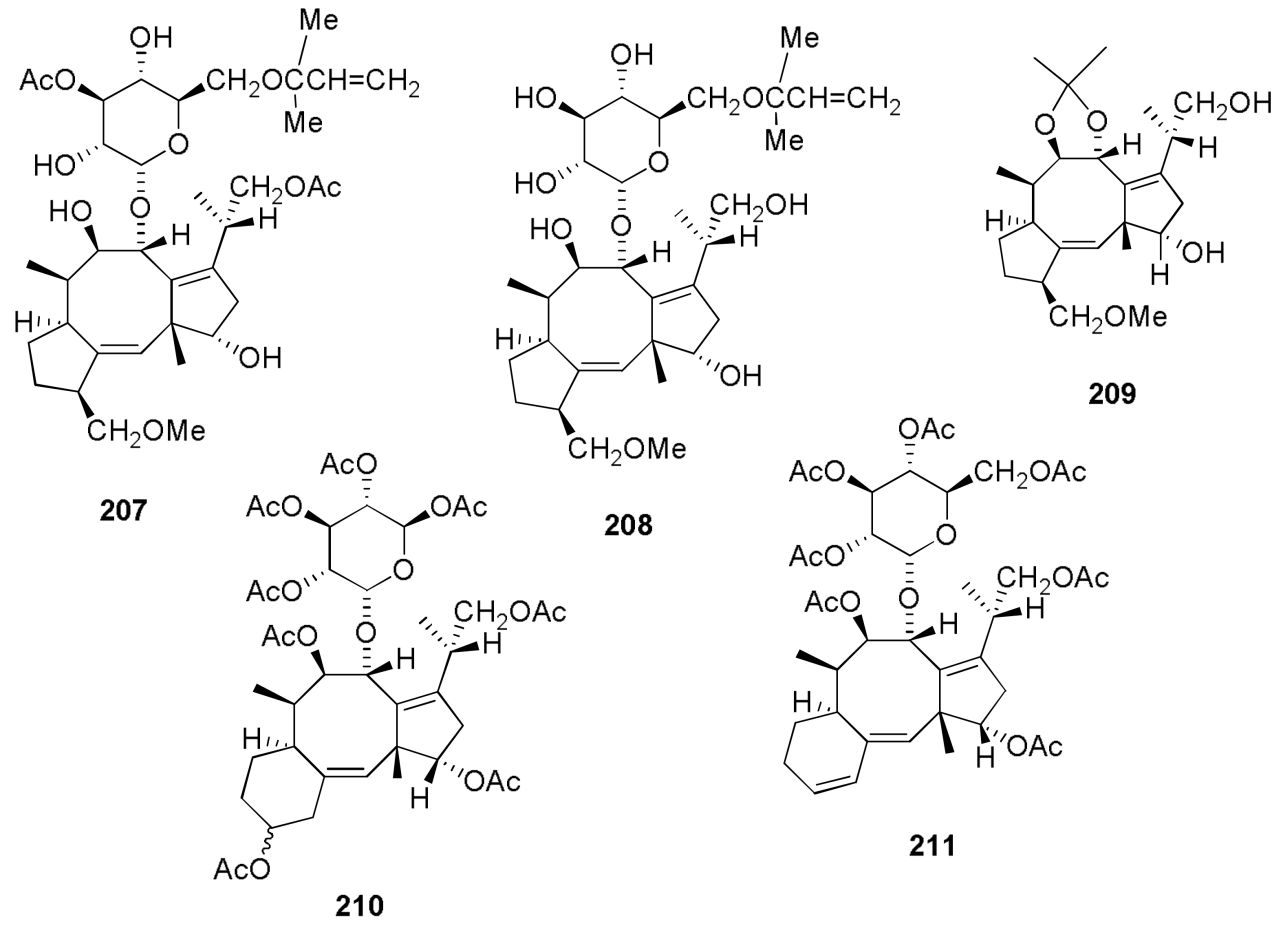
The structures of fusicoccin A (**207**), dideacetylfusicoccin A (**208**), the isopropylidene derivative of fusicoccin aglycone (**209**), and the hexacetyl and pentacetyl isomers of 16-*O-*demethyl-de-*tert*-pentenylfusicoccin A (**210** and **211**).

Among weeds, parasitic plants are of great economic importance causing huge crop losses worldwide and the urgent development of innovative control strategies is due to the lack of effective control methods [98,99,100]. A SAR study was carried out to evaluate the efficacy to stimulate seed germination of the parasitic plant *Orobanche ramosa*. Between the diterpene fusicoccin A (**207**, Figure 15), its aglycone, several fusicoccin A derivatives, and natural analogs were assayed. In this study, the most active compounds appeared to be the dideacetylfusicoccin A and the isopropylidene derivative of fusicoccin aglycone (**208** and **209**, Figure 15) [2,101]. Successively, the effect of fusicoccin A derivatives were evaluated on the seed germination of nine different *Orobanche* species. The results showed that the stimulation of seed germination was species-dependent and also affected by the concentration of the stimulant. Among fusicoccin and its seven derivatives, the highest stimulatory effect was observed for the hexacetyl and pentacetyl isomers of 16-*O-*demethyl-de-*tert*-pentenylfusicoccin A (**210** and **211**, Figure 15) [102].

Recently a SAR study was performed on the above cited sphaeropsidone and *epi*-sphaeropsidone (**7** and **8**, Figure 1) which showed the ability to induce haustorium development in radicals of the parasitic weeds *Striga hermonthica*, *Orobanche crenata*, and *Orobanche cumana* [100].

**Scheme 14:**
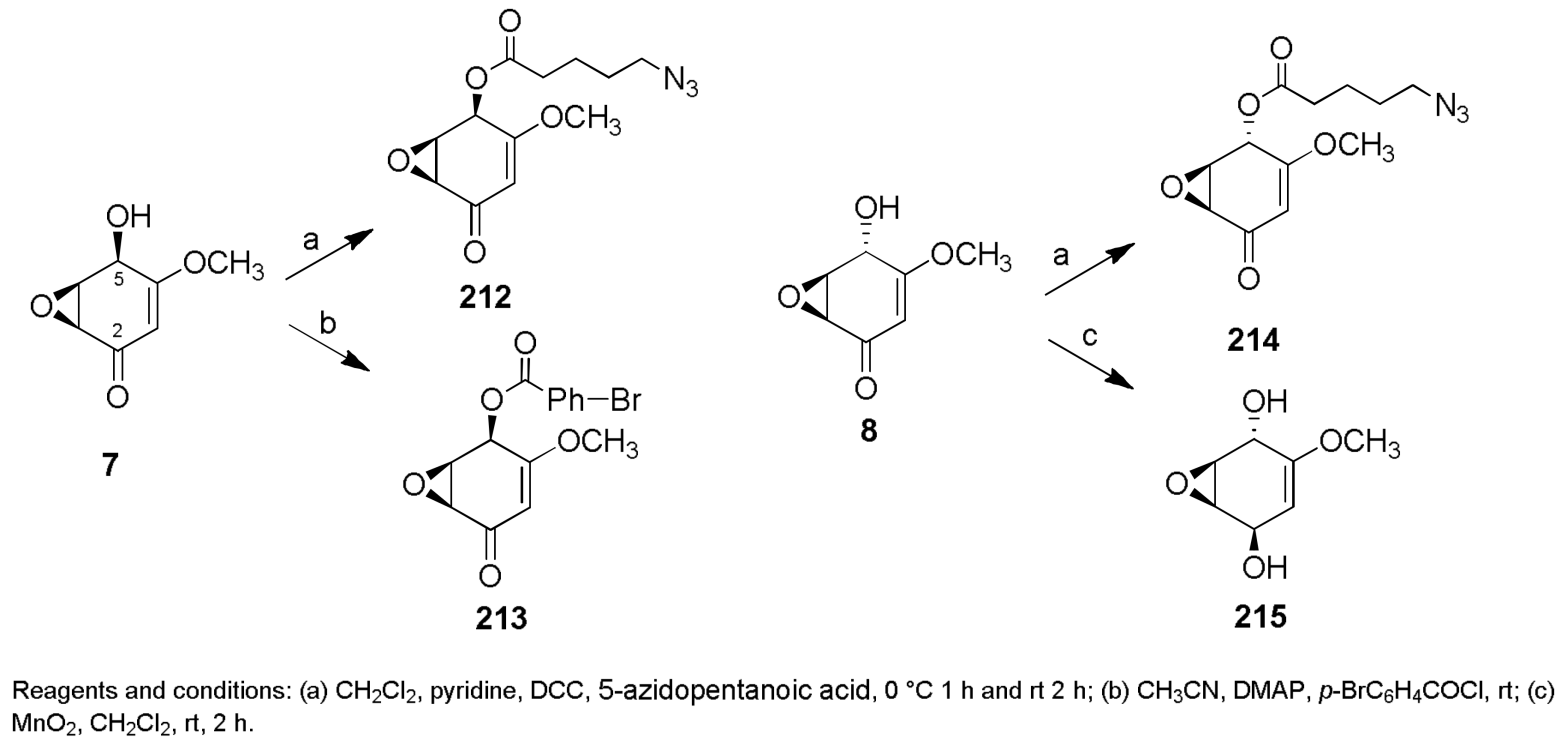
The structures of sphaeropsidone (**7**), its derivatives (**212** and **213**), *epi*-sphaeropsidone (**8**), and its derivatives (**214** and **215**).

The haustorium is a plant organ that the parasitic weeds use to invade the host and to withdraw the plant nutrients. Thus, the stimulation of a haustorium development in radicles of parasitic plants in the absence of the host could be a suitable strategy to manage these weeds. For this reason, seven already known (**19**–**25**, Scheme 4 and Scheme 5) and four new hemisynthetic derivatives (**212**–**215**, Scheme 14) were prepared starting from **7** and **8.** SAR studies were carried out by testing (at different decreasing concentrations between 100 and 0.01 μM) their haustorium-inducing activity in comparison with that of the natural cyclohexene oxides. The results suggested that the haustorium-inducing activity is due to the possibility to convert the natural sphaeropsidone and natural and hemisynthetic derivatives in the corresponding 3-methoxyquinone and that the stereochemistry at C-5 also seems to affect this activity [100].

**Figure 16:**
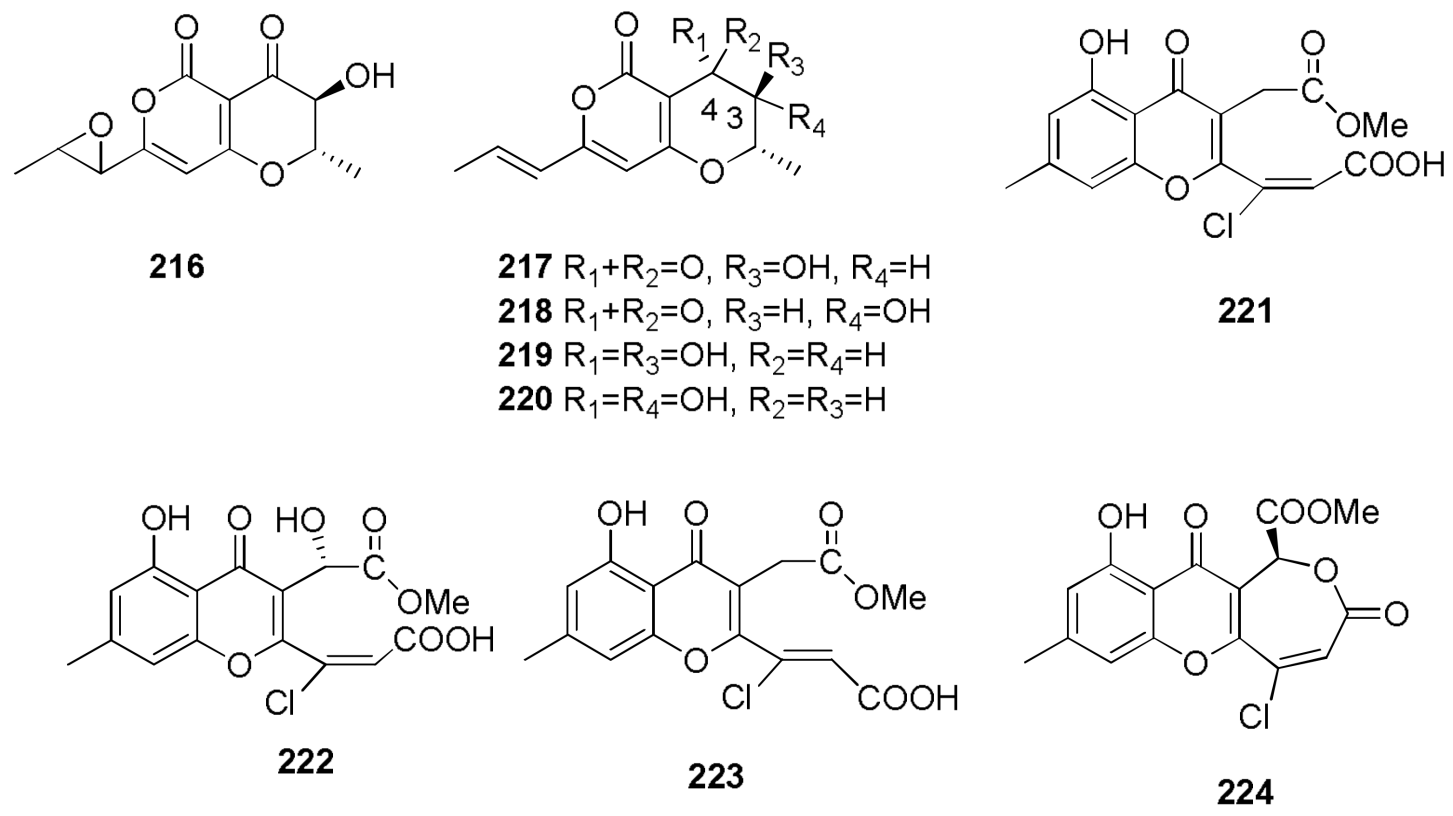
The structures of cochliotoxin (**216**), radicinin (**217**), 3-*epi*-radicinin (**218**), radicinol (**219**), 3-*epi*-radicinol (**220**), chloromonilinic acids B–D (**221**–**223**), and chloromonilicin (**224**).

Buffelgrass (*Pennisetum ciliare*, syn. *Cenchrus ciliare*) is a perennial forage grass introduced from Africa that is widely planted for livestock forage in Texas and northern Mexico. However, buffelgrass has become a very serious invader in the Sonoran Desert of southern Arizona where it increases the frequency of fire and has the potential to destroy the iconic saguaro woodland ecosystem. Considering that buffelgrass is difficult to eradicate with chemical herbicides without major damage to native vegetation, two primary foliar pathogens in its introduced range, namely *Cochliobolus australiensis* and *Pyricularia grisea*, were studied to evaluate their ability to produce phytotoxic secondary metabolites which could be used as potential natural herbicides [103,104,105]. In particular, from the liquid culture of *C. australiensis*, a new phytotoxin named cochliotoxin (**216**, Figure 16) was isolated together with radicinin, radicinol, and their 3-epimers (**217**–**220**, Figure 16). The fungus produced these compounds in two different liquid media together with two new tetrasubstituted 3-chromanonacrylic acids named chloromonilinic acids C and D, and the known chloromonilinic acid B and chloromonilicin (**221**–**224**, Figure 16). However, the radicinin-related compounds were also produced when the fungus was grown in wheat seed solid culture, but chloromonilinic acids were not found in the solid culture organic extract. These results were not surprising because it is known that some fungi are able to produce metabolites belonging to disparate classes of natural compounds when grown in different culture conditions as recently demonstrated for the seed pathogen *Pyrenophora semeniperda*. In fact, this fungus showed the ability to produce cytochalasins and pyrenophoric acids when grown on cheatgrass and wheat seed cultures but spirostaphylotrichins when grown in liquid cultures (PDB) [48,49,106,107,108].

Cochliotoxin was characterized by spectroscopic methods as 3-hydroxy-2-methyl-7-(3-methyloxiranyl)-2,3-dihydropyrano[4,3-b]pyran-4,5-dione. Its relative stereochemistry was assigned by ^1^H NMR techniques, while the absolute configuration (2*S*,3*S*) was determined by applying the advanced Mosher’s method by esterification of its hydroxy group at C-3 [103,109].

When bioassayed in a buffelgrass coleoptile elongation test and by leaf puncture bioassay against the host weed and two nontarget kinds of grass, cochliotoxin, radicinin, and 3-*epi*-radicinin showed phytotoxicity while radicinol and 3-*epi*-radicinol were largely inactive. On the basis of these results, some preliminary structure–activity relationships between **216** and its analogs (**217**–**220**) were considered. In particular, the presence at C-4 of the α,β-unsaturated ketone in **216**–**218** seems to play a central role in the strong phytotoxic activities of these compounds. In fact, the absence of this moiety in **219** and **220** causes a noticeable activity reduction at the higher concentration used on buffelgrass and the complete inactivity in the leaf puncture assay at lower concentrations on the native grasses. Furthermore, the stereochemistry of the chiral C-3 in **216**–**218** as well as the presence of the epoxy group in **216** seem also to be important features involved in modulating the activity of these compounds [103].

## 3. Conclusions

Natural products have been used since ancient times in different fields due to their abundant scaffold diversity and the several biological activities that they show. These compounds have also had a substantial impact on pest control and drug discovery, identifying novel modes of action and serving as inspiration/models for synthetic compounds. Among the living organisms, fungi represent a very good source of natural substances that could be used as an efficient tool to design natural biopesticides and drugs against human pathogens. Unfortunately, their application into practice as commercial products is still very limited due to the lack of funding to this research field and for the severe regulations adopted by different countries. However, to better understand the mechanisms and modes of action of selected active metabolites, SAR studies are necessary to identify the structural features essential for their biological activities. Furthermore, these studies could help find a derivative of the natural bioactive compound with an increased activity, selectivity, and the stability associated to reduce toxicity, all important properties for potential practical applications.

## Supplementary Materials

A table (Table S1) with all the fungal metabolites cited in the text, their hemisynthetic derivatives and the relative biological activities is reported in the Supplementary Materials available online.

## Abbreviations

Ac_2_O	Acetic anhydride
at.p.	Atmospheric pressure
CH_2_N_2_	Diazomethane
CH_3_CN	Acetonitrile
DCC	*N*,*N′*-Dicyclohexylcarbodiimide
DMAP	4-Dimethylaminopyridine
EtOAc	Ethyl acetate
Et_2_O	Diethyl ether
MeOH	Methanol
Me_2_CO	Acetone
MIC	Minimum inhibitory concentration
PDB	Potato dextrose broth
*p*-TSA	*p*-Toluenesulfonic acid
rt	Room temperature
THF	Tetrahydrofuran
