# Supplementary material for: Fungal Metabolites Antagonists towards Plant Pests and Human Pathogens: Structure-Activity Relationship Studies
Source: Molecules. 2018 Apr 5;23(4):834. doi: 10.3390/molecules23040834 (PMC6017029; doi:10.3390/molecules23040834)
Supplement: Supplementary file 1 [file molecules-23-00834-s001.pdf]

SUPPLEMENTARY MATERIAL

Fungal metabolites antagonists towards plant pests and human pathogens: structure-activity relationship studies

Marco Masi *, Paola Nocera, Pierluigi Reveglia, Alessio Cimmino and Antonio Evidente

Dipartimento di Scienze Chimiche, Università di Napoli Federico II, Complesso Universitario monte Sant'Angelo, Via Cintia 4, 80126 Napoli, Italy; pierlugi.reveglia@unina.it; paola.nocera@unina.it; alessio.cimmino@unina.it; evidente@unina.it

* Correspondence: marco.masi@unina.it; Tel.: +39-081-253-2126

List of content

Table S1. Fungal metabolites, their hemisynthetic derivatives and biological activities.

Table S1. Fungal metabolites, their hemisynthetic derivatives and biological activities.

Name and Number	Class of Natural Compounds	Systematic Name	Source	Activity	Ref.
Sphaeropsidins A (**1**)	pimarane diterpene	(2*R*,4a*R*,4b*R*,8a*S*,9*S*)-4a,9-dihydroxy-2,8,8-trimethyl-2-vinyl-4,4a,5,6,7,8,8a,9-octahydro-2*H*-9,4b-(epoxymethano)phenanthrene-10,12(3*H*)-dione	*Diplodia* spp.	antimycotic; anticancer; antibacterial; insecticidal	[38,39,41-44, 63,78]
Sphaeropsidins B (**2**)	pimarane diterpene	(2*R*,4a*R*,4b*R*,8a*S*,9*S*,10*R*)-4a,9,10-trihydroxy-2,8,8-trimethyl-2-vinyl-3,4,4a,5,6,7,8,8a,9,10-decahydro-2*H*-9,4b-(epoxymethano)phenanthren-12-one	*Diplodia cupressi*	antimycotic; phytotoxic; insecticidal	[44,78]
Sphaeropsidins C (**3**)	pimarane diterpene	(4a*R*,4b*R*,7*R*,10a*S)*-4b-hydroxy-1,1,7-trimethyl-9-oxo-7-vinyl-1,2,3,4,4a,4b,5,6,7,9,10,10a-dodecahydrophenanthrene-4a-carboxylic acid	*D. cupressi*	antimycotic; phytotoxic	[44]
Sphaeropsidins D (**4**)	pimarane diterpene	(2*R*,4*R*,4a*R*,4b*R*,8a*S*,9*S*)-4,4a,9-trihydroxy-2,8,8-trimethyl-2-vinyl-4,4a,5,6,7,8,8a,9-octahydro-2H-9,4b-(epoxymethano)phenanthrene-10,12(3H)-dione	*D. cupressi*	Phytotoxic	[44]
Sphaeropsidins E (**5**)	pimarane diterpene	(4*R*,4a*R*,4b*R*,7*R*,9*S*,10*R*,10a*S*)-1,1,4a,7-tetramethyl-7-vinyl-1,2,3,4,4a,4b,5,6,7,9,10,10a-dodecahydrophenanthrene-4,4b,9,10-tetraol	*D. cupressi*		[44]
Sphaeropsidins F (**6**)	pimarane diterpene	(1*S*,2*R*,4*S*,4b*S*,8a*S*,10*R*)-1,2,4b,8,8-pentamethyl-2-vinyl-1,2,3,4,4b,5,6,7,8,8a,9,10-dodecahydrophenanthrene-4,10-diol	*D. cupressi*		[44]
Sphaeropsidone (**7**)	cyclohexanone	(1*S*,5*R*,6*S*)-5-hydroxy-4-methoxy-7-oxabicyclo[4.1.0]hept-3-en-2-one	*D. cupressi*	antioomycetes; phytotoxic	[45,100]
epi-Sphaeropsidone (**8**)	cyclohexanone	(1*S*,5*S*,6*S*)-5-hydroxy-4-methoxy-7-oxabicyclo[4.1.0]hept-3-en-2-one	*D. cupressi*<br>*Diplodia africana*	antioomycetes; phytotoxic	[45,100]
Compound (**9**)	cyclohexanone	(4*S*,6*S*)-6-chloro-4,5-dihydroxy-3-methoxycyclohex-2-enone	*D. cupressi*		[45,100]
Compound (**10**)	cyclohexanone	(4*S*,6*R*)-6-chloro-4,5-dihydroxy-3-methoxycyclohex-2-enone	*D. cupressi*		[45,100]
Compound (**11**)	pimarane diterpene	(2*R*,4a*R*,4b*R*,8a*S*,9*S*)-4a-hydroxy-2,8,8-trimethyl-10,12-dioxo-2-vinyl-3,4,4a,5,6,7,8,8a,9,10-decahydro-2*H*-9,4b-(epoxymethano)phenanthren-9-yl acetate	hemisynthesized from **1** for SAR studies	phytotoxic; antimycotic	[44,63,78]
Compound (**12**)	pimarane diterpene	(2*R*,4b*R*,8a*S*,9*S*)-2,8,8-trimethyl-10,12-dioxo-2-vinyl-2,3,4,5,6,7,8,8a,9,10-decahydro-1*H*-9,4b-(epoxymethano)phenanthrene-1,9-diyl diacetate	hemisynthesized from **1** for SAR studies		[44,63]
Compound (**13**)	pimarane diterpene	(2*S*,4a*R*,4b*R*,8a*S*,9*S*)-2-ethyl-4a,9-dihydroxy-2,8,8-trimethyl-4,4a,5,6,7,8,8a,9-octahydro-2*H*-9,4b-(epoxymethano)phenanthrene-10,12(3*H*)-dione	hemisynthesized from **1** for SAR studies		[44,63]
Compound (**14**)	pimarane diterpene	(3*R*,3a*S*,4a*R*,6a*S*,10a*R*,10b*R*)-methyl 10b-hydroxy-3,7,7-trimethyl-5,6-dioxo-3-vinyltetradecahydrocyclopropa[j]phenanthrene-10a-	hemisynthesized from **1** for SAR studies		[44,63,78]

		carboxylate			
Compound (**15**)	pimarane diterpene	(1*R*,3a'*S*,4*R*,7a'*R*)-2-formyl-4,4',4'-trimethyl-3'-oxo-4-vinyl-3a',4',5',6',7',7a'-hexahydro-3'*H*-spiro[cyclohex[2]ene-1,1'-isobenzofuran]-7a'-carboxylic acid	hemisynthesized from **2** for SAR studies		[44,63]
Compound (**16**)	pimarane diterpene	(4a*R*,4b*R*,7*R*,9*S*,10a*S*)-4b,9-dihydroxy-1,1,7-trimethyl-7-vinyl-1,2,3,4,4a,4b,5,6,7,9,10,10a-dodecahydrophenanthrene-4a-carboxylic acid	hemisynthesized from **3** for SAR studies		[44,63]
Compound (**17**)	pimarane diterpene	(3*R*,3a*S*,4a*R*,6a*S*,10a*R*,10b*R*)-methyl 10b-hydroxy-3,7,7-trimethyl-5-oxo-3-vinyltetradecahydrocyclopropa[j]phenanthrene-10a-carboxylate	hemisynthesized from **3** for SAR studies		[44,63]
Compound (**18**)	pimarane diterpene	(4a*R*,4b*R*,7*R*,10a*S*)-methyl 4b-hydroxy-1,1,7-trimethyl-9-oxo-7-vinyl-1,2,3,4,4a,4b,5,6,7,9,10,10a-dodecahydrophenanthrene-4a-carboxylate	hemisynthesized from **3** for SAR studies		[44,63]
Compound (**19**)	cyclohexanone	(1*S*,2*R*,6*S*)-3-methoxy-5-oxo-7-oxabicyclo[4.1.0]hept-3-en-2-yl acetate	hemisynthesized from **7** for SAR studies		[45,100]
Compound (**20**)	aromatic compound	5-methoxybenzene-1,2,4-triyl triacetate	hemisynthesized from **7** for SAR studies		[45,100]
Compound (**21**)	cyclohexandione	(1*R*,6*S*)-3-methoxy-7-oxabicyclo[4.1.0]hept-3-ene-2,5-dione	hemisynthesized from **7** for SAR studies	antioomycetes	[45,100]
Compound (**22**)	cyclohexanone	(4*R*,5*S*,6*R*)-6-bromo-4,5-dihydroxy-3-methoxycyclohex-2-enone	hemisynthesized from **7** for SAR studies		[45,100]
Compound (**23**)	cyclohexandiol	(1*S*,2*R*,5*R*,6*R*)-3-methoxy-7-oxabicyclo[4.1.0]hept-3-ene-2,5-diol	hemisynthesized from **7** for SAR studies		[45,100]
Compound (**24**)	cyclohexanone	(2*S*,4*R*,5*S*)-2,4-dihydroxy-5-methoxycyclohexanone	hemisynthesized from **7** for SAR studies		[45,100]
Compound (**25**)	cyclohexanone	(1*S*,2*S*,6*S*)-3-methoxy-5-oxo-7-oxabicyclo[4.1.0]hept-3-en-2-yl acetate	hemisynthesized from **8** for SAR studies	antioomycetes	[45,100]
Compound (**26**)	cyclohexanone	(1*R*,2*S*,3*S*)-4-methoxy-6-oxocyclohex-4-ene-1,2,3-triyl triacetate	hemisynthesized from **8** for SAR studies		[45,100]
Afritoxinone A (**27**)	pyranone	(3a*S*,6*R*,7a*S*)-6-methoxy-3,3a,6,7a-tetrahydro-2*H*-furo[2,3-b]pyran-2-one	*D. africana*	phytotoxic	[34]
Afritoxinone B (**28**)	pyranone	(3a*R*,6*R*,7a*S*)-6-methoxy-3,3a,6,7a-tetrahydro-2*H*-furo[2,3-b]pyran-2-one	*D. africana*	phytotoxic	[34]
Oxysporone (**29**)	pyranone	(3a*R*,4*S*,7a*R*)-4-hydroxy-3,3a,4,7a-tetrahydro-2*H*-furo[2,3-b]pyran-2-one	*D. africana*	phytotoxic; antioomycetes; antifungal	[34, 46]
R-(-)-Mellein (**30**)	isocoumarin	(*R*)-8-hydroxy-3-methylisochroman-1-one	*D. africana*	phytotoxicity	[34]
(3*R*,4*R*)-4-Hydroxymellein (**31**)	isocoumarin	(3*R*,4*R*)-4,8-dihydroxy-3-methylisochroman-1-one	*D. africana*	phytotoxicity	[34]
(3*R*,4*S*)-4-Hydroxymellein (**32**)	isocoumarin	(3*R*,4*S*)-4,8-dihydroxy-3-methylisochroman-1-one	*D. africana*	phytotoxicity	[34]
Compund (**33**)	pyranone	(3a*R*,4*S*,7a*R*)-2-oxo-3,3a,4,7a-tetrahydro-2*H*-furo[2,3-	hemisynthesized from		[46]

		b]pyran-4-yl acetate	**29** for SAR studies		
Compund (**34**)	pyranone	(3a*R*,4*S*,7a*R*)-2-oxohexahydro-2H-furo[2,3-b]pyran-4-yl acetate	hemisynthesized from **29** for SAR studies		[46]
Compund (**35**)	pyranone	(3a*R*,4*S*)-2-oxo-3,3a,4,7a-tetrahydro-2*H*-furo[2,3-b]pyran-4-yl 4-bromobenzoate	hemisynthesized from **29** for SAR studies	antioomycetes; antifungal	[46]
Compund (**36**)	pyranone	(3a*S*,7a*R*)-3,3a-dihydro-2*H*-furo[2,3-b]pyran-2,4(7a*H*)-dione	hemisynthesized from **29** for SAR studies		[46]
Compund (**37**)	pyranone	(3a*R*,4*S*,7a*R*)-4-hydroxyhexahydro-2*H*-furo[2,3-b]pyran-2-one	hemisynthesized from **29** for SAR studies		[46]
Compund (**38**)	pyrane	(S)-2-(4-hydroxy-3,4-dihydro-2*H*-pyran-5-yl)acetic acid	hemisynthesized from **29** for SAR studies		[46]
Compund (**39**)	pyrane	(*S*)-2-(4-hydroxy-4*H*-pyran-3-yl)acetic acid	hemisynthesized from **29** for SAR studies		[46]
Compund (**40**)	pyrane	(*S*)-methyl 2-(4-hydroxy-4*H*-pyran-3-yl)acetate	hemisynthesized from **29** for SAR studies		[46]
Ascochalasin (**41**)	cytochalasan	(3*S*,4*S*,6*S*,10*R*,14*R*,17a*R*)-3-benzyl-6,14-dihydroxy-4,10-dimethyl-5-methylene-3,3a,4,5,6,6a,9,10,11,12,13,14,15,16-tetradecahydro-1*H*-cyclotrideca[d]isoindole-1,17(2*H*)-dione	*Ascochyta heteromorpha*		[51]
Deoxaphomin (**42**)	cytochalasan	(3*S*,4*S*,6*S*,7*E*,10*R*,14*R*,15*E*,17a*R*)-3-benzyl-6-hydroxy-4,10,14-trimethyl-5-methylene-3,3a,4,5,6,6a,9,10,11,12,13,14-dodecahydro-1*H*-cyclotrideca[d]isoindole-1,17(2*H*)-dione	*A. heteromorpha*		[51]
Cytochalasins A (**43**)	cytochalasan	(3*S*,4*S*,6*S*,7E,10*R*,15*E*,18a*S*)-3-benzyl-6-hydroxy-4,10-dimethyl-5-methylene-3,3a,4,5,6,6a,10,11,12,13-decahydro-1*H*-[1]oxacyclotetradecino[2,3-*d*]isoindole-1,14,17(2*H*,9*H*)-trione	*A. heteromorpha*	antifungal, antibacterial	[51]
Cytochalasins B (**44**)	cytochalasan	(3*S*,4*S*,6*S*,7*E*,10*R*,14*R*,15*E*,18a*S*)-3-benzyl-6,14-dihydroxy-4,10-dimethyl-5-methylene-3,3a,4,5,6,6a,9,10,11,12,13,14-dodecahydro-1*H*-[1]oxacyclotetradecino[2,3-d]isoindole-1,17(2*H*)-dione	*A. heteromorpha*		[51]
Compound (**45**)	cytochalasan	(3*S*,4*S*,6*S*,7*E*,10*R*,14*R*,15*E*,18a*S*)-3-benzyl-4,10-dimethyl-5-methylene-1,17-dioxo-2,3,3a,4,5,6,6a,9,10,11,12,13,14,17-tetradecahydro-1*H*-[1]oxacyclotetradecino[2,3-d]isoindole-6,14-diyl diacetate	hemisynthesized from **44** for SAR studies		[51]
Compound (**46**)	cytochalasan	(3*S*,4*S*,6*S*,10*R*,14*R*,18a*S*)-3-benzyl-6,14-dihydroxy-4,10-dimethyl-5-methylene-3,3a,4,5,6,6a,9,10,11,12,13,14,15,16-tetradecahydro-1*H*-[1]oxacyclotetradecino[2,3-d]isoindole-1,17(2*H*)-dione	hemisynthesized from **44** for SAR studies		[51]

Compound (**47**)	cytochalasan	(3*S*,4*S*,6*S*,7*E*,10*R*,15*E*,18a*S*)-3-benzyl-4,10-dimethyl-5-methylene-1,14,17-trioxo-2,3,3a,4,5,6,6a,9,10,11,12,13,14,17-tetradecahydro-1*H*-[1]oxacyclotetradecino[2,3-d]isoindol-6-yl acetate	hemisynthesized from **43** for SAR studies	antibacterial	[51]
Cytochalasins C (**48**)	cytochalasan	(3*S*,6*S*,7*E*,10*S*,12*R*,13*E*,15*R*)-3-benzyl-6,12-dihydroxy-4,5,10,12-tetramethyl-1,11-dioxo-2,3,3a,6,6a,9,10,11,12,15-decahydro-1*H*-cycloundeca[d]isoindol-15-yl acetate	*A. heteromorpha*		[51]
Cytochalasins D (**49**)	cytochalasan	(3*S*,6*S*,7*E*,10*S*,12*R*,13*E*,15*R*)-3-benzyl-6,12-dihydroxy-4,5,10,12-tetramethyl-1,11-dioxo-2,3,3a,6,6a,9,10,11,12,15-decahydro-1*H*-cycloundeca[d]isoindol-15-yl acetate	*A. heteromorpha*		[51]
Cytochalasins E (**50**)	cytochalasan	(1*E*,4*S*,6*R*,7*E*,11a*S*,14*S*,15*S*,15a*R*,16a*S*)-14-benzyl-6-hydroxy-4,6,15,15a-tetramethyl-3,4,14,14a,15,15a,16a,16b-octahydro-[1,3]dioxacyclotridecino[4,5-d]oxireno[2,3-f]isoindole-5,10,12(6*H*,13*H*)-trione	*A. heteromorpha*		[51]
Cytochalasins H (**51**)	cytochalasan	(3*S*,6*S*,7*E*,10*S*,12*R*,13*E*,15*R*)-3-benzyl-6,12-dihydroxy-4,5,10,12-tetramethyl-1-oxo-2,3,3a,6,6a,9,10,11,12,15-decahydro-1*H*-cycloundeca[d]isoindol-15-yl acetate	*A. heteromorpha*		[51]
Cytochalasins J (**52**)	cytochalasan	(3*S*,6*S*,7*E*,10*S*,12*R*,13*E*,15*R*)-3-benzyl-6,12,15-trihydroxy-4,5,10,12-tetramethyl-2,3,3a,6,6a,9,10,11,12,15-decahydro-1*H*-cycloundeca[d]isoindol-1-one	*A. heteromorpha*		[51]
12β-Hydroxy-13α-methoxyverruculogen TR-2 (**53**)	indole diketopiperazine alkaloid	(5a*S*,6*S*,12*S*,14a*S*)-5a-hydroxy-12-(3-hydroxy-3-methylbutyl)-6,9-dimethoxy-1,2,3,5a,6,14a-hexahydropyrrolo[1'',2'':4',5']pyrazino[1',2':1,6]pyrido[3,4-b]indole-5,14(11*H*,12*H*)-dione	*Aspergillus fumigatus* LN-4		[52]
3-Hydroxyfumiquinazoline A (**54**)	fumiquinazoline	(1*R*,4*R*)-1-hydroxy-4-(((2*S*,9*S*,9a*S*)-9-hydroxy-2-methyl-3-oxo-2,3,9,9a-tetrahydro-1*H*-imidazo[1,2-a]indol-9-yl)methyl)-1-methyl-1*H*-pyrazino[2,1-b]quinazoline-3,6(2*H*,4*H*)-dione	*A. fumigatus* LN-4		[52]
Fumitremorgin C (**55**)	indole diketopiperazine alkaloid	(5a*R*,6*S*,12*S*,14a*S*)-5a,6-dihydroxy-9-methoxy-12-(3-methylbut-2-en-1-yl)-1,2,3,5a,6,14a-hexahydropyrrolo[1'',2'':4',5']pyrazino[1',2':1,6]pyrido[3,4-b]indole-5,14(11*H*,12*H*)-dione	*A. fumigatus* LN-4		[52]
Cyclotryprostatins A (**56**)	indole diketopiperazine alkaloid	(5a*S*,6*S*,12*S*,14a*S*)-5a,6-dihydroxy-9-methoxy-12-(3-methylbut-2-en-1-yl)-1,2,3,5a,6,14a-hexahydropyrrolo[1'',2'':4',5']pyrazino[1',2':1,6]pyrido[3,4-b]indole-5,14(11*H*,12*H*)-dione	*A. fumigatus* LN-4		[52]
Cyclotryprostatins B (**57**)	indole diketopiperazine alkaloid	(5a*S*,6*S*,12*S*,14a*S*)-5a-hydroxy-6,9-dimethoxy-12-(3-methylbut-2-en-1-yl)-1,2,3,5a,6,14a-	*A. fumigatus* LN-4		[52]

		hexahydropyrrolo[1'',2'':4',5']pyrazino[1',2':1,6]pyrido[3,4-b]indole-5,14(11*H*,12*H*)-dione			
Verruculogen TR-2 (**58**)	indole diketopiperazine alkaloid	(5a*R*,6*S*,12*S*,14a*S*)-5a,6-dihydroxy-12-(3-hydroxy-3-methylbutyl)-9-methoxy-1,2,3,5a,6,14a-hexahydropyrrolo[1'',2'':4',5']pyrazino[1',2':1,6]pyrido[3,4-b]indole-5,14(11*H*,12*H*)-dione	*A. fumigatus* LN-4		[64]
12β-Hydroxyverruculogen TR-2 (**59**)	indole diketopiperazine alkaloid	(5a*S*,6*S*,12*S*,14a*S*)-5a,6-dihydroxy-12-(3-hydroxy-3-methylbutyl)-9-methoxy-1,2,3,5a,6,14a-hexahydropyrrolo[1'',2'':4',5']pyrazino[1',2':1,6]pyrido[3,4-b]indole-5,14(11*H*,12*H*)-dione	*A. fumigatus* LN-4		[64]
Fumitremorgin B (**60**)	indole diketopiperazine alkaloid	(5a*R*,6*S*,12*S*,14a*S*)-5a,6-dihydroxy-9-methoxy-11-(3-methylbut-2-en-1-yl)-12-(2-methylprop-1-en-1-yl)-1,2,3,5a,6,14a-hexahydropyrrolo[1'',2'':4',5']pyrazino[1',2':1,6]pyrido[3,4-b]indole-5,14(11*H*,12*H*)-dione	*A. fumigatus* LN-4		[64]
Verruculogen (**61**)	indole diketopiperazine alkaloid	(5*R*,10*S*,10a*R*,14a*S*,15b*S*)-10,10a-dihydroxy-7-methoxy-2,2-dimethyl-5-(2-methylprop-1-en-1-yl)-1,10,10a,12,13,14,14a,15b-octahydro-3,4-dioxa-5a,11a,15a-triazacycloocta[lm]indeno[5,6-b]fluorene-11,15(2*H*,5*H*)-dione	*A. fumigatus* LN-4		[64]
Fumiquinazoline F (**62**)	fumiquinazoline	(1*R*,4*R*)-4-((1*H*-indol-2-yl)methyl)-1-methyl-1H-pyrazino[2,1-b]quinazoline-3,6(2*H*,4*H*)-dione	*A. fumigatus* LN-4		[64]
Fumiquinazoline G (**63**)	fumiquinazoline	(1*S*,4*R*)-4-((1*H*-indol-2-yl)methyl)-1-methyl-1*H*-pyrazino[2,1-b]quinazoline-3,6(2*H*,4*H*)-dione	*A. fumigatus* LN-4		[64]
Fumiquinazoline D (**64**)	fumiquinazoline	(1*S*,2a,1*S*,6b*S*,8*R*,15*R*)-6b-hydroxy-1,15-dimethyl-6b,7,8,15-tetrahydro-2a,8a,14,15a,16-pentaaza-8,15-ethanobenzo[4',5']pentaleno[1',6':4,5,6]cycloocta[1,2-b]naphthalene-2,9,17(1*H*,2a1*H*)-trione	*A. fumigatus* LN-4		[64]
Fumiquinazoline A (**65**)	fumiquinazoline	(1*S*,4*R*)-4-(((2*S*,9*S*,9a*S*)-9-hydroxy-2-methyl-3-oxo-2,3,9,9a-tetrahydro-1H-imidazo[1,2-a]indol-9-yl)methyl)-1-methyl-1H-pyrazino[2,1-b]quinazoline-3,6(2*H*,4*H*)-dione	*A. fumigatus* LN-4		[64]
Fusapyrone (**66**)	substituted pyrone	3-((2*R*,3*R*,4*R*,6*R*)-3,4-dihydroxy-6-(hydroxymethyl)tetrahydro-2H-pyran-2-yl)-4-hydroxy-6-((4*E*,6*E*,9*Z*)-3-hydroxy-8-(hydroxymethyl)-2,6,10,12-tetramethyloctadeca-4,6,9-trien-2-yl)-2*H*-pyran-2-one	*Fusarium semitectum*	antifungal	[53, 54]
Deoxyfusapyrone (**67**)	substituted pyrone	3-((2S,6S)-6-methyltetrahydro-2*H*-pyran-2-yl)-6-((4*E*,6*E*,9*Z*)-2,6,8,10,12-pentamethyloctadeca-4,6,9-trien-2-yl)-2*H*-pyran-2-one	hemisynthesized from **66** for SAR studies	high zootoxicity	[53, 54]
Compound (**68**)	substituted pyrone	(*Z*)-2-((1*E*,3*E*)-5-acetyl-6-(4-acetyl-3-((2*S*,4*R*,6*R*)-4-acetyl-6-(2-oxopropyl)tetrahydro-2*H*-pyran-2-yl)-2-	hemisynthesized from **66** for SAR studies		[55]

		oxo-2*H*-pyran-6-yl)-2,6-dimethylhepta-1,3-dien-1-yl)-4,6-dimethyldodec-3-en-1-yl acetate			
Compound (**69**)	substituted pyrone	4-acetyl-6-((4*E*,6*E*,9*Z*)-3-acetyl-2,6,8,10,12-pentamethyloctadeca-4,6,9-trien-2-yl)-3-((2*S*,4*R*,6*R*)-4-acetyl-6-(2-oxopropyl)tetrahydro-2H-pyran-2-yl)-2*H*-pyran-2-one	hemisynthesized from **67** for SAR studies		[55]
Compound (**70**)	substituted pyrone	(4*E*,6*E*)-3-(2-(4-acetyl-3-((2S,4*R*,6*S*)-4-acetyl-6-ethyltetrahydro-2*H*-pyran-2-yl)-2-oxo-2*H*-pyran-6-yl)propan-2-yl)-8-((*Z*)-2,4-dimethyldec-1-en-1-yl)-6-methylundeca-4,6-diene-2,10-dione	hemisynthesized from **66** for SAR studies		[55]
Compound (**71**)	substituted pyrone	(2*R*,3*R*,4*R*,6*R*)-2-(6-((4*E*,6*E*,9*Z*)-3-acetoxy-8-(acetoxymethyl)-2,6,10,12-tetramethyloctadeca-4,6,9-trien-2-yl)-2-methoxy-4-oxo-4*H*-pyran-3-yl)-6-(acetoxymethyl)tetrahydro-2*H*-pyran-3,4-diyl diacetate	hemisynthesized from **66** for SAR studies		[55]
Compound (**72**)	substituted pyrone	3-((2*S*,6*S*)-6-ethyltetrahydro-2*H*-pyran-2-yl)-4-hydroxy-6-((4*E*,6*E*,9*Z*)-8-(hydroxymethyl)-2,6,10,12-tetramethyloctadeca-4,6,9-trien-2-yl)-2*H*-pyran-2-one	hemisynthesized from **66** for SAR studies	weak activity against *B. cinerea*	[55]
Compound (**73**)	substituted pyrone	4-hydroxy-6-((4*E*,6*E*,9*Z*)-3-hydroxy-8-(hydroxymethyl)-2,6,10,12-tetramethyloctadeca-4,6,9-trien-2-yl)-3-(2-hydroxyethyl)-2*H*-pyran-2-one	hemisynthesized from **66** for SAR studies		[55]
Compound (**74**)	substituted pyrone	3-((2*R*,3*R*,4*R*,6*R*)-3,4-dihydroxy-6-(hydroxymethyl)tetrahydro-2H-pyran-2-yl)-4-hydroxy-6-((*Z*)-3-hydroxy-8-(hydroxymethyl)-2,6,10,12-tetramethyloctadec-9-en-2-yl)-2*H*-pyran-2-one	hemisynthesized from **66** for SAR studies	weak activity against *B. cinerea*	[55]
Compound (**75**)	substituted pyrone	3-((2*R*,3*R*,4*R*,6*R*)-3,4-dihydroxy-6-(hydroxymethyl)tetrahydro-2H-pyran-2-yl)-4-hydroxy-6-(3-hydroxy-8-(hydroxymethyl)-2,6,10,12-tetramethyloctadecan-2-yl)-2*H*-pyran-2-one	hemisynthesized from **66** for SAR studies	weak activity against *B. cinerea*	[55]
6-*n*-Pentyl-2*H*-pyran-2-one (**76**)	substituted pyrone	6-pentyl-2*H*-pyran-2-one	*Trichoderma* spp.	antifungal	[55,56]
Viridepyronone (**77**)	substituted pyrone	6-(4-oxopentyl)-2*H*-pyran-2-one	*Trichoderma viride*	antifungal	[57]
Fusaproliferin (**78**)	macrocyclic compound	(*S*)-2-((3a*S*,5*E*,9*Z*,13*R*,14*E*,16a*R*)-2,13-dihydroxy-3a,6,10,14-tetramethyl-3-oxo-3,3a,4,7,8,11,12,13,16,16a-decahydrocyclopenta[15]annulen-1-yl)propyl acetate	*Cleistothelebolus nipigonensis* and *Neogymnomyces virgineus*	antifungal	[58]
Terpestacin (**79**)	macrocyclic compound	(3a*R*,5*E*,7*R*,10*Z*,14*E*,16a*S*)-2,7-dihydroxy-3-((*S*)-1-hydroxypropan-2-yl)-6,10,14,16a-tetramethyl-3a,4,8,9,12,13,16,16a-octahydrocyclopenta[15]annulen-1(7*H*)-one	*C. nipigonensis* and *N. virgineus*	antifungal	[59]
Compound (**80**)	macrocyclic compound	(3a*R*,5*E*,7*R*,10*Z*,14*E*,16a*S*)-3-((*S*)-1-acetoxypropan-2-	hemisynthesized from		[59]

		yl)-6,10,14,16a-tetramethyl-1-oxo-1,3a,4,7,8,9,12,13,16,16a-decahydrocyclopenta[15]annulene-2,7-diyl diacetate	**78** for SAR studies		
Compound (**81**)	macrocyclic compound	(*S*)-2-((3a*S*,5*E*,9*Z*,13*R*,14*E*,16a*R*)-13-hydroxy-2-methoxy-3a,6,10,14-tetramethyl-3-oxo-3,3a,4,7,8,11,12,13,16,16a-decahydrocyclopenta[15]annulen-1-yl)propyl acetate	hemisynthesized from **78** for SAR studies		[59]
Compound (**82**)	macrocyclic compound	(2*S*)-2-((3a*S*,13*R*,16a*R*)-2,13-dihydroxy-3a,6,10,14-tetramethyl-3-oxo-3,3a,4,5,6,7,8,9,10,11,12,13,14,15,16,16a-hexadecahydrocyclopenta[15]annulen-1-yl)propyl acetate	hemisynthesized from **78** for SAR studies		[59]
Compound (**83**)	macrocyclic compound	(*S*)-2-((3a*S*,5*E*,9*Z*,13*R*,14*E*,16a*R*)-2-((1-(4-bromophenoxy)vinyl)oxy)-13-hydroxy-3a,6,10,14-tetramethyl-3-oxo-3,3a,4,7,8,11,12,13,16,16a-decahydrocyclopenta[15]annulen-1-yl)propyl acetate	hemisynthesized from **78** for SAR studies		[59]
Compound (**84**)	macrocyclic compound	(3a*R*,5*E*,7*R*,10*Z*,14*E*,16a*S*)-7-hydroxy-3-((*S*)-1-hydroxypropan-2-yl)-2-methoxy-6,10,14,16a-tetramethyl-3a,4,8,9,12,13,16,16a-octahydrocyclopenta[15]annulen-1(7*H*)-one	hemisynthesized from **79** for SAR studies		[59]
Compound (**85**)	macrocyclic compound	(3a*R*,7*R*,16a*S*)-2,7-dihydroxy-3-((*S*)-1-hydroxypropan-2-yl)-6,10,14,16a-tetramethyl-4,5,6,7,8,9,10,11,12,13,14,15,16,16a-tetradecahydrocyclopenta[15]annulen-1(3a*H*)-one	hemisynthesized from **79** for SAR studies	antifungal	[59]
Compound (**86**)	macrocyclic compound	*Bis*-4-bromophenyl ((*S*)-2-((3a*S*,5*E*,9*Z*,13*R*,14*E*,16a*R*)-2,13-dihydroxy-3a,6,10,14-tetramethyl-3-oxo-3,3a,4,7,8,11,12,13,16,16a-decahydrocyclopenta[15]annulen-1-yl)propyl) carbonate	hemisynthesized from **79** for SAR studies		[59]
Compound (**87**)	macrocyclic compound	4-bromophenyl ((3a*R*,5*E*,7*R*,10*Z*,14*E*,16a*S*)-2-hydroxy-3-((*S*)-1-hydroxypropan-2-yl)-6,10,14,16a-tetramethyl-1-oxo-1,3a,4,7,8,9,12,13,16,16a-decahydrocyclopenta[15]annulen-7-yl) carbonate	hemisynthesized from **79** for SAR studies	antifungal	[59]
Compound (**88**)	pimarane diterpene	(4a'*R*,4b'*R*,7'*R*,10a'*S*)-methyl 4b'-hydroxy-1',1',7'-trimethyl-10'-oxo-7'-vinyl-2',3',4',4a',4b',5',6',7',10',10a'-decahydro-1'*H*-spiro[oxirane-2,9'-phenanthrene]-4a'-carboxylate	hemisynthesized from **1** for SAR studies		[63]
Compound (**89**)	pimarane diterpene	(4a'*R*,4b'*R*,7'*R*,10a'*S*)-methyl 4b'-methoxy-1',1',7'-trimethyl-10'-oxo-7'-vinyl-2',3',4',4a',4b',5',6',7',10',10a'-decahydro-1'*H*-spiro[oxirane-2,9'-phenanthrene]-4a'-carboxylate	hemisynthesized from **1** for SAR studies		[63]
Compound (**90**)	pimarane diterpene	(2*S*,4a*R*,4b*R*,8a*S*,9*S*,10*R*)-2-ethyl-4a,9,10-trihydroxy-	hemisynthesized from **1**		[63]

		2,8,8-trimethyl-3,4,4a,5,6,7,8,8a,9,10-decahydro-2*H*-9,4b-(epoxymethano)phenanthren-12-one	for SAR studies		
Compound (**91**)	pimarane diterpene	(2*S*,4a*R*,4b*R*,8a*S*,9*S*,10*R*)-2-ethyl-4a,9-dihydroxy-2,8,8-trimethyl-12-oxo-3,4,4a,5,6,7,8,8a,9,10-decahydro-2H-9,4b-(epoxymethano)phenanthren-10-yl acetate	hemisynthesized from **1** for SAR studies		[63]
Compound (**92**)	pimarane diterpene	(2*S*,4a*R*,4b*R*,8a*S*,9*S*,10*R*)-2-ethyl-4a,9-dihydroxy-2,8,8-trimethyl-12-oxo-3,4,4a,5,6,7,8,8a,9,10-decahydro-2*H*-9,4b-(epoxymethano)phenanthren-10-yl acetate	hemisynthesized from **2** for SAR studies		[63]
Compound (**93**)	pimarane diterpene	(4a*R*,4b*R*,7*R*,10a*S*)-methyl 4b-hydroxy-1,1,7,8-tetramethyl-9,10-dioxo-7-vinyl-1,2,3,4,4a,4b,5,6,7,9,10,10a-dodecahydrophenanthrene-4a-carboxylate	hemisynthesized from **1** for SAR studies		[63]
Compound (**94**)	pimarane diterpene	(1*R*,1'*R*,2*S*,4'*R*)-methyl 1'-hydroxy-2-(2-hydroxyacetyl)-2',3,3,4'-tetramethyl-4'-vinyl-[1,1'-bi(cyclohexan)]-2'-ene-1-carboxylate	hemisynthesized from **2** for SAR studies		[63]
Fischerindoline (**95**)	pyrroloindole sesquiterpenoid	(2*S*,3a*R*,8a*R*)-(1*S*,2*R*,4a*R*,5*R*,8*R*,8a*R*)-2-acetoxy-8a-hydroxy-3,8-dimethyl-5-(prop-1-en-2-yl)-1,2,4a,5,6,7,8,8a-octahydronaphthalen-1-yl 3a-hydroxy-8-methyl-1,2,3,3a,8,8a-hexahydropyrrolo[2,3-b]indole-2-carboxylate	*Neosartorya pseudofischeri*		[66,67]
Eurochevalierine (**96**)	octahydronaphthalen	(*S*)-(1*S*,2*R*,4a*R*,5*R*,8*R*,8a*R*)-2-acetoxy-8a-hydroxy-3,8-dimethyl-5-(prop-1-en-2-yl)-1,2,4a,5,6,7,8,8a-octahydronaphthalen-1-yl 2-formamido-4-(2-(methylamino)phenyl)-4-oxobutanoate	*N. pseudofischeri*		[66,67]
Neosartins A (**97**)	dihydropyrazino[1,2-a]indole	3-methoxy-2,3-dimethyl-2,3-dihydropyrazino[1,2-a]indole-1,4-dione	*N. pseudofischeri*		[65]
Neosartins B (**98**)	dihydropyrazino[1,2-a]indole	3,3-dimethoxy-2-methyl-2,3-dihydropyrazino[1,2-a]indole-1,4-dione	*N. pseudofischeri*		[65]
Neosartins C (**99**)	dihydropyrazino[1,2-a]indole	2,3-dimethyl-2,3-dihydropyrazino[1,2-a]indole-1,4-dione	*N. pseudofischeri*		[65]
1,2,3,4-Tetrahydro-2,3-dimethyl-1,4-dioxopyrazino[1,2-a]indole (**100**)	dihydropyrazino[1,2-a]indole	2-methyl-3-methylene-2,3-dihydropyrazino[1,2-a]indole-1,4-dione	*N. pseudofischeri*	antibacterial	[65]
1,2,3,4-Tetrahydro-2-methyl- 3-methylene-1,4-dioxopyrazino[1,2-a]indole (**101**)	dihydropyrazino[1,2-a]indole	2-methylpyrazino[1,2-a]indole-1,3,4(2H)-trione	*N. pseudofischeri*		[65]
1,2,3,4-Tetrahydro-2-methyl-1,3,4- trioxopyrazino [1,2-a]	dihydropyrazino[1,2-a]indole	1,2,3,4-tetrahydro-2-methyl-1,3,4- trioxopyrazino [1,2-a] indole	*N. pseudofischeri*		[65]

indole (**102**)					
N-Methyl-1*H*-indole-2-carboxamide (**103**)	indole	*N*-methyl-1*H*-indole-2-carboxamide	*N. pseudofischeri*		[65]
Gliotoxin (**104**)	epidithiopyrazino[1,2-a]indole	6-hydroxy-3-(hydroxymethyl)-2-methyl-2,3,5a,6-tetrahydro-1*H*-3,10a-epidithiopyrazino[1,2-a]indole-1,4(10*H*)-dione	*N. pseudofischeri*	antibacterial	[65]
Acetylgliotoxin (**105**)	epidithiopyrazino[1,2-a]indole	3-(hydroxymethyl)-2-methyl-1,4-dioxo-2,3,4,5a,6,10-hexahydro-1*H*-3,10a-epidithiopyrazino[1,2-a]indol-6-yl acetate	*N. pseudofischeri*	antibacterial	[65]
Reduced gliotoxin (**106**)	hexahydropyrazino[1,2-a]indole	6-hydroxy-3-(hydroxymethyl)-3,10a-dimercapto-2-methyl-2,3,5a,6,10,10a-hexahydropyrazino[1,2-a]indole-1,4-dione	*N. pseudofischeri*	antibacterial	[65]
6-Acetylbis(methylthio)gliotoxin (**107**)	octahydropyrazino[1,2-a]indol	3-(hydroxymethyl)-2-methyl-3,10a-bis(methylthio)-1,4-dioxo-1,2,3,4,5a,6,10,10a-octahydropyrazino[1,2-a]indol-6-yl acetate	*N. pseudofischeri*		[65]
Bisdethiobis(methylthio) gliotoxin (**108**)	octahydropyrazino[1,2-a]indol	3-(hydroxymethyl)-2-methyl-3,10a-bis(methylthio)-1,4-dioxo-1,2,3,4,5a,6,10,10a-octahydropyrazino[1,2-a]indol-6-yl acetate	*N. pseudofischeri*		[65]
Didehydrobisdethiobis(methylthio)gliotoxin (**109**)	tetrahydropyrazino[1,2-a]indole	3-(hydroxymethyl)-2-methyl-3,10a-bis(methylthio)-2,3,10,10a-tetrahydropyrazino[1,2-a]indole-1,4-dione	*N. pseudofischeri*		[65]
Bis-*N*-norgliovictin (**110**)	bis(methylthio)piperazine	3-benzyl-6-(hydroxymethyl)-1-methyl-3,6-bis(methylthio)piperazine-2,5-dione	*N. pseudofischeri*		[65]
Pyripyropene A (**111**)	dodecahydrobenzo[f]pyrano[4,3-b]chromene	(3*S*,4*R*,4a*R*,6*S*,6a*S*,12*R*,12a*S*,12b*S*)-4-(acetoxymethyl)-12-hydroxy-4,6a,12b-trimethyl-11-oxo-9-(pyridin-3-yl)-1,2,3,4,4a,5,6,6a,11,12,12a,12b-dodecahydrobenzo[f]pyrano[4,3-b]chromene-3,6-diyl diacetate	*N. pseudofischeri*		[65]
Boydines A (**112**)	epipolythiodioxopiperazine	(4*S*,4a*S*,6a*R*,11*S*,11a*S*,13a*R*)-4,11-dihydroxy-6a,13a-bis(methylthio)-4,4a,6a,7,11,11a,13a,14-octahydropyrazino[1,2-a:4,5-a']diindole-6,13-dione	*Pseudallescheria boydii*		[68]
Boydines B (**113**)	epipolythiodioxopiperazine	(2*S*,3*R*,4*S*,*E*)-(4*S*,4a*S*,6a*R*,11*S*,11a*S*,13a*R*)-11-hydroxy-6a,13a-bis(methylthio)-6,13-dioxo-4,4a,6,6a,7,11,11a,13,13a,14-decahydropyrazino[1,2-a:4,5-a']diindol-4-yl 3-hydroxy-2,4,6-trimethyl-5-oxooct-6-enoate	*P. boydii*	antibacterial	[68]
Boydines C (**114**)	epipolythiodioxopiperazine	(2*S*,3*R*,4*S*,*E*)-(5*S*,5a*S*,7a*R*,12*S*,12a*S*,14a*R*)-12-hydroxy-7a,14a-bis(methylthio)-7,14-dioxo-5,5a,7,7a,8,12,12a,14,14a,15-decahydrooxepino[3'',4'':4',5']pyrrolo[1',2':4,5]pyrazino[1,2-a]indol-5-yl 3-hydroxy-2,4,6-trimethyl-5-oxooct-6-enoate	*P. boydii*		[68]
Boydines D (**115**)	epipolythiodioxopiperazine	(2*S*,3*R*,4*S*,*E*)-(5*S*,5a*S*,7a*R*,14a*R*)-12-hydroxy-7a,14a-	*P. boydii*		[68]

		bis(methylthio)-7,14-dioxo-5,5a,7,7a,8,14,14a,15-octahydrooxepino[3'',4'':4',5']pyrrolo[1',2':4,5]pyrazino[1,2-a]indol-5-yl 3-hydroxy-2,4,6-trimethyl-5-oxooct-6-enoate			
4a-*epi*-9α-Methoxydihydrodeoxybostrycin (**116**)	hydroanthraquinone	(2*R*,3*S*,4aS,9a*R*,10*R*)-2,3,5,8-tetrahydroxy-6,10-dimethoxy-3-methyl-1,3,4,4a,9a,10-hexahydroanthracen-9(2*H*)-one	*Nigrospora* sp.		[69]
10-Deoxybostrycin (**117**)	anthracene compound	(1*S*,2*R*,3*S*)-6-methoxy-3-methyl-1,2,3,4-tetrahydroanthracene-1,2,3,5,8,10-hexaol	*Nigrospora* sp.		[69]
Nigrosporin B (**118**)	anthracene compound	(6S,7*R*)-3-methoxy-6-methyl-5,6,7,8-tetrahydroanthracene-1,4,6,7,9-pentaol	*Nigrospora* sp.	antibacterial	[69]
9α-Hydroxydihydrodesoxybostrycin (**119**)	hydroanthraquinone	(2*R*,3*S*,4a*S*,9a*S*,10*R*)-2,3,5,8,10-pentahydroxy-6-methoxy-3-methyl-1,3,4,4a,9a,10-hexahydroanthracen-9(2*H*)-one	*Nigrospora* sp.		[69]
9α-Hydroxyhalorosellinia A (**120**)	hydroanthraquinone	(2*R*,3*S*,4a*R*,9a*R*,10*R*)-2,3,5,8,9a,10-hexahydroxy-6-methoxy-3-methyl-1,3,4,4a,9a,10-hexahydroanthracen-9(2*H*)-one	*Nigrospora* sp.		[69]
4-Deoxybostrycin (**121**)	hydroanthraquinone	(6*R*,7*S*)-2-methoxy-7-methyl-5,6,7,8-tetrahydroanthracene-1,4,6,7,9,10-hexaol	*Nigrospora* sp.		[69]
Bostrycin (**122**)	anthracene compound	(1*S*,2*R*,3*S*)-6-methoxy-3-methyl-1,2,3,4-tetrahydroanthracene-1,2,3,5,8,9,10-heptaol	*Nigrospora* sp.		[69]
Austrocortirubin (**123**)	anthraquinone	1,4,6-trihydroxy-2-methoxy-7-methylanthracene-9,10-dione	*Nigrospora* sp.		[69]
3,5,8-Trihydroxy-7-methoxy-2-methylanthracene-9,10-dione (**124**)	anthraquinone	1-hydroxy-3-methoxy-6-methylanthracene-9,10-dione	*Nigrospora* sp.		[69]
3-Acetoxy-4-deoxybostrycin (**125**)	anthracene compound	(2*R*,3*S*)-3,5,8,9,10-pentahydroxy-6-methoxy-3-methyl-1,2,3,4-tetrahydroanthracen-2-yl acetate	hemisynthesized from **121** for SAR studies	antibacterial	[69]
3-Acetoxybostrycin (**126**)	anthracene compound	(1*S*,2*R*,3*S*)-1,3,5,8,9,10-hexahydroxy-6-methoxy-3-methyl-1,2,3,4-tetrahydroanthracen-2-yl acetate	hemisynthesized from **122** for SAR studies		[69]
8-Acetoxy-3,5-dihydroxy-7-methoxy- 2-methylanthracene-9,10-dione (**127**)	anthraquinone	4,6-dihydroxy-2-methoxy-7-methyl-9,10-dioxo-9,10-dihydroanthracen-1-yl acetate	hemisynthesized from **124** for SAR studies		[69]
5-Acetoxy-3,8-dihydroxy-7-methoxy-2-methylanthracene-9,10-dione (**128**)	anthraquinone	4,7-dihydroxy-3-methoxy-6-methyl-9,10-dioxo-9,10-dihydroanthracen-1-yl acetate	hemisynthesized from **124** for SAR studies		[69]
3-Acetoxy-5,8-dihydroxy-7-methoxy-2-methylanthracene-9,10-dione (**129**)	anthraquinone	5,8-dihydroxy-6-methoxy-3-methyl-9,10-dioxo-9,10-dihydroanthracen-2-yl acetate	hemisynthesized from **124** for SAR studies		[69]
5,8-Diacetoxy-3-hydroxy- 7-methoxy-2-methylanthracene-9,10-dione (**130**)	anthraquinone	6-hydroxy-2-methoxy-7-methyl-9,10-dioxo-9,10-dihydroanthracene-1,4-diyl diacetate	hemisynthesized from **124** for SAR studies		[69]

3,8-Diacetoxy-5-hydroxy-7-methoxy-2-methylanthracene-9,10-dione (**131**)	anthraquinone	4-hydroxy-2-methoxy-7-methyl-9,10-dioxo-9,10-dihydroanthracene-1,6-diyl diacetate	hemisynthesized from **124** for SAR studies		[69]
3,5-Diacetoxy-8-hydroxy-7-methoxy-2-methylanthracene-9,10-dione (**132**)	anthraquinone	4-hydroxy-3-methoxy-6-methyl-9,10-dioxo-9,10-dihydroanthracene-1,7-diyl diacetate	hemisynthesized from **124** for SAR studies		[69]
3,5,8-Triacetoxy- -7-methoxy-2-methylanthracene-9,10-dione (**133**)	anthraquinone	2-methoxy-7-methyl-9,10-dioxo-9,10-dihydroanthracene-1,4,6-triyl triacetate	hemisynthesized from **124** for SAR studies		[69]
8-Acetoxyaustrocortirubin (**134**)	hydroanthraquinone	4-hydroxy-2-methoxy-7-methyl-9,10-dioxo-9,10-dihydroanthracen-1-yl acetate	hemisynthesized from **123** for SAR studies		[69]
Spiromastixones A (**135**)	depsidone analogue	3,8-dihydroxy-1,6-dipropyl-11*H*-dibenzo[b,e][1,4]dioxepin-11-one	*Spiromastix* sp.		[70]
Spiromastixones B (**136**)	depsidone analogue	2-chloro-3,8-dihydroxy-1,6-dipropyl-11*H*-dibenzo[b,e][1,4]dioxepin-11-one	*Spiromastix* sp.		[70]
Spiromastixones C (**137**)	depsidone analogue	4-chloro-3,8-dihydroxy-1,6-dipropyl-11*H*-dibenzo[b,e][1,4]dioxepin-11-one	*Spiromastix* sp.		[70]
Spiromastixones D (**138**)	depsidone analogue	4,7-dichloro-3,8-dihydroxy-1,6-dipropyl-11*H* dibenzo[b,e][1,4]dioxepin-11-one	*Spiromastix* sp.		[70]
Spiromastixones E (**139**)	depsidone analogue	2,4-dichloro-3,8-dihydroxy-1,6-dipropyl-11*H*-dibenzo[b,e][1,4]dioxepin-11-one	*Spiromastix* sp.		[70]
Spiromastixones F (**140**)	depsidone analogue	2,4,7-trichloro-3,8-dihydroxy-1,6-dipropyl-11*H*-dibenzo[b,e][1,4]dioxepin-11-one	*Spiromastix* sp.	antibacterial	[70]
Spiromastixones G (**141**)	depsidone analogue	2,4,7-trichloro-3-hydroxy-8-methoxy-1,6-dipropyl-11*H*-dibenzo[b,e][1,4]dioxepin-11-one	*Spiromastix* sp.	antibacterial	[70]
Spiromastixones H (**142**)	depsidone analogue	2,4,9-trichloro-3,8-dihydroxy-1,6-dipropyl-11*H*-dibenzo[b,e][1,4]dioxepin-11-one	*Spiromastix* sp.	antibacterial	[70]
Spiromastixones I (**143**)	depsidone analogue	2,4,7,9-tetrachloro-3,8-dihydroxy-1,6-dipropyl-11*H*-dibenzo[b,e][1,4]dioxepin-11-one	*Spiromastix* sp.	antibacterial	[70]
Spiromastixones J (**144**)	depsidone analogue	2,4,7,9-tetrachloro-3-hydroxy-8-methoxy-1,6-dipropyl-11*H*-dibenzo[b,e][1,4]dioxepin-11-one	*Spiromastix* sp.	antibacterial	[70]
Spiromastixones K (**145**)	depsidone analogue	2,4,7-trichloro-3-hydroxy-8-methoxy-1,9-dipropyl-11*H*-dibenzo[b,e][1,4]dioxepin-11-one	*Spiromastix* sp.		[70]
Spiromastixones L (**146**)	depsidone analogue	2,4,6,7-tetrachloro-3-hydroxy-8-methoxy-1,9-dipropyl-11*H*-dibenzo[b,e][1,4]dioxepin-11-one	*Spiromastix* sp.		[70]
Spiromastixones M (**147**)	depsidone analogue	2,4-dichloro-3,8-dihydroxy-1,9-dipropyl-11*H*-dibenzo[b,e][1,4]dioxepin-11-one	*Spiromastix* sp.		[70]
Spiromastixones N (**148**)	depsidone analogue	2,4,7-trichloro-3,8-dihydroxy-1,9-dipropyl-11*H*-dibenzo[b,e][1,4]dioxepin-11-one	*Spiromastix* sp.		[70]
Spiromastixones O (**149**)	depsidone analogue	2,4,6,7-tetrachloro-3,8-dihydroxy-1,9-dipropyl-11*H*-dibenzo[b,e][1,4]dioxepin-11-one	*Spiromastix* sp.		[70]
Cyclopaldic acid (**150**)	dihydroisobenzofuran	3,5-dihydroxy-7-methoxy-6-methyl-1-oxo-1,3-	*Seiridium cupressi*	insecticide activity	[78,80]

		dihydroisobenzofuran-4-carbaldehyde			
Compound (**151**)	dihydroisobenzofuran	7-methoxy-6-methyl-1-oxo-1,3-dihydroisobenzofuran-4-carboxylic acid	hemisynthesized from **150** for SAR studies		[78,80]
Compound (**152**)	dihydroisobenzofuran	7-formyl-6-hydroxy-4-methoxy-5-methyl-3-oxo-1,3-dihydroisobenzofuran-1-yl acetate	hemisynthesized from **150** for SAR studies		[78,80]
Compound (**153**)	dihydroisobenzofuran	(3,5-diacetoxy-7-methoxy-6-methyl-1-oxo-1,3-dihydroisobenzofuran-4-yl)methylene diacetate	hemisynthesized from **150** for SAR studies		[78,80]
Compound (**154**)	dihydroisobenzofuran	4-isobenzofurancarboxaldehyde, 1,3-dihydro-3,5-dihydroxy-7-methoxy-6-methyl-1-oxo-, 4-[2-(2,4-dinitrophenyl)hydrazone]	hemisynthesized from **150** for SAR studies		[78,80]
Compound (**155**)	dihydroisobenzofuran	1-naphthalenesulfonic acid, 5-(dimethylamino)-, 2-[(1,3-dihydro-3,5-dihydroxy-7-methoxy-6-methyl-1-oxo-4-isobenzofuranyl)methylene]hydrazide	hemisynthesized from **150** for SAR studies		[78,80]
Compound (**156**)	aromatic compound	benzoic acid, 2,3-bis[[2-[[5-(dimethylamino)-1-naphthalenyl]sulfonyl]hydrazinylidene]methyl]-4-hydroxy-6-methoxy-5-methyl-	hemisynthesized from **150** for SAR studies		[78,80]
Compound (**157**)	dihydroisobenzofuran	7-formyl-6-hydroxy-4-methoxy-5-methyl-3-oxo-1,3-dihydroisobenzofuran-1-yl 5-azidopentanoate	hemisynthesized from **150** for SAR studies		[78,80]
Compound (**158**)	dihydroisobenzofuran	5-hydroxy-7-methoxy-6-methyl-2a,4-dihydro-1*H*-2,3-dioxacyclopenta[cd]inden-1-one	hemisynthesized from **150** for SAR studies		[78,80]
Compound (**159**)	isobenzofuran	5-hydroxy-4-(hydroxymethyl)-7-methoxy-6-methylisobenzofuran-1(3*H*)-one	hemisynthesized from **150** for SAR studies		[78,80]
Seiridin (**160**)	furanone	(*R*)-4-(6-hydroxyheptyl)furan-2(5*H*)-one	*Seiridium cardinale*		[82]
2′-*O*-Acetylseiridin (**161**)	furanone	(*R*)-7-(4-methyl-5-oxo-2,5-dihydrofuran-3-yl)heptan-2-yl acetate	hemisynthesized from **160** for SAR studies		[82]
Compound (**162**)	furanone	4-((*R*)-6-hydroxyheptyl)-3-methyldihydrofuran-2(3H)-one	hemisynthesized from **160** for SAR studies		[82]
Compound (**163**)	furanone	3-methyl-4-(6-oxoheptyl)furan-2(5*H*)-one	hemisynthesized from **160** for SAR studies		[82]
Isoseiridin (**164**)	furanone	(*R*)-4-(5-hydroxyheptyl)furan-2(5*H*)-one	*S. cardinale*		[82]
Compound (**165**)	pimarane diterpene	4-azidobutyl ((2*R*,4a*R*,4b*R*,8a*S*,9*R*)-4a-hydroxy-2,8,8-trimethyl-10,12-dioxo-2-vinyl-3,4,4a,5,6,7,8,8a,9,10-decahydro-2*H*-9,4b-(epoxymethano)phenanthren-9-yl) carbonate	hemisynthesized from **1** for SAR studies		[78]
Compound (**166**)	pimarane diterpene	(2*R*,4a*R*,4b*R*,8a*S*,9*S*,10*R*)-4a-hydroxy-9,10-dimethoxy-2,8,8-trimethyl-2-vinyl-3,4,4a,5,6,7,8,8a,9,10-decahydro-2H-9,4b-(epoxymethano)phenanthren-12-one	hemisynthesized from **2** for SAR studies		[78]
Papyracillic acid (**167**)	dioxaspiro	(5*S*,7*R*,8*S*)-7-hydroxy-4-methoxy-7,8-dimethyl-9-methylene-1,6-dioxaspiro[4.4]non-3-en-2-one	*Ascochyta agropyrina* var. *nana*	mycoherbicide	[79,83, 84]
Compound (**168**)	furanone	3-methoxy-2-(3-methyl-4-oxopent-1-en-2-yl)-5-oxo-2,5-dihydrofuran-2-yl acetate	hemisynthesized from **167** for SAR studies		[84,85]

Compound (**169**)	furanone	(*Z*)-2-(3-methoxy-5-oxofuran-2(5*H*)-ylidene)-3-methyl-4-oxopentyl acetate	hemisynthesized from **167** for SAR studies		[84,85]
Compound (**170**)	furanone	(*E*)-2-(3-methoxy-5-oxofuran-2(5*H*)-ylidene)-3-methyl-4-oxopentyl acetate	hemisynthesized from **167** for SAR studies		[84,85]
Compound (**171**)	dioxaspiro	(5*S*,7*R*,8*S*,9*R*)-7-hydroxy-4,7,8,9-tetramethyl-1,6-dioxaspiro[4.4]non-3-en-2-one	hemisynthesized from **167** for SAR studies		[84,85]
Compound (**172**)	dioxaspiro	(5*S*,7*R*,8*S*)-4,7-dimethoxy-7,8-dimethyl-9-methylene-1,6-dioxaspiro[4.4]non-3-en-2-one	hemisynthesized from **167** for SAR studies		[84,85]
Compound (**173**)	dioxaspiro	(5*S*,7*R*,8*S*)-7-ethoxy-4-methoxy-7,8-dimethyl-9-methylene-1,6-dioxaspiro[4.4]non-3-en-2-one	hemisynthesized from **167** for SAR studies		[84,85]
Compound (**174**)	dimethylcyclobutyl	(2S)-2-(3-(heptyloxy)-2,3-dimethylcyclobut-1-en-1-yl)-3-methoxy-2,5-dihydrofuran-2,5-diol	hemisynthesized from **167** for SAR studies		[84,85]
Compound (**175**)	dimethylcyclobutyl	(2*R*)-2-(3-(heptyloxy)-2,3-dimethylcyclobut-1-en-1-yl)-3-methoxy-2,5-dihydrofuran-2,5-diol	hemisynthesized from **167** for SAR studies		[84,85]
Compound (**176**)	oxadiazine	(*E*)-methyl 3-methoxy-3-(5-((1*S*)-1-(2-methyloxiran-2-yl)ethyl)-4*H*-1,2,3-oxadiazin-6-yl)acrylate	hemisynthesized from **167** for SAR studies		[84,85]
Compound (**177**)	oxadiazetyl	(*E*)-methyl 3-methoxy-3-(3-((1*S*)-1-(4-methyl-4*H*-1,2,3-oxadiazet-4-yl)ethyl)-4,5-dihydrofuran-2-yl)acrylate	hemisynthesized from **167** for SAR studies		[84,85]
Compound (**178**)	oxadiazetyl	(*E*)-methyl 3-methoxy-3-(3-((1*R*)-1-(4-methyl-4*H*-1,2,3-oxadiazet-4-yl)ethyl)-4,5-dihydrofuran-2-yl)acrylate	hemisynthesized from **167** for SAR studies		[84,85]
Preaustinoid A (**179**)	meroterpenoids	(6a*R*,7*S*,9*R*,11*S*,12b*R*)-methyl 9-hydroxy-4,4,6a,9,11,12b-hexamethyl-13-methylene-3,8,10-trioxohexadecahydro-7,11-methanocycloocta[a]naphthalene-7-carboxylate	*Penicillium sp.*		[86-89]
Preaustinoid B (**180**)	meroterpenoids	(6a*R*,7*S*,9*R*,10*S*,11b*R*)-methyl 9-acetyl-9-hydroxy-4,4,6a,10,11b-pentamethyl-12-methylene-3,8-dioxotetradecahydro-1*H*-7,10-methanocyclohepta[a]naphthalene-7-carboxylate	*Penicillium sp.*		[86-89]
Preaustinoid A2 (**181**)	meroterpenoids	(7a*R*,8*S*,10*R*,12*S*,13b*R*)-methyl 10-hydroxy-5,5,7a,10,12,13b-hexamethyl-14-methylene-3,9,11-trioxo-3,5,5a,6,7,7a,8,9,10,11,12,13,13a,13b-tetradecahydro-8,12-methanocycloocta[3,4]benzo[1,2-c]oxepine-8-carboxylate	*Penicillium sp.*		[86-89]
Dehydroaustin (**182**)	meroterpenoids	(1*S*,3a*R*,3'*S*,5*R*,6*R*,10a*R*,11a*S*)-1,2',2',5-tetramethyl-4,7-dimethylene-3,6',12-trioxo-2',3,4,5,6,6a,6',7,9,10-decahydro-1*H*-spiro[5,11a-(epoxymethano)naphtho[1',8a':3,4]oxeto[2,3-c]furan-8,3'-pyran]-6-yl acetate	*Penicillium sp.*	larvicidal activities	[86-89]
Acetoxydehydroaustin (**183**)	meroterpenoids	(1*S*,3a*R*,3'*S*,5*R*,6*R*,10*S*,10a*R*,11a*S*)-1,2',2',5-	*Penicillium sp.*	larvicidal activities	[86-89]

		tetramethyl-4,7-dimethylene-3,6',12-trioxo-2',3,4,5,6,6a,6',7,9,10-decahydro-1*H*-spiro[5,11a-(epoxymethano)naphtho[1',8a':3,4]oxeto[2,3-c]furan-8,3'-pyran]-6,10-diyl diacetate			
Neoaustin (**184**)	meroterpenoids	(3*S*,3a*S*,3'*S*,5*S*,6a*R*,10a*R*,10b*S*)-3a-hydroxy-2',2',3,5,10a-pentamethyl-7,11-dimethylene-3,3a,5,6,6a,7,10,10a-octahydro-1*H*-spiro[5,10b-methanobenzo[3,4]cyclohepta[1,2-c]furan-8,3'-pyran]-1,4,6'(2'*H*,9*H*)-trione	*Penicillium sp.*		[86-89]
Austin (**185**)	meroterpenoids	(3S,3aS,3'S,6*R*,7*R*,11a*R*,11b*S*)-3a-hydroxy-2',2',3,6,8,11a-hexamethyl-12-methylene-1,4,6'-trioxo-2',3,3a,4,6,6',7,10,11,11a-decahydro-1*H*-spiro[6,11b-methanobenzo[e]furo[3,4-c]oxocine-9,3'-pyran]-7-yl acetate	*Penicillium sp.*		[86-89]
Okaramine A (**186**)	prenylated indole alkaloids	5*H*-indolo[3''',2''':4'',5'']azocino[1'',2'':4',5']pyrazino[1',2':1,5]pyrrolo[2,3-*b*]indole-10,18(6*H*,10a*H*)-dione, 16-(1,1-dimethyl-2-propen-1-yl)-11,11a,16,16a-tetrahydro-11a-hydroxy-6,6-dimethyl-, (10a*R*,11a*R*, 16a*S*)-	*Penicillium simplicissimum* AK-40 (ATCC 90288)		[87-90]
Okaramine B (**187**)	prenylated indole alkaloids	5*H*-azeto[1,2-*a*]indolo[3''',2''':4'',5'']azocino[1'',2'':4',5'] pyrazino[1',2':1,5]pyrrolo[2,3-*b*]indole-10,20(6*H*, 10a*H*)-dione, 11,11a,17,18-tetrahydro-10a,11a-dihydroxy-11-methoxy-6,6,17,17,18-pentamethyl-, (10a*S*,11*S*,11a*R*,18*R*,18a*S*)-	*P. simplicissimum* AK-40 (ATCC 90288)	insecticidal	[87-90]
Okaramine C (**188**)	prenylated indole alkaloids	2*H*-pyrazino[1',2':1,5]pyrrolo[2,3-*b*]indole-1,4(3*H*, 5a*H*)-dione, 6-(1,1-dimethyl-2-propen-1-yl)-3-[[2-(1, 1-dimethyl-2-propen-1-yl)-1*H*-indol-3-yl]methyl]-6, 10b,11,11a-tetrahydro-10b-hydroxy-, (3*S*,5a*R*,10b*S*, 11a*S*)-	*P. simplicissimum* AK-40 (ATCC 90288)		[87-90]
Okaramine G (**189**)	prenylated indole alkaloids	2*H*-Pyrazino[1',2':1,5]pyrrolo[2,3-*b*]indole-1,4(3*H*, 5a*H*)-dione, 6-(1,1-dimethyl-2-propen-1-yl)-3-[[2-(1, 1-dimethyl-2-propen-1-yl)-1*H*-indol-3-yl]methylene] -6,10b,11,11a-tetrahydro-10b-hydroxy-, (3*Z*,5a*S*, 10b*R*,11a*R*)-	*P. simplicissimum* AK-40 (ATCC 90288)		[87-90]
Okaramine H (**190**)	prenylated indole alkaloids	5*H*-indolo[3''',2''':4'',5'']azocino[1'',2'':4',5']pyrazino[1',2':1,5]pyrrolo[2,3-*b*]indole-10,18(6*H*,10a*H*)-dione, 11, 11a,16,16a-tetrahydro-11a-hydroxy-6,6-dimethyl-16-(3-methyl-2-buten-1-yl)-, (10a*R*,11a*R*,16a*S*)-	*P. simplicissimum* AK-40 (ATCC 90288)		[87-90]
Okaramine I (**191**)	prenylated indole alkaloids	5*H*-indolo[3''',2''':4'',5'']azocino[1'',2'':4',5']pyrazino[1',2':1,5]pyrrolo[2,3-*b*]indole-10,18(6*H*,10a*H*)-dione, 11, 11a,16,16a-tetrahydro-11a-hydroxy-6,6-dimethyl-, (10a*R*,11a*R*,16a*S*)-	*P. simplicissimum* AK-40 (ATCC 90288)		[87-90]

Okaramine N (**192**)	prenylated indole alkaloids	5*H*-indolo[3''',2''':4'',5'']azocino[1'',2'':4',5']pyrazino[1',2':1,5]pyrrolo[2,3-*b*]indole-10,18(6*H*,10a*H*)-dione, 16-(1,1-dimethyl-2-propen-1-yl)-11,11a,16,16a,18a,19-hexahydro-11a-hydroxy-6,6-dimethyl-, (10a*S*,11a*S*,16a*R*,18a*S*)-	*P. simplicissimum* AK-40 (ATCC 90288)		[87-90]
Okaramine Q (**193**)	prenylated indole alkaloids	5*H*-azeto[1,2-*a*]indolo[3''',2''':4'',5'']azocino[1'',2'':4',5'] pyrazino[1',2':1,5]pyrrolo[2,3-*b*]indole-10,20(6*H*,10a*H*)-dione, 11,11a,17,18-tetrahydro-10a,11a-dihydroxy-6,6,17,17,18-pentamethyl-, (10a*R*,11a*S*,18*S*,18a*R*)-	*P. simplicissimum* AK-40 (ATCC 90288)		[87-90]
2-Dehydroxy-3-demethoxy okaramine B (**194**)	prenylated indole alkaloids	5*H*-azeto[1,2-*a*]indolo[3''',2''':4'',5'']azocino[1'',2'':4',5'] pyrazino[1',2':1,5]pyrrolo[2,3-*b*]indole-10,20(6*H*,10a*H*)-dione, 11,11a,17,18-tetrahydro-11a-hydroxy-6,6,17,17,18-pentamethyl-, (10a*R*,11a*S*,18*S*,18a*R*)-	*P. simplicissimum* AK-40 (ATCC 90288)		[87-90]
Cyclo(N8-(α,α-dimethylallyl)-L-Trp-6a'-(α,α-dimethylallyl)-L-Trp) (**195**)	prenylated indole alkaloids	(3*S*,6*S*)-3-((1-(2-methylbut-3-en-2-yl)-1*H*-indol-3-yl)methyl)-6-((2-(2-methylbut-3-en-2-yl)-1*H*-indol-3-yl)methyl)piperazine-2,5-dione	*P. simplicissimum* AK-40 (ATCC 90288)		[87-90]
Chenopodolin (**196**)	pimarane diterpene	(1*S*,2*S*,5*S*,5a*S*,9*S*,10*S*,11a*R*,11b*S*,12*S*)-12-hydroxy-5,9,11b-trimethyl-4,6-dioxo-9-vinyl-1,2,4,5,5a,6,8,9,10,11,11a,11b-dodecahydro-2,5-methanonaphtho[1,2-d]oxepine-1,10-diyl diacetate	*Phoma chenopodiicola*	phytotoxicity	[91,92]
Stagonolide (**197**)	nonenolides	(9*R*,10*R*,*E*)-9-hydroxy-10-propyl-4,5,9,10-tetrahydro-2H-oxecine-2,8(3*H*)-dione	*P. chenopodiicola*		[93]
Putaminoxin (**198**)	nonenolides	(6*S*,*Z*)-6-hydroxy-10-propyl-3,4,5,6,9,10-hexahydro-2H-oxecin-2-one	*P. chenopodiicola*	phytotoxicity	[93]
Pinolidoxin (**199**)	nonenolides	(2*E*,4*E*)-(3*S*,8*R*,9*S*,10*R*,*E*)-8,9-dihydroxy-2-oxo-10-propyl-3,4,5,8,9,10-hexahydro-2H-oxecin-3-yl hexa-2,4-dienoate	*Stagonospora*, *Phoma* and *Ascochyta* spp	phytotoxicity	[93]
Cytochalasins F (**200**)	cytochalasan	(1E,4R,9E,12aS,15S,16S,16aR,17aS)-15-benzyl-4,16,16a-trimethyl-4,5,6,7,15,15a,16,16a-octahydro-3H-[1]oxacyclotetradecino[2,3-d]oxireno[2,3-f]isoindole-8,11,13(14H,17aH,17bH)-trione	*Stagonospora*, *Phoma* and *Ascochyta* spp		[93]
Cytochalasins T (**201**)	cytochalasan	(3*S*,4*S*,7*E*,10*R*,14*R*,15*E*,18a*R*)-3-benzyl-14-hydroxy-4,5,10-trimethyl-2,3,3a,4,9,10,11,12,13,14-decahydro-1*H*-[1]oxacyclotetradecino[2,3-d]isoindole-1,17(6a*H*)-dione	*Stagonospora*, *Phoma* and *Ascochyta* spp		[93]
Cytochalasins Z1 (**202**)	cytochalasan	(3*S*,4*S*,7*E*,10*R*,15*E*,18a*R*)-3-(4-hydroxybenzyl)-4,5,10-trimethyl-2,3,3a,4,9,10,11,12,13,14-decahydro-1*H*-[1]oxacyclotetradecino[2,3-d]isoindole-1,17(6aH)-dione	*Stagonospora*, *Phoma* and *Ascochyta* spp		[93]
Cytochalasins Z2 (**203**)	cytochalasan	(3*S*,4*S*,7*E*,10*R*,14*R*,15*E*,18a*R*)-3-benzyl-14-hydroxy-5-(hydroxymethyl)-4,10-dimethyl-	*Stagonospora*, *Phoma* and *Ascochyta* spp		[93]

		2,3,3a,4,9,10,11,12,13,14-decahydro-1*H*-[1]oxacyclotetradecino[2,3-d]isoindole-1,17(6a*H*)-dione			
Cytochalasins Z3 (**204**)	cytochalasan	(3*S*,4*S*,6*S*,7*E*,10*R*,15*E*,18a*S*)-3-benzyl-6,13-dihydroxy-4,10-dimethyl-5-methylene-3,3a,4,5,6,6a,9,10,11,12,13,14-dodecahydro-1*H*-[1]oxacyclotetradecino[2,3-d]isoindole-1,17(2*H*)-dione	*Stagonospora*, *Phoma* and *Ascochyta* spp		[93]
Agropyrenol (**205**)	substituted salicylaldehyde	2-((3R,4R,E)-3,4-dihydroxypent-1-en-1-yl)-6-hydroxybenzaldehyde	*A. agropyrina* var. *nana*		[94,95]
Phomentrioloxin (**206**)	cyclohex-5-ene-1,2,4-triol	(1*R*,2*R*,3*R*,4*R*)-3-methoxy-6-(7-methyl-3-methyleneoct-6-en-1-yn-1-yl)cyclohex-5-ene-1,2,4-triol	*Phomopsis* sp.		[96,97]
Fusicoccin (**207**)	diterpene	(*S*)-2-((1*S*,4*R*,5*R*,6*R*,6a*S*,9*S*,10a*R*,*E*)-4-(((2*S*,3*R*,4*S*,5*R*,6*R*)-4-acetoxy-3,5-dihydroxy-6-(((2-methylbut-3-en-2-yl)oxy)methyl)tetrahydro-2*H*-pyran-2-yl)oxy)-1,5-dihydroxy-9-(methoxymethyl)-6,10a-dimethyl-1,2,4,5,6,6a,7,8,9,10a-decahydrodicyclopenta[a,d][8]annulen-3-yl)propyl acetate	*Fusicoccum amygda*		[2,101]
Dideacetylfusicoccin A (**208**)	diterpene	(2*S*,3*R*,4*S*,5*S*,6*R*)-2-(((1*S*,4*R*,5*R*,6*R*,6a*S*,9*S*,10a*R*,*E*)-1,5-dihydroxy-3-((*S*)-1-hydroxypropan-2-yl)-9-(methoxymethyl)-6,10a-dimethyl-1,2,4,5,6,6a,7,8,9,10a-decahydrodicyclopenta[a,d][8]annulen-4-yl)oxy)-6-(((2-methylbut-3-en-2-yl)oxy)methyl)tetrahydro-2*H*-pyran-3,4,5-triol	hemisynthesized from **207** for SAR studies		[2,101]
Isopropylidene derivative of fusicoccin aglycone (**209**)	diterpene	(3a*R*,6*S*,6a*R*,8*S*,10a*S*,11*R*,11a*R*,*E*)-4-((*S*)-1-hydroxypropan-2-yl)-8-(methoxymethyl)-2,2,6a,11-tetramethyl-3a,5,6,6a,8,9,10,10a,11,11a-decahydrodicyclopenta[3,4:6,7]cycloocta[1,2-d][1,3]dioxol-6-ol	hemisynthesized from **207** for SAR studies		[2,101]
16-*O*-Demethyl-de-*tert*-pentenylfusicoccin A (**210**)	diterpene	(2*R*,3*S*,4*R*,5*R*,6*S*)-6-(((1*S*,4*R*,5*R*,6*R*,6a*S*,11a*R*,*Z*)-1,5,9-triacetoxy-3-((S)-1-acetoxypropan-2-yl)-6,11a-dimethyl-2,4,5,6,6a,7,8,9,10,11a-decahydro-1*H*-benzo[a]cyclopenta[d][8]annulen-4-yl)oxy)tetrahydro-2*H*-pyran-2,3,4,5-tetrayl tetraacetate	hemisynthesized from **207** for SAR studies		[102]
16-*O*-Demethyl-de-*tert*-pentenylfusicoccin A (**211**)	diterpene	(2*R*,3*R*,4*S*,5*R*,6*S*)-2-(acetoxymethyl)-6-(((1*S*,4*R*,5*R*,6*R*,6a*S*,11a*R*,*Z*)-1,5-diacetoxy-3-((S)-1-acetoxypropan-2-yl)-6,11a-dimethyl-2,4,5,6,6a,7,8,11a-octahydro-1*H*-	hemisynthesized from **207** for SAR studies		[102]

		benzo[a]cyclopenta[d][8]annulen-4-yl)oxy)tetrahydro-2*H*-pyran-3,4,5-triyl triacetate			
Compound (**212**)	cyclohexanone	(1*S*,2*R*,6*S*)-3-methoxy-5-oxo-7-oxabicyclo[4.1.0]hept-3-en-2-yl 5-azidopentanoate	hemisynthesized from **7** for SAR studies		[100]
Compound (**213**)	cyclohexanone	(1*S*,2*R*,6*S*)-3-methoxy-5-oxo-7-oxabicyclo[4.1.0]hept-3-en-2-yl 4-bromobenzoate	hemisynthesized from **7** for SAR studies		[100]
Compound (**214**)	cyclohexanone	(1*S*,2*S*,6*S*)-3-methoxy-5-oxo-7-oxabicyclo[4.1.0]hept-3-en-2-yl 5-azidopentanoate	hemisynthesized from **8** for SAR studies		[100]
Compound (**215**)	cyclohexanone	(1*S*,2*S*,5*R*,6*R*)-3-methoxy-7-oxabicyclo[4.1.0]hept-3-ene-2,5-diol	hemisynthesized from **8** for SAR studies		[100]
Cochliotoxin (**216**)	dihydropyranopyrandione	(2*S*,3*S*)-3-hydroxy-2-methyl-7-(3-methyloxiran-2-yl)-2,3-dihydropyrano[4,3-b]pyran-4,5-dione	*Cochliobolus australiensis*	phytotoxicity	[106–108]
Radicinin (**217**)	dihydropyranopyrandione	(2*S*,3*S*)-3-hydroxy-2-methyl-7-((*E*)-prop-1-en-1-yl)-2,3-dihydropyrano[4,3-b]pyran-4,5-dione	*C. australiensis*	phytotoxicity	[106–108]
3-*epi*-Radicinin (**218**)	dihydropyranopyrandione	(2*S*,3*R*,4*S*)-3,4-dihydroxy-2-methyl-7-((*E*)-prop-1-en-1-yl)-3,4-dihydropyrano[4,3-b]pyran-5(2*H*)-one	*C. australiensis*	phytotoxicity	[106–108]
Radicinol (**219**)	dihydropyranopyrandione	(2*S*,3*R*)-3-hydroxy-2-methyl-7-((*E*)-prop-1-en-1-yl)-2,3-dihydropyrano[4,3-b]pyran-4,5-dione	*C. australiensis*		[106–108]
3-*epi*-Radicinol (**220**)	dihydropyranopyrandione	(2*S*,3*S*,4*S*)-3,4-dihydroxy-2-methyl-7-((*E*)-prop-1-en-1-yl)-3,4-dihydropyrano[4,3-b]pyran-5(2*H*)-one	*C. australiensis*		[106–108]
Chloromonilinic acid B (**221**)	chromanonacrylic acid	(*E*)-3-chloro-3-(5-hydroxy-3-(2-methoxy-2-oxoethyl)-7-methyl-4-oxo-4*H*-chromen-2-yl)acrylic acid	*C. australiensis*	phytotoxicity	[106–108]
Chloromonilinic acids C (**222**)	chromanonacrylic acid	(*S*,*E*)-3-chloro-3-(5-hydroxy-3-(1-hydroxy-2-methoxy-2-oxoethyl)-7-methyl-4-oxo-4*H*-chromen-2-yl)acrylic acid	*C. australiensis*	phytotoxicity	[106–108]
Chloromonilinic acids D (**223**)	chromanonacrylic acid	(*Z*)-3-chloro-3-(5-hydroxy-3-(2-methoxy-2-oxoethyl)-7-methyl-4-oxo-4*H*-chromen-2-yl)acrylic acid	*C. australiensis*	phytotoxicity	[106–108]
Chloromonilicin (**224**)	oxepinochromene	(*S*)-methyl 5-chloro-10-hydroxy-8-methyl-3,11-dioxo-3,11-dihydro-1*H*-oxepino[4,3-b]chromene-1-carboxylate	*C. australiensis*	phytotoxicity	[106–108]
